# Population segmentation of type 2 diabetes mellitus patients and its clinical applications - a scoping review

**DOI:** 10.1186/s12874-021-01209-w

**Published:** 2021-03-11

**Authors:** Jun Jie Benjamin Seng, Amelia Yuting Monteiro, Yu Heng Kwan, Sueziani Binte Zainudin, Chuen Seng Tan, Julian Thumboo, Lian Leng Low

**Affiliations:** 1 Duke-NUS Medical School, 8 College Road, Singapore, 169857 Singapore; 2 SingHealth Regional Health System PULSES Centre, Singapore Health Services, Outram Rd, Singapore, 169608 Singapore; 3 Program in Health Services and Systems Research, Duke-NUS Medical School, 8 College Road, Singapore, 169857 Singapore; 4 Department of Pharmacy, Faculty of Science, National University of Singapore, Singapore, Singapore; 5 Department of General Medicine (Endocrinology), Sengkang General Hospital, Singapore, Singapore; 6 Saw Swee Hock School of Public Health, National University of Singapore and National University Health System, Singapore, Republic of Singapore; 7 Department of Rheumatology and Immunology, Singapore General Hospital, Singapore, Singapore; 8 SingHealth Regional Health System, Singapore Health Services, Singapore, Singapore; 9 Department of Family Medicine and Continuing Care, Singapore General Hospital, Outram Road, Singapore, 169608 Singapore; 10 SingHealth Duke-NUS Family Medicine Academic Clinical Program, Singapore, Singapore; 11 Outram Community Hospital, SingHealth Community Hospitals, 10 Hospital Boulevard, Singapore, 168582 Singapore

**Keywords:** Diabetes mellitus, type 2, Cluster analysis, Latent class analysis, Population segmentation, Data analysis, Patient outcome assessment, Outcome assessment, health care, Scoping review

## Abstract

**Background:**

Population segmentation permits the division of a heterogeneous population into relatively homogenous subgroups. This scoping review aims to summarize the clinical applications of data driven and expert driven population segmentation among Type 2 diabetes mellitus (T2DM) patients.

**Methods:**

The literature search was conducted in Medline®, Embase®, Scopus® and PsycInfo®. Articles which utilized expert-based or data-driven population segmentation methodologies for evaluation of outcomes among T2DM patients were included. Population segmentation variables were grouped into five domains (socio-demographic, diabetes related, non-diabetes medical related, psychiatric / psychological and health system related variables). A framework for PopulAtion Segmentation Study design for T2DM patients (PASS-T2DM) was proposed.

**Results:**

Of 155,124 articles screened, 148 articles were included. Expert driven population segmentation approach was most commonly used, of which judgemental splitting was the main strategy employed (*n* = 111, 75.0%). Cluster based analyses (*n* = 37, 25.0%) was the main data driven population segmentation strategies utilized. Socio-demographic (*n* = 66, 44.6%), diabetes related (*n* = 54, 36.5%) and non-diabetes medical related (*n* = 18, 12.2%) were the most used domains. Specifically, patients’ race, age, Hba1c related parameters and depression / anxiety related variables were most frequently used. Health grouping/profiling (*n* = 71, 48%), assessment of diabetes related complications (*n* = 57, 38.5%) and non-diabetes metabolic derangements (*n* = 42, 28.4%) were the most frequent population segmentation objectives of the studies.

**Conclusions:**

Population segmentation has a wide range of clinical applications for evaluating clinical outcomes among T2DM patients. More studies are required to identify the optimal set of population segmentation framework for T2DM patients.

**Supplementary Information:**

The online version contains supplementary material available at 10.1186/s12874-021-01209-w.

## Background

### Rationale and objective

The global disease burden for type 2 diabetes mellitus (T2DM) is rising, with projected healthcare expenditures incurred by governments worldwide to exceed U.S.$ 2.3 trillion by 2030 [1]. Despite the advent of new drug therapeutics and improvement in diabetic care processes, the management of T2DM remains suboptimal and it remains one of the leading causes for non-traumatic lower extremity amputations, blindness and end-stage renal disease requiring renal replacement therapy [2].

With the rising prevalence of T2DM and its associated healthcare costs, the development and delivery of healthcare models from a population health perspective are becoming increasingly relevant. Population health refers to “the health outcomes of a group of individuals, including the distribution of such outcomes within the group” [3]. Within the field of population health analytics, population segmentation forms an important pillar where a data-driven segregation approach applied to a heterogeneous population cohort can generate meaningful and relatively homogenous sub-groups with similar healthcare needs [4]. This in turn allows healthcare administrators to navigate large and complex databases efficiently and synthesize essential patient factors which contribute to the health related outcome of interest such as healthcare utilization [5].

There are two distinctive approaches to population segmentation which are namely expert-driven and data-driven approaches. The derivation of patient segments using expert-driven approach is pre-determined by an expert panel (e.g. judgemental splits or prescribed binning criteria), while data-driven approaches perform specialized statistical techniques such as latent class analysis (LCA) on a dataset to derive the patient segments [6]. An example of an expert-driven framework is the “Bridges to Health” model which divides a patient population into eight segments comprising of patients without health issues to dying patients with rapid deterioration [7]. It has been suggested as an aid to guide the planning and allocation of healthcare resources tailored for each patient segment [7]. On the other hand, data-driven approaches have been used to profile patient segments by their healthcare utilization and clinical outcomes. An example is a study by Yan et al. which utilized LCA to identify six classes of primary care utilizers with differential healthcare utilization and mortality [8].

Among T2DM patients, data-driven population segmentation methodologies have also been leveraged to identify subgroups of patients with differential risk of diabetes related complications, healthcare utilization and clinical trajectories in large administrative patient databases [9]. A study by Jiang et al. identified four unique profiles of patients where patients in the “high morbidity / moderate treatment” group was shown to have the highest rates of inpatient admissions, all-cause healthcare costs and risk for diabetic nephropathy progression [10]. Another study by Karpati et al. derived three clusters of patients with differing Hba1c trajectories, where patients in both increasing and decreasing Hba1c trajectory clusters were to have higher prevalence of microvascular and macrovascular complications [11].

While reviews have summarized the applications of use-based [10] and healthcare needs based population segmentation [5] among general patient populations, there is no review which has evaluated the clinical applications of population segmentation among T2DM patients. It is important to note that T2DM patients form a high-priority target patient population as the comprehensive coverage and optimization of diabetes care involve a constellation of psychosocial, economic and demographical determinants, and requires a multi-pronged approach ranging from disease maintenance to prevention of its complications. Notably, care models designed for T2DM often serve as a model for the management of other chronic diseases. Coupled with the high prevalence of T2DM and its implications on the development of multi-organ complications, this makes T2DM patients highly amendable to reap the benefits of population segmentation so as to optimise patient outcomes. Hence, we aim to summarize the literature on the clinical applications of population segmentation among T2DM patients.

## Methods

A scoping review was conducted for studies which applied the use of population segmentation techniques among T2DM patients and was reported using the Preferred Reporting Items for Systematic review and Meta-Analysis extension for Scoping Reviews (PRISMA-ScR) checklist [12].

### Protocol and registration

The protocol for the search strategy was registered on Open Science Framework (10.17605/osf.io/ay6uc).

### Eligibility criteria and information sources

The literature search was performed in Medline®, Embase®, SCOPUS® and PsycInfo®. We included peer-reviewed studies in English language which applied data-driven or expert-driven approaches population segmentation among adult patients (age ≥ 18 years old) with T2DM. We excluded studies that included patients with type 1 diabetes mellitus or maturity onset diabetes of the young, as well as articles that were not in the English language. Randomized controlled trials, cross-sectional, case-control, cohort and record linkage studies were included. Case studies, case series, meta-analyses and other reviews were excluded. In situations where the subtype of diabetic patients studied was not clearly specified, we contacted the authors of the study for clarifications. The search was current as of September 2019. As this review did not include human subjects, institutional review board approval was exempted.

### Search, selection of sources of evidence, data charting process and data items

The search terms included concepts and strategies utilized to segregate patients in population segmentation which were adapted from a review by Yan et al. [10], and key T2DM related terms. The details of the full search strategy are listed in Supplementary File 1. A pilot exercise for the screening of articles was performed by two independent reviewers (SJJB and AM) for the first 200 records (based on title and abstract). Thereafter, the same reviewers screened the titles and abstracts of all retrieved articles. After a second pilot exercise to screen the first 20 full-text articles, the full-texts of identified articles were evaluated by SJJB and AM independently for inclusion in the review. All disagreements in the inclusion process were discussed to reach a consensus. In the event that discrepancies could not be resolved, discussion with a third independent reviewer (YHK) was performed. Hand-searching of references in included articles was conducted.

The references and abstracts identified from the literature search were pooled in EndNote X9 software, which was utilized to remove the duplicated references. The removal of duplicated references was performed using the automated function in Endnote X9 and manual screening thereafter. Screening of the title, abstract and full-text was performed using a standardized Microsoft Excel spreadsheet which contained checkboxes for each inclusion and exclusion criteria. Conflicts during the screening process were automatically flagged by the software, using formulas embedded within the spreadsheet. All members of the research team involved in the screening of articles were trained to use the screening form. Thereafter, data of included articles were extracted independently by the two reviewers into a separate standardized Microsoft Excel spreadsheet. This information included the study’s title, publication year, sample size and characteristics of patient population, objectives of population segmentation, variables used for segmentation of patients, number/categories of patient segments derived and funding sources. In addition, the funding sources of included studies were extracted and reported, as per recommendations from AMSTAR-2 [13]. The full list of variables collected are reported in Supplementary Files 2 and 3.

### Critical appraisal of individual sources of evidence

Critical appraisal of the risk of bias for included studies was not performed as this was not the objective of this scoping review.

### Summary and synthesis of results

Descriptive statistics were used to summarize the characteristics of included studies which encompassed the study design, population, segmentation methods and variables used. The results were tabulated or presented in graphical charts to map the literature. A narrative summary of the population segmentation methodologies and variables used was presented. The segmentation methods identified from each study were mapped into two key themes - data-driven and expert-driven approaches [14]. Prescribed binning and judgemental splitting are examples of expert-driven population segmentation strategies. Prescribed binning utilizes a set of “off-the-shelf” binning rules, which are pre-determined by experts to divide a patient cohort into pre-defined segments [14]. On the other hand, judgemental splitting segregates patients based on one or more explanatory variables, which is determined by the judgement or documented experiences of healthcare practitioners or experts [14]. With regards to data-driven strategies, they can be grouped into decision trees and cluster-based segmentation [14]. Decisions trees utilize an objective classification strategy where patients are divided at successive decision nodes containing the explanatory variables and mimic the extension of branches in a tree [14]. An example is Breiman’s Classification and Regression Tree (CART) model which employs a binary recursive partitioning algorithm on the covariate space of a patient cohort [15]. Lastly, cluster based segmentation refer to a group of unsupervised modelling methods such as k-means and LCA which seeks to identify homogenous subgroups within a population.

Variables used for population segmentation were mapped into five broad domains which encompassed socio-demographic, diabetes related, non-diabetes medical related, psychological or psychological related and healthcare systems related variables.

#### Proposed framework for design of population segmentation studies for T2DM patients (PASS-T2DM)

A proposed framework, PopulAtion Segmentation Studies design framework for T2DM patients (PASS-T2DM), was constructed using population segmentation variables and outcomes identified from the review. The study design framework was divided into three phases: 1) Selection of study design; 2) Selection of population segmentation outcomes and population segmentation variables and 3) Evaluation of segments generated. The approaches to selection of study designs for population segmentation studies were divided into data-driven and expert driven approaches. The advantages and disadvantages of the approaches were listed in Supplementary File 4. With regards to the population segmentation variables, variables identified were categorized into three categories which were namely: Category A: important and accessible variables; Category B: important variables that are relatively accessible and Category C: important variables that may not be readily accessible. The derivation of these categories factored in the level of accessibility and importance of the variables where a variable is important is determined by its clinical relevance and need/usefulness in diabetic care as assessed by existing literature and expert opinion. The relative level of accessibility was determined from a health system perspective. The selection and assignment of variables to each category was discussed among population segmentation experts (LLL, TCS, JT) and endocrinologist (SBZ) within the team. All disagreements were resolved via further discussion to achieve consensus. With regards to the evaluation of segments generated, important criteria utilized commonly in consumer market segmentation and a review by Yan et al. was adapted for use [10, 16]. They were namely number of patient segments, internal and external validation, identifiability and interpretability, substantiality, stability and actionability. (Definitions in Supplementary File 5).

#### Data availability statement

All data analyzed in this study are included within the published article and its supplementary information files.

## Results

### Selection of sources of evidence

**Fig. 1:**
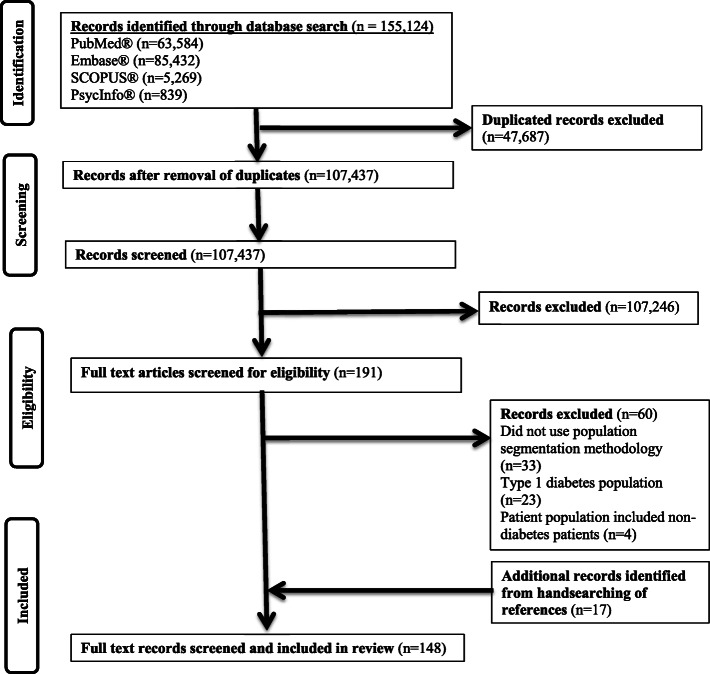
Flowchart for retrieval of articles

Figure 1 shows the flowchart for inclusion and exclusion of articles. After the exclusion of duplicated and irrelevant articles and, inclusion of 17 articles from hand-searching, a total of 148 articles were included in this review. The overall percentage of agreement between SJJB and AM during the screening of articles was 89.2% and all disagreements were resolved after discussion. Detailed information on the characteristics of individual studies is reported in Supplementary File 4. Thirty-seven studies (25%) received partial or full funding by private organizations while 47 studies (31.8%) were not funded. (Supplementary File 3). The remaining studies (*n* = 64, 43.2%) were funded by governmental agencies, professional organizations and/or research foundations.

### Characteristics of sources of evidence

Table 1 shows the characteristics of the studies included in the review. Majority of the studies were conducted between 2011 and 2019 (*n* = 113, 76.4%) and in Asia (*n* = 58, 39.2%), Europe (*n* = 40, 27.0%) and North America (n = 40, 27.0%). Among these continents, the United States of America (USA) (*n* = 33, 22.3%) [10, 17–47], China (*n* = 17, 11.5%) [48–63] and Italy (n = 11, 7.4%) [64–73] were the three countries with the highest number of studies. The two most common study designs employed were cross sectional studies (*n* = 92, 62.1%) and cohort studies (*n* = 50, 33.8%). Most studies included fewer than 5000 patients (*n* = 101, 68.2%).

**Table 1:** Characteristics of included studies (n = 148)

Characteristics of studies	N (%)
***Year of study***	
Before Year 2000	3 (2.0)
Year 2000–2010	32 (21.6)
Year 2011–2020	113 (76.4)
***Continent of study***	
Asia	58 (39.2)
Europe	40 (27.0)
North America	40 (27.0)
Australia	3 (2.0)
South America	3 (2.0)
Cross-continents	4 (2.7)
***Country of study***	
USA	33 (22.3)
China	17 (11.5)
Italy	11 (7.4)
Canada	6 (4.1)
Netherlands	6 (4.1)
Korea	6 (4.1)
Singapore	5 (3.4)
Australia	3 (2.0)
Hong Kong	4 (2.7)
Japan	4 (2.7)
Multi-countries	5 (3.4)
Others	48 (32.4)
***Study design***	
Cross-sectional study	92 (62.1)
Cohort study	50 (33.8)
Prospective cohort study	21 (14.2)
Retrospective cohort study	29 (19.5)
Record linkage study	2 (1.4)
Prospective record linkage study	1 (0.7)
Retrospective record linkage study	1 (0.7)
Randomized controlled trials	4 (2.7)
***Patient population***	
≤ 500	37 (25)
501–1000	21 (14.2)
1001 – 5000	43 (29.1)
5001 – 10,000	11 (7.4)
10,001–50,000	17 (11.5)
50,001–100,000	7 (4.7)
100,001–500,000	9 (6.1)
500,000 – 1000,000	2 (1.4)
> 1000,000	1 (0.7)
***Type 2 diabetes subgroups***	
Adult diabetics (across all age ranges)	137 (92.6)
Elderly (≥ 65 years old)	8 (5.4)
Young (≤ 40 years old)	2 (1.4)
Women with singleton pregnancy	1 (0.7)
***Study setting (Healthcare)***^**a**^	
Primary	40 (27.0)
Tertiary	68 (45.9)
Mixed	10 (6.8)
Not applicable or not specified	30 (20.3)
***Data source***	
Primary	70 (47.3)
Secondary	76 (51.4)
Mixed (Primary and Secondary)	2 (1.4)

^a^ – There were no studies performed in secondary healthcare settings

With regards to the subgroups of T2DM patients studied, adult T2DM patients (without age restriction) formed the most studied population (*n* = 137, 92.6%). Eight studies (5.4%) focused on elderly T2DM patients (≥65 years old) [24, 30, 32, 62, 74–77] while two studies (1.4%) [78, 79] focused on young T2DM patients (≤40 years old). Majority of the studies were conducted in tertiary healthcare settings (*n* = 68, 45.9%) and utilized secondary data sources (*n* = 76, 51.4%).

### Synthesis of results

#### Population segmentation strategies utilized in studies

**Fig. 2:**
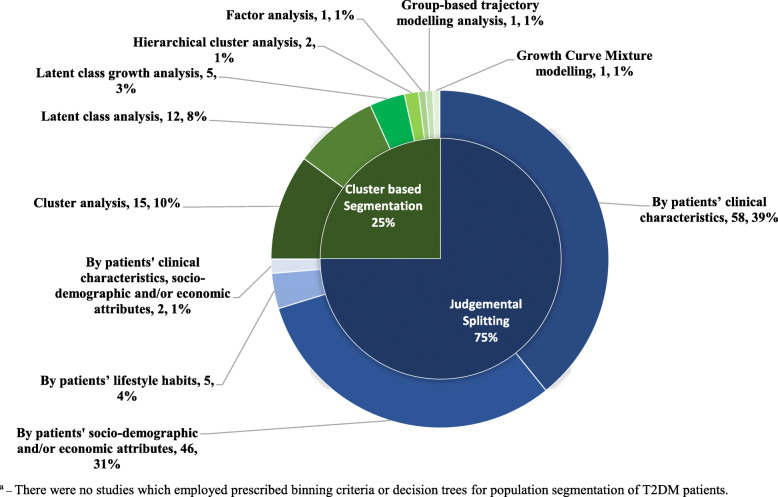
Population segmentation strategies employed in studies (*n* = 148) ^a^

Figure 2 shows the details pertaining to population segmentation strategies employed. Expert-driven population segmentation was the most common approach utilized (*n* = 111, 75.0%) where all studies employed judgemental splits as the main strategy. With regards to data-driven population segmentation studies, cluster-based segmentation was the main strategy used (*n* = 37, 100%), of which cluster analysis (*n* = 15, 40.5%) and LCA (*n* = 12, 32.4%) were most frequently used.

#### Segmentation variables used

**Fig. 3:**
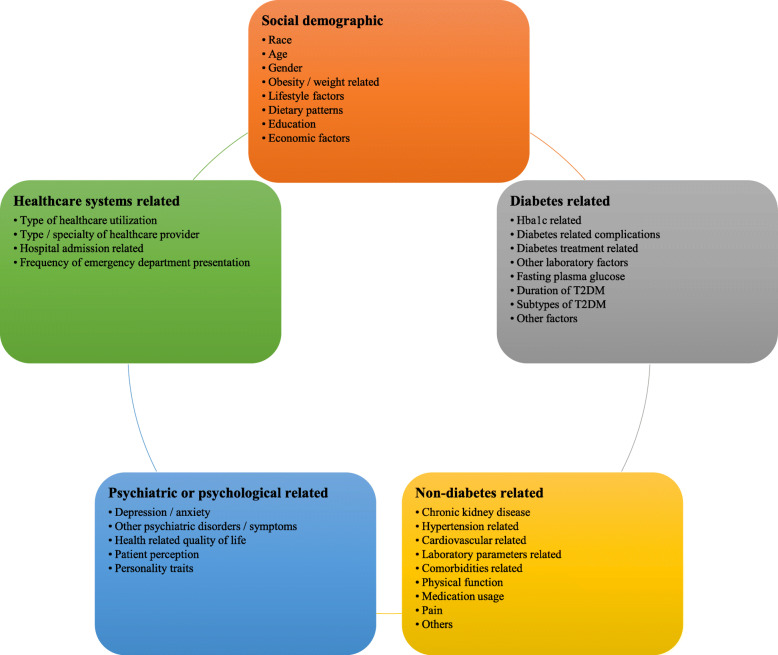
Overview of variables used in population segmentation

Table 2 shows the variables used for population segmentation. Across the five main domains of variables utilized, socio-demographic (*n* = 66, 44.6%), diabetes related (*n* = 54, 36.4%), non-diabetes medical related (*n* = 18, 12.2%) and psychiatric/psychological related variables (*n* = 16, 10.8%) were the most frequently utilized across studies. A total of 85 types of variables from 45 subdomains were utilized as population segmentation variables. A graphical overview of the common subtypes of population segmentation variables used was presented in Fig. 3. Within the domain of socio-demographic related variables, the use of race/ethnicity (*n* = 17, 11.5%) [23, 25, 36–38, 40, 44, 45, 79–86], patient’s age (n = 16, 10.8%) [26, 33, 39, 43, 45, 51, 88–97], gender (n = 12, 8.1%) [41, 56, 67, 72, 79, 92, 94, 97–100] and obesity/weight related (*n* = 7, 4.7%) [17, 49, 58, 59, 102–104] variables were most commonly studied.

**Table 2:** Variables utilized for population segmentation analyses (*N* = 148)

Variables	N^**a**^	Selected examples	References
***Socio-demographic***	**66**		
Race and/or ethnicity	**17**		[23, 25, 36–38, 40, 44, 45, 79–87]
Patients’ age	**16**	Young: ≤ 45 years old, Older: > 45 years old	[26, 33, 39, 43, 51, 87–97]
Gender	**12**		[41, 56, 67, 72, 79, 92, 94, 97–101]
Obesity / weight related	**7**		
- Body mass index / obesity	6		[17, 49, 58, 59, 102, 103]
- Waist circumference	1		[59]
- Weight change over time	1		[104]
Lifestyle factors	**6**		
- Level / patterns of physical activity	4		[20, 32, 57, 105]
- Lifestyle patterns	3	Morningness-eveningness, sleep quality, Consumption of food, alcohol and cigarettes [106]	[20, 106, 107]
Dietary factors / patterns	**6**	e.g. intake of coffee, high fat dietary pattern score	[57, 108–112]
Education related	**4**		
- Level of literacy	2		[48, 107]
- Level of health literacy	1		[18]
- Verbal intelligence	1	Groningen Intelligence Test [113]	[113]
Economic related factors	**4**		
- Household income	2		[48, 114]
- Socio-economic status of patients	1		[115]
- Employment status	1		[107]
Smoking	**4**		
- Current smoking status	3		[20, 34, 57]
- Smoking duration	1		[107]
Alcohol consumption	**1**		[57]
Citizenship (immigrant / non-immigrant)	**1**		[116]
Living in Northern vs Southern latitudes	**1**		[117]
Marital status	**1**		[107]
***Diabetes related***	**54**		
Hba1c related	**14**		
- Hba1c level	7		[74, 76, 103, 118–121]
- Patterns, trends and trajectories	6		[11, 75, 122–125]
- Hba1c variability	2		[125, 126]
Diabetes related complications	**13**		
- Presence of albuminuria	4		[26, 54, 68, 127]
- Presence of complications e.g. cardiometabolic, microvascular diseases	3		[10, 50, 128]
- Presence of diabetic retinopathy	2		[129, 130]
- Severity of albuminuria	1		[54]
- Presence of diabetic nephropathy	1		[130]
- Severity of diabetic neuropathy	1		[131]
- Severity of diabetic kidney disease	1		[132]
- Severity of diabetes related complications	1		[133]
- Medically attended hypoglycemia	1		[30]
Diabetes treatment related	**7**		
- Types of anti-diabetic agents used	5		[10, 26, 69, 134, 135]
- Dose of anti-diabetic agents	1		[136]
- Adherence to anti-diabetic agents	1		[31]
Other laboratory parameters	**5**		
- Presence / levels of islet cells auto-immune antibodies levels	4		[66, 103, 137, 138]
- Glucose-dependent insulinotropic polypepide (GIP) and glucagon like peptide-1 (GLP-1)	1		[70]
Age at T2DM diagnosis	**5**		[60, 78, 93, 103, 139]
Diabetes related risk factors	**5**		
- Number of risk factors	3	Clinical risk groups	[52, 140, 141]
- Total metabolic score	2		[61, 142]
Fasting plasma glucose	**3**		
- Variability of fasting plasma glucose levels	3		[55, 73, 143]
- Mean fasting plasma glucose levels	2		[73, 143]
Duration of T2DM	**4**		[103, 120, 144, 145]
Subtypes of T2DM	**1**	Ketosis prone T2DM or previously diagnosed T2DM with diabetic ketoacidosis [146]	[146]
Status of T2DM at cancer diagnosis	**1**	Insulin resistant /insulin sensitive [24]	[24]
Characteristics of T2DM	**1**	Ketosis	[134]
Severity of diabetes distress	**1**	17-item Diabetes Distress Scale scores [147]	[147]
Presence of metabolic disorder	**1**		[10]
Reasons for not participating in diabetes related progam	**1**		[148]
***Non-diabetes medical related factors***	**18**		
Chronic kidney disease	**6**		
- Severity of chronic kidney disease	4		[26, 65, 68, 132]
- Presence of chronic kidney disease	2		[10, 56]
Hypertension related	**5**		
- Blood pressure control, variability	2		[149, 150]
- Presence of hypertension	1		[102]
- Presence of resistance hypertension	1		[71]
- Number of anti-hypertensive agents	1		[71]
Cardiovascular related	**3**		
- Number of cardiovascular risk factors	1		[50]
- Presence of atherosclerosis	1		[51]
- Echocardiographic variables	1		[102]
Physical function status related	**2**		
- Physical functional status	1		[151]
- Frailty	1		[152]
Laboratory related parameters	**2**		
- Blood biochemistries / full blood count	1		[26]
- Low-density lipoprotein (LDL) levels	1		[63]
Comorbidities of patients (overall)	**1**		[128]
Overall medication usage	**1**		[26]
Severity of pain	**1**		[131]
Microaneurysm turnover and for the central retinal thickness	**1**		[153]
***Psychiatric or psychological related factors***	**16**		
Depression or anxiety related	**9**		
- Presence and severity of depression and anxiety symptoms	3		[42, 154, 155]
- Severity of depression	3		[21, 35, 42]
- Presence of depression	2		[27, 156]
- Patterns of depression	1	Persistant depressive symptoms, new depressive symptoms, remitted depressive symptoms, no or few depressive symptoms [29]	[29]
- Severity of anxiety symptoms	1		[46]
Other psychiatric disorders / symptoms related factors	**5**		
- Presence of sleep disturbance, fatigue	2		[42, 131]
- Number of psychiatric conditions	1		[22]
- Severity of psychiatric symptoms	1	Brief Psychiatric Rating Scale score	[35]
- Cognitive status	1		[62]
Health related quality of life	**3**		[118, 131, 157]
Patient perceptions related factors	**2**		
- Illness perception	1		[158]
- Perceived self-efficacy	1		[35]
- Perceived social support	1		[35]
Personality traits differences	**1**		[159]
***Healthcare systems related***	**6**		
Type of healthcare utilization	2		[160, 161]
Type or specialty of care provider	2		[64, 77]
Hospital admission	1		[53]
Frequency of emergency department presentation	1		[162]

*Abbreviations*: *N* Number of studies

^a^The number of unique studies were reported for the subtotals within and for each domain (in bold) to avoid double counting of studies

Within the domain of diabetes related variables, Hba1c related (*n* = 14, 9.5%) [11, 67, 74–76, 103, 118–124, 126], diabetes related complications (*n* = 13, 8.8%) [10, 26, 30, 50, 54, 57, 68, 127, 129–133] and diabetes treatment related (n = 7, 4.7%) [10, 26, 31, 69, 134–136] variables were the most commonly utilized variables for population segmentation.

With regards to the domain of non-diabetes medical related variables, chronic kidney disease (*n* = 6, 4.1%) [10, 26, 56, 65, 68, 132], hypertension (*n* = 5, 3.4%) [71, 102, 149, 150], and cardiovascular disease related (*n* = 3, 2.0%) [50, 51, 102]. Pertaining to the domain of psychiatric / psychological related variables, depression/anxiety related (*n* = 9, 6.1%) [21, 27, 29, 35, 42, 46, 154–156] and other psychiatric disorders/symptoms related (n = 5, 3.4%) [22, 35, 42, 62, 131] variables were most commonly employed. Lastly for the health systems related domain, types of healthcare utilization (*n* = 2, 1.4%) [160, 161] and type/specialty of care providers (n = 2, 1.4%) [64, 77] related variables were most frequently used.

#### Objectives of population segmentation strategies and number of derived segments

**Fig. 4:**
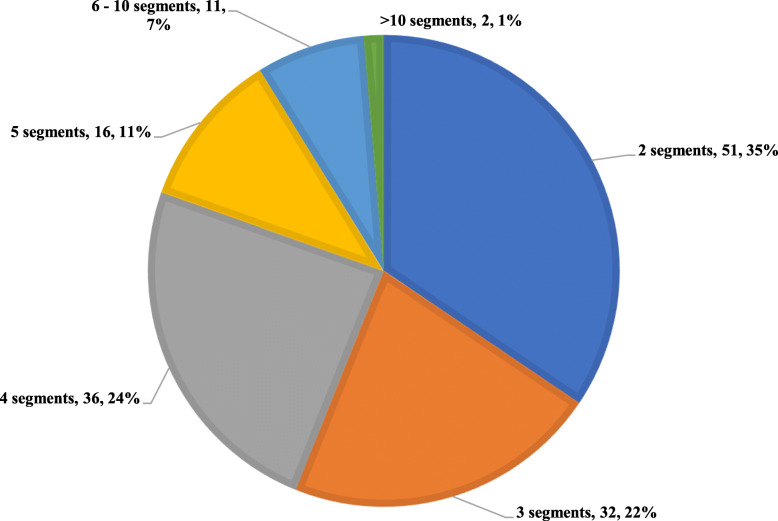
Number of patient segments derived within included studies

Health grouping/profiling (*n* = 71, 48%), assessment of differential risk of diabetes related complications (*n* = 57, 38.5%), non-diabetes metabolic derangements (e.g. lipids and blood pressure) (*n* = 42, 28.4%) and diabetes control (*n* = 40, 27.0%) were the most frequent population segmentation objectives of the studies. (Table 3) The number of patient segments derived ranged from one to ten segments, of which two to four segments (*n* = 119, 80.4%) were most commonly derived number of segments. (Fig. 4).

**Table 3:** Objectives of population segmentation

Characteristics of studies	N (%)
***Objective of segmentation***	
Health grouping or profiling	71 (48.0)
Assess risk of diabetic related complications across groups	57 (38.5)
Assess non-diabetes metabolic derangements (e.g. lipid, blood pressure) across groups	42 (28.4)
Assess diabetic control across groups	40 (27.0)
Assess healthcare utilization	17 (11.5)
Assess mortality	16 (10.8)
Assess treatment outcomes	10 (6.8)
Assess quality of life across groups	4 (2.7)
Assess psychological symptoms across groups	3 (2.0)
Assess risk of psychological outcomes across groups	3 (2.0)
Asses treatment adherence	3 (2.0)
Assess accessibility to providers and healthcare services	1 (0.7)
Assess cognitive related outcomes across groups	1 (0.7)
Assess physical function across groups	1 (0.7)
Assess obesity rates across groups	1 (0.7)
Assess pregnancy related outcomes	1 (0.7)

#### PASS-T2DM framework

**Fig. 5:**
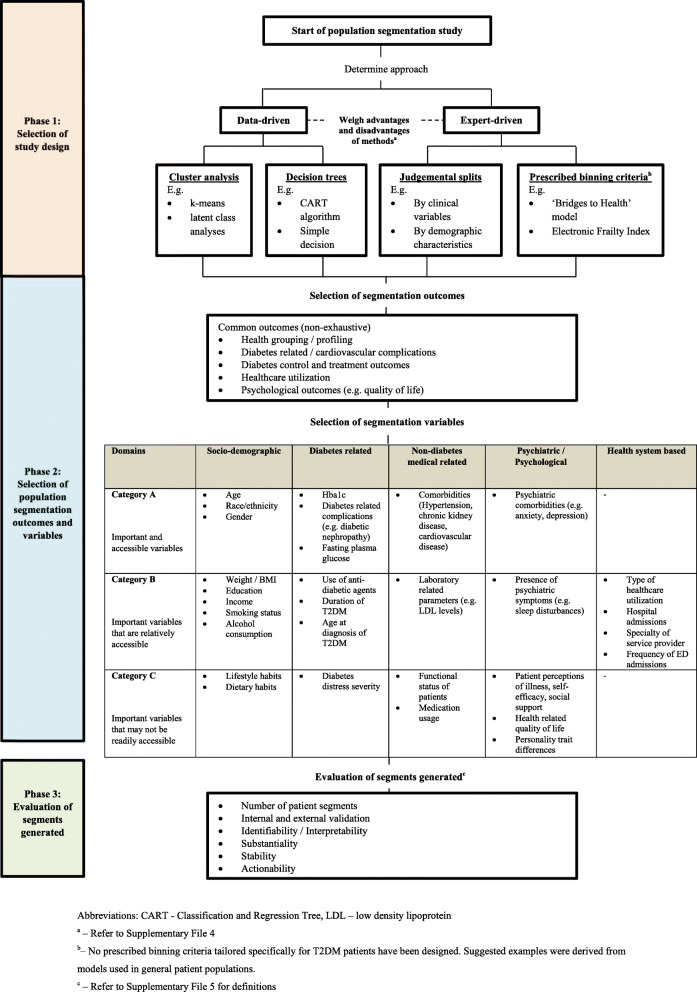
Population Segmentation Studies design framework for T2DM patients (PASS-T2DM)

Figure 5 shows the proposed PASS-T2DM framework, which comprises of three phases: 1) Selection of study design; 2) Selection of population segmentation outcomes and variables and 3) Evaluation of segments generated. For Phase 2, there is generally no preferred order for the selection of population segmentation outcomes and variables. One exception lies in the use of CART which requires the segmentation outcome to be determined beforehand. For other methodologies, concurrent selection of segmentation variables and outcomes may be performed at the user’s discretion. Examples of commonly utilized segmentation outcomes for consideration include health profiling of patients or assessment of patients’ differential risk of T2DM related complications. For segmentation variables that were classified as “Category A: important and accessible variables”, these included patient’s age, gender, race / ethnicity, Hba1c levels, diabetes related complications, presence of non-psychiatric and psychiatric comorbidities. These variables were selected after careful evaluation of their clinical importance and relative accessibility. Additionally, they were among the most commonly used segmentation variables across studies.

## Discussion

### Summary of evidence

Overall, this scoping review has summarized the clinical applications of population segmentation strategies among T2DM patients. To our best knowledge, this is also the first review which evaluated the clinical applications of population strategies for T2DM patients and proposed a framework for the design of population segmentation studies for T2DM patients.

As shown in the review, a multitude of population segmentation strategies encompassing both data-driven and expert-riven population segmentation approaches have been utilized among T2DM patients. Importantly, each methodology carries its inherent advantages and disadvantages [10, 14, 16, 163]. The main merit of judgemental splitting, which is the most studied expert-driven methodology is its simplicity of use, where a patient population is divided into segments based on one or more explanatory variables [14]. Conversely, one of its disadvantages is non-objectivity, where the discriminatory properties of the target variable have not been actively sought [14]. Furthermore, the use of certain population segmentation variables may lead to excessive number of segments, which may have inadequate discriminatory properties [14]. For cluster based analysis which was the main data-driven population segmentation analyses employed, its chief advantage lies in its ability to manage multiple types of population segmentation variables, which can be continuous or categorical in nature [163, 164]. However, certain cluster based analyses techniques such as hierarchical analyses and k-means cluster analyses are affected by outliers [163]. In this review, there were no studies which have utilized decision trees analyses or prescribed binning criteria for the segmentation of T2DM patients. Future studies should consider the use of these strategies to evaluate their potential role in segmentation of T2DM patients. Currently, the optimal population segmentation methodology for T2DM patients has not been established. As such, researchers should be cognizant of the advantages and disadvantages of each population segmentation methodology when selecting an appropriate technique for their studies. Additional factors that should be considered during the selection process include the type of population segmentation variables to be used, the properties of the dataset, research questions and level of technical and statistical expertise of the researchers [165].

With regards to population segmentation variables used in T2DM studies, 85 sub-groups of variables were identified in our review. Given the wide array of population segmentation variables available, potential computation challenge exists when processing large number of segmentation variables and observations during the implementation of population segmentation strategies. Hence, careful selection and screening of variables needs to be performed to achieve a balance between number of patient segments derived and sufficient discriminatory properties from the derived segments. In the PASS-T2DM framework, variables which included patient’s age, gender, race / ethnicity, Hba1c levels, diabetes related complications, presence of non-psychiatric and psychiatric comorbidities e.g. hypertension, chronic kidney disease, anxiety and depression were classified as Category A variables which correspond to variables that are of high clinical importance and relative accessibility. Of note, these variables were also the most frequently utilized segmentation variables across included studies for the review.

Within the domain of socio-demographic variables, age is a well-recognized driver of the global rise in diabetes [166] and T2DM among older adults have been associated with increased mortality, poorer functional status and risk of hospitalisation [167]. On the other end of the spectrum, there is also a growing epidemic of early-onset T2DM among young adults, which has been attributed to complex interplay of lifestyle and genetic factors such as sedentary lifestyles and obesity [168]. For example, the SEARCH study showed that the incidence of T2DM among young people increased by 7.1% between 2002 and 2003 and 2011–2012 in the United States [169]. Unsurprisingly, studies which have employed age as a population segmentation variable were able to generate patient segments with differential risk of diabetes related complications, diabetes control and cardiovascular risk profiles [51, 88, 89]. With regards to the role of gender, sexual dimorphisms related to pathophysiological mechanisms of T2DM and its complications have been gaining interest in the recent years [170]. Gender differences in the clinical presentation of T2DM and risk of diabetes related complications have been postulated to involve a multitude of biological, cultural, lifestyle, environmental and socio-economic factors [170]. For example, a study by Logue et al. showed that diabetic men tend to be diagnosed at an earlier age and lower body mass index as compared to women [171]. Conversely, diabetic women tended to have higher risk of stroke related mortality when compared to their male counterparts [172]. For ethnicity, it has been implicated in the development of T2DM related lower extremity amputations and microvascular complications, where higher rates of these complications have been reported among minority ethnic groups [173]. The use of gender and ethnicity as segmentation variables have similarly generated distinctive patient segments with varying risk of diabetes related outcomes [25, 67, 80, 98].

With regards to diabetes related variables, Hba1c is a well-established measure of glycaemic control and has been shown in the Diabetes Control and Complications Trial (DCCT) to be quintessential for prevention of diabetes related complications [174]. In studies which segmented patients based on Hba1c levels, patients with poorer glycaemic control were consistently shown to have increased risk of diabetes related complications [76, 118]. A variant of this measure, Hba1c variability and its relationship with diabetic complications has been increasingly studied although there have been conflicting results [175]. While a recent study showed a positive correlation between increased Hba1c variability and all-cause mortality [176], a post-hoc analysis from the DCCT trial showed no association between glycaemic variability and developing adverse clinical outcomes [177]. Consequently, researchers who are designing population segmentation studies should consider the use of Hba1c variability for exploratory purposes until more evidence supporting its use emerges. For diabetes related complications, their association with morbidity and mortality, as well as their resultant impact on healthcare resource consumption and economic burden are well-established and recognized [9]. In studies where patients were segmented by the presence or severity of diabetes related complications, distinct patient segments were derived with differing healthcare utilization, mortality and morbidity [9, 133].

Pertaining to non-diabetes medical related and psychiatric/psychological related domains, the presence of co-morbidities such as hypertension, chronic kidney disease, depression and anxiety are common and often result in additional financial and psychological burden on patients [178]. Notably, these comorbidities may have profound impacts on the self-care ability of patients. For example, depression often results in significant impairment of patients’ functioning and may present as barriers to adherence to lifestyle modifications and treatment regimens [179]. Managing the healthcare needs of T2DM patients with varying types of multi-morbidities is challenging and there is a need for changes in the health system to meet the needs of these patients. Population segmentation is a valuable endeavour which can segregate T2DM patients into more manageable patient segments, to facilitate the design of targeted interventions.

As seen from this review, population segmentation has a wide range of clinical applications, ranging from health group profiling to assessing the differential risk of diabetes related complications and mortality. This highlights the versatility of population segmentation and its applications. With the rising use of electronic health records in big data analytics, future population segmentation studies may wish to leverage on the recent advancement in big data by streamlining and tailoring their study designs to population segmentation variables and outcomes which are readily available in the electronic health records or can be easily incorporated into electronic health records at routine clinical care touch points between patients and healthcare providers to reduce the burden placed onto healthcare professionals [5].

In this review, we identified 148 studies which have utilized data-driven and expert-driven population segmentation strategies to identify subgroups of T2DM patients with differential health related outcomes or healthcare utilization patterns. The main strength of this review was that the proposed PASS-T2DM framework provides a simple overview for future researchers to design population segmentation studies for T2DM patients.

### Limitations

However, users of this framework should also be cognizant of its potential limitations. The segmentation variables included within the framework were restricted to those evaluated across included studies and should not be regarded as an exhaustive list. Researchers planning to utilize variables outside the list should evaluate these variables carefully prior to their inclusion. In addition, this highlights the need for more studies to explore the role of other potentially useful population segmentation variables not listed in the framework such as medication compliance rates. Furthermore, while the proposed framework in our study had categorized population segmentation variables on the basis of their relative clinical importance and accessibility, the optimal set and combination of population segmentation variables which is context specific for the different aims of population segmentation remains unclear. Nonetheless, our review serves as an important foundation for future researchers to evaluate and determine the optimal set of population segmentation variables that should be used. With regards to other limitations related to this review, the grey literature was not searched, which could have led to omission of potentially relevant articles. Future reviews which plan to update this topic should consider searching grey literature. Another limitation was that non-English articles as well as studies which included children or adolescents with T2DM were excluded. Lastly, a formal assessment of the methodological limitations of the evidence was not performed as it was not the objective of this study. Nonetheless, researchers conducting future systematic reviews to evaluate specific population segmentation methodologies should evaluate the risk of bias of included studies [180]. This will aid in identifying the optimal combination of population segmentation variables to be used for each methodology.

## Conclusion

Population segmentation methodologies via data-driven or expert-driven approaches are important tools that can aid policymakers and healthcare administrators in evaluating a wide range of outcomes among different sub-groups of T2DM patients, ranging from health profiling to assessing the differential risk of diabetes related complications. While a large number of population segmentation variables have been used in literature, the optimal combination of population segmentation variables to be used remains unknown and should be explored in future studies. The proposed PASS-T2DM framework for the design of population segmentation studies will serve as an important guide for researchers to structure and design population segmentation studies for T2DM patients until the optimal framework has been established. More studies are required to explore the role of population segmentation variables not listed in the framework.

## Supplementary Information

**Additional file 1.** Details of full search strategy

**Additional file 2.** Details of included studies

**Additional file 3 **Funding sources for included studies (*n* = 148)

**Additional file 4.** Advantages and disadvantages of population segmentation methods [14, 181]

**Additional file 5.** Definitions of criterion used for evaluation of population segmentation outcomes [16, 181]

## Data Availability

All data generated or analyzed during this study are included in this published article and its supplementary information files.

## Abbreviations

CART: Classification and regression tree
LCA: Latent class analyses
PASS-T2DM: PopulAtion Segmentation Studies design framework for T2DM patients
T2DM: Type 2 diabetes mellitus
