# Supplementary material for: Population segmentation of type 2 diabetes mellitus patients and its clinical applications - a scoping review
Source: BMC Med Res Methodol. 2021 Mar 11;21:49. doi: 10.1186/s12874-021-01209-w (PMC7953703; doi:10.1186/s12874-021-01209-w)
Supplement: Supplementary file 1 — Additional file 1. Details of full search strategy [file 12874_2021_1209_MOESM1_ESM.docx]

**Supplementary File 1. Details of full search strategy**

**1) Medline®**

Search strategy : #1 AND 2

| **S/No** | **Keywords** | **Terms** |
| --- | --- | --- |
| **1** | **Type 2 Diabetes Mellitus** | ("Diabetes Mellitus"[Mesh] OR "Diabetes Mellitus, Type 2"[mh] OR "diabetes mellitus"[Title/Abstract] OR t2dm[Title/Abstract] OR "type 2 diabetes mellitus"[Title/Abstract] OR NIDDM[Title/Abstract]) |
| **2** | **Population Segmentation** | ("cluster analysis"[MeSH] OR (cluster[Title/Abstract] OR cluster'[Title/Abstract] OR cluster''[Title/Abstract] OR cluster's[Title/Abstract] OR cluster1[Title/Abstract] OR cluster2[Title/Abstract] OR cluster23[Title/Abstract] OR cluster3[Title/Abstract] OR cluster32[Title/Abstract] OR cluster4[Title/Abstract] OR cluster5[Title/Abstract] OR cluster50[Title/Abstract] OR cluster58[Title/Abstract] OR cluster6[Title/Abstract] OR cluster7[Title/Abstract] OR cluster70[Title/Abstract] OR cluster8[Title/Abstract] OR cluster90[Title/Abstract] OR cluster94[Title/Abstract] OR clustera[Title/Abstract] OR clusterability[Title/Abstract] OR clusterable[Title/Abstract] OR clusteral[Title/Abstract] OR clusterally[Title/Abstract] OR clusteranalyses[Title/Abstract] OR clusteranalysis[Title/Abstract] OR clusteranalytic[Title/Abstract] OR clusteranalytically[Title/Abstract] OR clusteranion[Title/Abstract] OR clusteranions[Title/Abstract] OR clusterb[Title/Abstract] OR clusterbase[Title/Abstract] OR clusterbases[Title/Abstract] OR clusterbean[Title/Abstract] OR clusterbfs[Title/Abstract] OR clusterblast[Title/Abstract] OR clusterbootstrap[Title/Abstract] OR clusterbud[Title/Abstract] OR clusterbuster[Title/Abstract] OR clusterbusters[Title/Abstract] OR clusterc[Title/Abstract] OR clustercad[Title/Abstract] OR clustercall[Title/Abstract] OR clustercons[Title/Abstract] OR clustercontaining[Title/Abstract] OR clustercontrol[Title/Abstract] OR clusterd[Title/Abstract] OR clusterdelta[Title/Abstract] OR clusterdeltaptlh[Title/Abstract] OR clusterdp[Title/Abstract] OR clusterdraw[Title/Abstract] OR clusterdv[Title/Abstract] OR clustere[Title/Abstract] OR clustere6[Title/Abstract] OR clusterectomy[Title/Abstract] OR clustered[Title/Abstract] OR clustered'[Title/Abstract] OR clustered''[Title/Abstract] OR clustereddot[Title/Abstract] OR clusteredephrina5fc[Title/Abstract] OR clusteredin[Title/Abstract] OR clusteredness[Title/Abstract] OR clusterelectric[Title/Abstract] OR clustereng[Title/Abstract] OR clusterer[Title/Abstract] OR clustererd[Title/Abstract] OR clusterered[Title/Abstract] OR clusterers[Title/Abstract] OR clusteres[Title/Abstract] OR clusterex[Title/Abstract] OR clusterexperiment[Title/Abstract] OR clusterfast[Title/Abstract] OR clusterfinder[Title/Abstract] OR clusterfinder'[Title/Abstract] OR clusterflock[Title/Abstract] OR clusterflow[Title/Abstract] OR clusterforming[Title/Abstract] OR clusterfullerene[Title/Abstract] OR clusterfullerenes[Title/Abstract] OR clustergenedb[Title/Abstract] OR clustergenedb'[Title/Abstract] OR clustergenomics[Title/Abstract] OR clustergram[Title/Abstract] OR clustergrammer[Title/Abstract] OR clustergrams[Title/Abstract] OR clustergrouped[Title/Abstract] OR clusterhead[Title/Abstract] OR clusterheads[Title/Abstract] OR clusterid[Title/Abstract] OR clusterification[Title/Abstract] OR clusterig[Title/Abstract] OR clusterin[Title/Abstract] OR clusterin'[Title/Abstract] OR clusterin's[Title/Abstract] OR clusterinassociated[Title/Abstract] OR clusterine[Title/Abstract] OR clustering[Title/Abstract] OR clustering'[Title/Abstract] OR clustering''[Title/Abstract] OR clustering's[Title/Abstract] OR clusteringbased[Title/Abstract] OR clusteringin[Title/Abstract] OR clusteringly[Title/Abstract] OR clusteringmethod[Title/Abstract] OR clusteringof[Title/Abstract] OR clusterings[Title/Abstract] OR clusterins[Title/Abstract] OR clusterisation[Title/Abstract] OR clusterised[Title/Abstract] OR clusteriv[Title/Abstract] OR clusterization[Title/Abstract] OR clusterizations[Title/Abstract] OR clusterize[Title/Abstract] OR clusterized[Title/Abstract] OR clusterizer[Title/Abstract] OR clusterizes[Title/Abstract] OR clusterlab[Title/Abstract] OR clusterless[Title/Abstract] OR clusterlike[Title/Abstract] OR clusterlocator[Title/Abstract] OR clusterlustre[Title/Abstract] OR clusterly[Title/Abstract] OR clusterm[Title/Abstract] OR clustermaker[Title/Abstract] OR clustermap[Title/Abstract] OR clustermatch[Title/Abstract] OR clustermates[Title/Abstract] OR clustermerge[Title/Abstract] OR clustermhcii[Title/Abstract] OR clustermi[Title/Abstract] OR clustermine360[Title/Abstract] OR clustermq[Title/Abstract] OR clusternanalyse[Title/Abstract] OR clusterness[Title/Abstract] OR clusterness'[Title/Abstract] OR clusternetwork[Title/Abstract] OR clusterngo[Title/Abstract] OR clusternomics[Title/Abstract] OR clusterof[Title/Abstract] OR clusterograms[Title/Abstract] OR clusteroids[Title/Abstract] OR clusteroluminescence[Title/Abstract] OR clusteroluminogens[Title/Abstract] OR clusteron[Title/Abstract] OR clusterone[Title/Abstract] OR clusterons[Title/Abstract] OR clusterophobia[Title/Abstract] OR clusterpicker[Title/Abstract] OR clusterpickergui[Title/Abstract] OR clusterpost[Title/Abstract] OR clusterpower[Title/Abstract] OR clusterpro[Title/Abstract] OR clusterprofile[Title/Abstract] OR clusterprofiler[Title/Abstract] OR clusterprofilers[Title/Abstract] OR clusterprominence[Title/Abstract] OR clusterpso[Title/Abstract] OR clusterr[Title/Abstract] OR clusterrandomised[Title/Abstract] OR clusterrandomisedtrial[Title/Abstract] OR clusterrandomized[Title/Abstract] OR clusterrank[Title/Abstract] OR clusterreg[Title/Abstract] OR clusterrendomized[Title/Abstract] OR clusterrepro[Title/Abstract] OR clusters[Title/Abstract] OR clusters'[Title/Abstract] OR clusters''[Title/Abstract] OR clustersampling[Title/Abstract] OR clusterscan[Title/Abstract] OR clusterscansupport[Title/Abstract] OR clusterscomprised[Title/Abstract] OR clusterscore[Title/Abstract] OR clusterscout[Title/Abstract] OR clusterseer[Title/Abstract] OR clustershade[Title/Abstract] OR clustershave[Title/Abstract] OR clustersignificance[Title/Abstract] OR clustersim[Title/Abstract] OR clustersindicating[Title/Abstract] OR clustersing[Title/Abstract] OR clustersize[Title/Abstract] OR clustersmark[Title/Abstract] OR clustersomes[Title/Abstract] OR clustersomes'[Title/Abstract] OR clustersour[Title/Abstract] OR clusterspots[Title/Abstract] OR clusterstructures[Title/Abstract] OR clustersvdd[Title/Abstract] OR clustertad[Title/Abstract] OR clustertlbo[Title/Abstract] OR clustertrays[Title/Abstract] OR clustertree[Title/Abstract] OR clusterv[Title/Abstract] OR clustervalidation[Title/Abstract] OR clustervine[Title/Abstract] OR clustervision[Title/Abstract] OR clustervisu[Title/Abstract] OR clusterviz[Title/Abstract] OR clustervize[Title/Abstract] OR clusterw[Title/Abstract] OR clusterwhen[Title/Abstract] OR clusterwide[Title/Abstract] OR clusterwise[Title/Abstract] OR clusterx20[Title/Abstract] OR clusterxtime[Title/Abstract] OR clustery[Title/Abstract] OR clusterz[Title/Abstract]) OR (typologia[Title/Abstract] OR typologic[Title/Abstract] OR typological[Title/Abstract] OR typologically[Title/Abstract] OR typologie[Title/Abstract] OR typologies[Title/Abstract] OR typologies'[Title/Abstract] OR typologisation[Title/Abstract] OR typologische[Title/Abstract] OR typologischem[Title/Abstract] OR typologise[Title/Abstract] OR typologised[Title/Abstract] OR typologism[Title/Abstract] OR typologists[Title/Abstract] OR typologization[Title/Abstract] OR typologize[Title/Abstract] OR typologized[Title/Abstract] OR typologizing[Title/Abstract] OR typology[Title/Abstract] OR typology'[Title/Abstract] OR typology's[Title/Abstract] OR typologyprovided[Title/Abstract]) OR (stratifactory[Title/Abstract] OR stratifcation[Title/Abstract] OR stratifed[Title/Abstract] OR stratifera[Title/Abstract] OR stratiffed[Title/Abstract] OR stratifi[Title/Abstract] OR stratifiable[Title/Abstract] OR stratifica[Title/Abstract] OR stratificaion[Title/Abstract] OR stratifical[Title/Abstract] OR stratificantion[Title/Abstract] OR stratificatation[Title/Abstract] OR stratificate[Title/Abstract] OR stratificated[Title/Abstract] OR stratificating[Title/Abstract] OR stratificatio[Title/Abstract] OR stratification[Title/Abstract] OR stratification'[Title/Abstract] OR stratification's[Title/Abstract] OR stratificational[Title/Abstract] OR stratificationed[Title/Abstract] OR stratifications[Title/Abstract] OR stratificationtool[Title/Abstract] OR stratificationwas[Title/Abstract] OR stratificative[Title/Abstract] OR stratificaton[Title/Abstract] OR stratificator[Title/Abstract] OR stratificators[Title/Abstract] OR stratificazione[Title/Abstract] OR stratificiation[Title/Abstract] OR stratifide[Title/Abstract] OR stratifieation[Title/Abstract] OR stratified[Title/Abstract] OR stratified'[Title/Abstract] OR stratifiedaccording[Title/Abstract] OR stratifiedcox[Title/Abstract] OR stratifiedly[Title/Abstract] OR stratifiee[Title/Abstract] OR stratifield[Title/Abstract] OR stratifier[Title/Abstract] OR stratifiers[Title/Abstract] OR stratifies[Title/Abstract] OR stratifification[Title/Abstract] OR stratifikacio[Title/Abstract] OR stratifin[Title/Abstract] OR stratifing[Title/Abstract] OR stratifiying[Title/Abstract] OR stratifizierte[Title/Abstract] OR stratifizierter[Title/Abstract] OR stratifizierung[Title/Abstract] OR stratiform[Title/Abstract] OR stratiformity[Title/Abstract] OR stratify[Title/Abstract] OR stratify'[Title/Abstract] OR stratifyed[Title/Abstract] OR stratifying[Title/Abstract] OR stratifyjcv[Title/Abstract]) OR (segment[Title/Abstract] OR segment'[Title/Abstract] OR segment''[Title/Abstract] OR segment's[Title/Abstract] OR segment1[Title/Abstract] OR segment2[Title/Abstract] OR segment9[Title/Abstract] OR segmenta[Title/Abstract] OR segmentability[Title/Abstract] OR segmentable[Title/Abstract] OR segmentacao[Title/Abstract] OR segmentacion[Title/Abstract] OR segmentacoes[Title/Abstract] OR segmentado[Title/Abstract] OR segmentai[Title/Abstract] OR segmentaion[Title/Abstract] OR segmentaire[Title/Abstract] OR segmentaires[Title/Abstract] OR segmental[Title/Abstract] OR segmental'[Title/Abstract] OR segmentale[Title/Abstract] OR segmentalen[Title/Abstract] OR segmentaler[Title/Abstract] OR segmentales[Title/Abstract] OR segmentalfractures[Title/Abstract] OR segmentalinic[Title/Abstract] OR segmentalis[Title/Abstract] OR segmentalised[Title/Abstract] OR segmentalism[Title/Abstract] OR segmentalist[Title/Abstract] OR segmentalists[Title/Abstract] OR segmentality[Title/Abstract] OR segmentalization[Title/Abstract] OR segmentalizations[Title/Abstract] OR segmentalize[Title/Abstract] OR segmentalized[Title/Abstract] OR segmentalizing[Title/Abstract] OR segmentallordosis[Title/Abstract] OR segmentally[Title/Abstract] OR segmentals[Title/Abstract] OR segmentalsize[Title/Abstract] OR segmentaly[Title/Abstract] OR segmentanalyzer[Title/Abstract] OR segmentar[Title/Abstract] OR segmentaria[Title/Abstract] OR segmentarian[Title/Abstract] OR segmentaries[Title/Abstract] OR segmentarily[Title/Abstract] OR segmentario[Title/Abstract] OR segmentary[Title/Abstract] OR segmentata[Title/Abstract] OR segmentate[Title/Abstract] OR segmentated[Title/Abstract] OR segmentatin[Title/Abstract] OR segmentating[Title/Abstract] OR segmentatio[Title/Abstract] OR segmentation[Title/Abstract] OR segmentation'[Title/Abstract] OR segmentation's[Title/Abstract] OR segmentationa[Title/Abstract] OR segmentational[Title/Abstract] OR segmentationm[Title/Abstract] OR segmentationnets[Title/Abstract] OR segmentations[Title/Abstract] OR segmentations'[Title/Abstract] OR segmentative[Title/Abstract] OR segmentatus[Title/Abstract] OR segmentbmca[Title/Abstract] OR segmentctomy[Title/Abstract] OR segmente[Title/Abstract] OR segmentectomia[Title/Abstract] OR segmentectomias[Title/Abstract] OR segmentectomie[Title/Abstract] OR segmentectomies[Title/Abstract] OR segmentectomiess[Title/Abstract] OR segmentectomiy[Title/Abstract] OR segmentectomy[Title/Abstract] OR segmentectomy'[Title/Abstract] OR segmentectomym[Title/Abstract] OR segmentectomywith[Title/Abstract] OR segmented[Title/Abstract] OR segmented'[Title/Abstract] OR segmentee[Title/Abstract] OR segmentel[Title/Abstract] OR segmentelevation[Title/Abstract] OR segmentenglish[Title/Abstract] OR segmenter[Title/Abstract] OR segmenters[Title/Abstract] OR segmentes[Title/Abstract] OR segmentestomies[Title/Abstract] OR segmentetomy[Title/Abstract] OR segmential[Title/Abstract] OR segmentically[Title/Abstract] OR segmentierung[Title/Abstract] OR segmentifida[Title/Abstract] OR segmentina[Title/Abstract] OR segmenting[Title/Abstract] OR segmentingly[Title/Abstract] OR segmentini[Title/Abstract] OR segmentinin[Title/Abstract] OR segmentinini[Title/Abstract] OR segmention[Title/Abstract] OR segmentism[Title/Abstract] OR segmentitis[Title/Abstract] OR segmentize[Title/Abstract] OR segmentized[Title/Abstract] OR segmentmovement[Title/Abstract] OR segmentmrs[Title/Abstract] OR segmentnon[Title/Abstract] OR segmento[Title/Abstract] OR segmentogranular[Title/Abstract] OR segmentography[Title/Abstract] OR segmentomy[Title/Abstract] OR segmentonuclear[Title/Abstract] OR segmentonucleary[Title/Abstract] OR segmentootion[Title/Abstract] OR segmentor[Title/Abstract] OR segmentor3isback[Title/Abstract] OR segmentorbis[Title/Abstract] OR segmentorientated[Title/Abstract] OR segmentors[Title/Abstract] OR segmentory[Title/Abstract] OR segmentos[Title/Abstract] OR segmentosa[Title/Abstract] OR segmentosum[Title/Abstract] OR segmentotherapy[Title/Abstract] OR segmentotomie[Title/Abstract] OR segmentotomy[Title/Abstract] OR segmentotopic[Title/Abstract] OR segmentous[Title/Abstract] OR segmentperfusion[Title/Abstract] OR segmentplasty[Title/Abstract] OR segmentral[Title/Abstract] OR segments[Title/Abstract] OR segments'[Title/Abstract] OR segmentsand[Title/Abstract] OR segmentsare[Title/Abstract] OR segmentseq[Title/Abstract] OR segmentsmthe[Title/Abstract] OR segmentsof[Title/Abstract] OR segmentspecific[Title/Abstract] OR segmentsrelative[Title/Abstract] OR segmentum[Title/Abstract] OR segmentun[Title/Abstract] OR segmentv[Title/Abstract] OR segmentwas[Title/Abstract] OR segmentwise[Title/Abstract] OR segmentxage[Title/Abstract] OR segmentxcondition[Title/Abstract] OR segmentxposition[Title/Abstract]) OR (class[Title/Abstract] OR class'[Title/Abstract] OR class''[Title/Abstract] OR class'es[Title/Abstract] OR class's[Title/Abstract] OR class02[Title/Abstract] OR class1[Title/Abstract] OR class10[Title/Abstract] OR class11[Title/Abstract] OR class14[Title/Abstract] OR class1and[Title/Abstract] OR class1and2[Title/Abstract] OR class1multidomain[Title/Abstract] OR class1sirtuin[Title/Abstract] OR class2[Title/Abstract] OR class2000[Title/Abstract] OR class3[Title/Abstract] OR class4[Title/Abstract] OR class5[Title/Abstract] OR class8[Title/Abstract] OR classa[Title/Abstract] OR classable[Title/Abstract] OR classafied[Title/Abstract] OR classamp[Title/Abstract] OR classand[Title/Abstract] OR classant[Title/Abstract] OR classantes[Title/Abstract] OR classat[Title/Abstract] OR classb[Title/Abstract] OR classbase[Title/Abstract] OR classbases[Title/Abstract] OR classcal[Title/Abstract] OR classcanine[Title/Abstract] OR classcation[Title/Abstract] OR classchain[Title/Abstract] OR classchildren[Title/Abstract] OR classcification[Title/Abstract] OR classcloud[Title/Abstract] OR classcraniofacial[Title/Abstract] OR classd1[Title/Abstract] OR classdb[Title/Abstract] OR classdependent[Title/Abstract] OR classdis[Title/Abstract] OR classdiscovery[Title/Abstract] OR classe[Title/Abstract] OR classecond[Title/Abstract] OR classed[Title/Abstract] OR classee[Title/Abstract] OR classees[Title/Abstract] OR classement[Title/Abstract] OR classemes[Title/Abstract] OR classen[Title/Abstract] OR classer[Title/Abstract] OR classes[Title/Abstract] OR classes'[Title/Abstract] OR classesa[Title/Abstract] OR classesas[Title/Abstract] OR classeshs[Title/Abstract] OR classesii[Title/Abstract] OR classesin[Title/Abstract] OR classess[Title/Abstract] OR classeyana[Title/Abstract] OR classfication[Title/Abstract] OR classfications[Title/Abstract] OR classficatory[Title/Abstract] OR classfied[Title/Abstract] OR classfiers[Title/Abstract] OR classflied[Title/Abstract] OR classfying[Title/Abstract] OR classgene[Title/Abstract] OR classgenomic[Title/Abstract] OR classgsts[Title/Abstract] OR classhla[Title/Abstract] OR classhood[Title/Abstract] OR classhours[Title/Abstract] OR classi[Title/Abstract] OR classia[Title/Abstract] OR classial[Title/Abstract] OR classiand[Title/Abstract] OR classiantigens[Title/Abstract] OR classic[Title/Abstract] OR classic'[Title/Abstract] OR classic''[Title/Abstract] OR classic'plasmodium[Title/Abstract] OR classic's[Title/Abstract] OR classic'strade[Title/Abstract] OR classica[Title/Abstract] OR classicabpa[Title/Abstract] OR classical[Title/Abstract] OR classical'[Title/Abstract] OR classical''[Title/Abstract] OR classical'actinomyces[Title/Abstract] OR classical'method[Title/Abstract] OR classical'nitrosomonas[Title/Abstract] OR classical`als[Title/Abstract] OR classical`antiepileptic[Title/Abstract] OR classicalbzds[Title/Abstract] OR classicalcutoff[Title/Abstract] OR classicalgt[Title/Abstract] OR classicalities[Title/Abstract] OR classicality[Title/Abstract] OR classicalization[Title/Abstract] OR classicalizing[Title/Abstract] OR classicallike[Title/Abstract] OR classically[Title/Abstract] OR classically'[Title/Abstract] OR classicalm[Title/Abstract] OR classicalmusic[Title/Abstract] OR classicalones[Title/Abstract] OR classicalor[Title/Abstract] OR classicalquantum[Title/Abstract] OR classicalregulation[Title/Abstract] OR classicals[Title/Abstract] OR classicalsurgical[Title/Abstract] OR classicaltrade[Title/Abstract] OR classicaly[Title/Abstract] OR classicamente[Title/Abstract] OR classication[Title/Abstract] OR classications[Title/Abstract] OR classicc[Title/Abstract] OR classicclinicopathological[Title/Abstract] OR classicepidermolytic[Title/Abstract] OR classicfication[Title/Abstract] OR classicform[Title/Abstract] OR classici[Title/Abstract] OR classicial[Title/Abstract] OR classicially[Title/Abstract] OR classiciation[Title/Abstract] OR classicication[Title/Abstract] OR classicification[Title/Abstract] OR classicism[Title/Abstract] OR classicist[Title/Abstract] OR classicist'[Title/Abstract] OR classicistic[Title/Abstract] OR classicists[Title/Abstract] OR classicists'[Title/Abstract] OR classickaposi[Title/Abstract] OR classicks[Title/Abstract] OR classicl[Title/Abstract] OR classiclal[Title/Abstract] OR classiclat[Title/Abstract] OR classicle[Title/Abstract] OR classicly[Title/Abstract] OR classico[Title/Abstract] OR classico'[Title/Abstract] OR classicor[Title/Abstract] OR classicos[Title/Abstract] OR classicperformers[Title/Abstract] OR classics[Title/Abstract] OR classics'[Title/Abstract] OR classictm[Title/Abstract] OR classictrade[Title/Abstract] OR classictriad[Title/Abstract] OR classicula[Title/Abstract] OR classiculaceae[Title/Abstract] OR classiculales[Title/Abstract] OR classiculasinensis[Title/Abstract] OR classiculomycetes[Title/Abstract] OR classicus[Title/Abstract] OR classid[Title/Abstract] OR classied[Title/Abstract] OR classier[Title/Abstract] OR classiers[Title/Abstract] OR classiest[Title/Abstract] OR classif[Title/Abstract] OR classif1[Title/Abstract] OR classifacation[Title/Abstract] OR classifacatory[Title/Abstract] OR classifaction[Title/Abstract] OR classifation[Title/Abstract] OR classifcation[Title/Abstract] OR classifcations[Title/Abstract] OR classifed[Title/Abstract] OR classifer[Title/Abstract] OR classifers[Title/Abstract] OR classiffication[Title/Abstract] OR classifi[Title/Abstract] OR classifiability[Title/Abstract] OR classifiable[Title/Abstract] OR classifiable'[Title/Abstract] OR classifiables[Title/Abstract] OR classifiably[Title/Abstract] OR classifiacation[Title/Abstract] OR classifiactory[Title/Abstract] OR classifiation[Title/Abstract] OR classific[Title/Abstract] OR classifica[Title/Abstract] OR classificable[Title/Abstract] OR classificacao[Title/Abstract] OR classificaci'on[Title/Abstract] OR classificacion[Title/Abstract] OR classificacoes[Title/Abstract] OR classificaction[Title/Abstract] OR classificada[Title/Abstract] OR classificadas[Title/Abstract] OR classificado[Title/Abstract] OR classificadores[Title/Abstract] OR classificados[Title/Abstract] OR classificaion[Title/Abstract] OR classificaiton[Title/Abstract] OR classifical[Title/Abstract] OR classifically[Title/Abstract] OR classificando[Title/Abstract] OR classificantion[Title/Abstract] OR classificar[Title/Abstract] OR classificaram[Title/Abstract] OR classificassion[Title/Abstract] OR classificastems[Title/Abstract] OR classificat[Title/Abstract] OR classificate[Title/Abstract] OR classificated[Title/Abstract] OR classificateur[Title/Abstract] OR classificatie[Title/Abstract] OR classificatied[Title/Abstract] OR classificatin[Title/Abstract] OR classificating[Title/Abstract] OR classificatio[Title/Abstract] OR classificatiom[Title/Abstract] OR classification[Title/Abstract] OR classification'[Title/Abstract] OR classification''[Title/Abstract] OR classification's[Title/Abstract] OR classification1[Title/Abstract] OR classification10[Title/Abstract] OR classification2[Title/Abstract] OR classification2015[Title/Abstract] OR classificationaccuracy[Title/Abstract] OR classificational[Title/Abstract] OR classificationand[Title/Abstract] OR classificationbased[Title/Abstract] OR classificationdifference[Title/Abstract] OR classificationfor[Title/Abstract] OR classificationi[Title/Abstract] OR classificationmethod[Title/Abstract] OR classificationmodels[Title/Abstract] OR classificationmtd[Title/Abstract] OR classificationof[Title/Abstract] OR classificationperformances[Title/Abstract] OR classificationprocess[Title/Abstract] OR classifications[Title/Abstract] OR classifications'[Title/Abstract] OR classificationtechniques[Title/Abstract] OR classificationthe[Title/Abstract] OR classificationtibia[Title/Abstract] OR classificationvirus[Title/Abstract] OR classificationwas[Title/Abstract] OR classificationwere[Title/Abstract] OR classificative[Title/Abstract] OR classificatoire[Title/Abstract] OR classificaton[Title/Abstract] OR classificator[Title/Abstract] OR classificatorio[Title/Abstract] OR classificators[Title/Abstract] OR classificatory[Title/Abstract] OR classificatoryand[Title/Abstract] OR classificazione[Title/Abstract] OR classificcation[Title/Abstract] OR classifice[Title/Abstract] OR classificed[Title/Abstract] OR classificetion[Title/Abstract] OR classificiation[Title/Abstract] OR classificitaion[Title/Abstract] OR classifictations[Title/Abstract] OR classifiction[Title/Abstract] OR classifid[Title/Abstract] OR classifided[Title/Abstract] OR classifie[Title/Abstract] OR classified[Title/Abstract] OR classified'[Title/Abstract] OR classified78[Title/Abstract] OR classifiedand[Title/Abstract] OR classifiedas[Title/Abstract] OR classifiedinto[Title/Abstract] OR classifiedl[Title/Abstract] OR classifiedly[Title/Abstract] OR classifieds[Title/Abstract] OR classifiedz[Title/Abstract] OR classifield[Title/Abstract] OR classifier[Title/Abstract] OR classifier'[Title/Abstract] OR classifier's[Title/Abstract] OR classifier1[Title/Abstract] OR classifier2[Title/Abstract] OR classifiera[Title/Abstract] OR classifierpre[Title/Abstract] OR classifiers[Title/Abstract] OR classifiers'[Title/Abstract] OR classifiersfor[Title/Abstract] OR classifierssuch[Title/Abstract] OR classifiertrade[Title/Abstract] OR classifies[Title/Abstract] OR classififed[Title/Abstract] OR classifification[Title/Abstract] OR classifified[Title/Abstract] OR classifiied[Title/Abstract] OR classifikation[Title/Abstract] OR classifilcation[Title/Abstract] OR classifing[Title/Abstract] OR classifised[Title/Abstract] OR classifited[Title/Abstract] OR classifixation[Title/Abstract] OR classifiy[Title/Abstract] OR classifiying[Title/Abstract] OR classifled[Title/Abstract] OR classify[Title/Abstract] OR classify'[Title/Abstract] OR classifyability[Title/Abstract] OR classifycation[Title/Abstract] OR classifyied[Title/Abstract] OR classifying[Title/Abstract] OR classifying'[Title/Abstract] OR classifyme[Title/Abstract] OR classifynder[Title/Abstract] OR classifynder's[Title/Abstract] OR classifyr[Title/Abstract] OR classigying[Title/Abstract] OR classihcation[Title/Abstract] OR classii[Title/Abstract] OR classii2[Title/Abstract] OR classiib[Title/Abstract] OR classiical[Title/Abstract] OR classiication[Title/Abstract] OR classiicytokine[Title/Abstract] OR classiied[Title/Abstract] OR classiifcation[Title/Abstract] OR classiification[Title/Abstract] OR classiii[Title/Abstract] OR classika[Title/Abstract] OR classilled[Title/Abstract] OR classily[Title/Abstract] OR classimat[Title/Abstract] OR classimplant[Title/Abstract] OR classimport[Title/Abstract] OR classin[Title/Abstract] OR classiness[Title/Abstract] OR classing[Title/Abstract] OR classiology[Title/Abstract] OR classiphage[Title/Abstract] OR classiphy[Title/Abstract] OR classique[Title/Abstract] OR classique'[Title/Abstract] OR classiquely[Title/Abstract] OR classiques[Title/Abstract] OR classis[Title/Abstract] OR classisfied[Title/Abstract] OR classism[Title/Abstract] OR classism'[Title/Abstract] OR classist[Title/Abstract] OR classit[Title/Abstract] OR classitic[Title/Abstract] OR classitication[Title/Abstract] OR classitied[Title/Abstract] OR classiv[Title/Abstract] OR classivication[Title/Abstract] OR classix[Title/Abstract] OR classk[Title/Abstract] OR classkv[Title/Abstract] OR classless[Title/Abstract] OR classlessness[Title/Abstract] OR classlfication[Title/Abstract] OR classlib[Title/Abstract] OR classlow[Title/Abstract] OR classmaker[Title/Abstract] OR classmalocclusion[Title/Abstract] OR classman[Title/Abstract] OR classmate[Title/Abstract] OR classmate'[Title/Abstract] OR classmate's[Title/Abstract] OR classmates[Title/Abstract] OR classmates'[Title/Abstract] OR classmen[Title/Abstract] OR classment[Title/Abstract] OR classmode[Title/Abstract] OR classmolecules[Title/Abstract] OR classness[Title/Abstract] OR classo[Title/Abstract] OR classof[Title/Abstract] OR classon[Title/Abstract] OR classone[Title/Abstract] OR classopollis[Title/Abstract] OR classorii[Title/Abstract] OR classpharmer[Title/Abstract] OR classrbm[Title/Abstract] OR classrelated[Title/Abstract] OR classrho[Title/Abstract] OR classroms[Title/Abstract] OR classroom[Title/Abstract] OR classroom'[Title/Abstract] OR classroom's[Title/Abstract] OR classroomclimate[Title/Abstract] OR classroomformat[Title/Abstract] OR classroomlike[Title/Abstract] OR classrooms[Title/Abstract] OR classrooms'[Title/Abstract] OR classroomwide[Title/Abstract] OR classrooom[Title/Abstract] OR classrtoom[Title/Abstract] OR classs[Title/Abstract] OR classses[Title/Abstract] OR classsical[Title/Abstract] OR classsication[Title/Abstract] OR classsifcation[Title/Abstract] OR classsification[Title/Abstract] OR classsified[Title/Abstract] OR classsnitch[Title/Abstract] OR classspecific[Title/Abstract] OR classss[Title/Abstract] OR classstab[Title/Abstract] OR classthrombus[Title/Abstract] OR classtime[Title/Abstract] OR classtm[Title/Abstract] OR classtr[Title/Abstract] OR classtumor[Title/Abstract] OR classtype[Title/Abstract] OR classtypes[Title/Abstract] OR classu2b[Title/Abstract] OR classu3a[Title/Abstract] OR classum[Title/Abstract] OR classv[Title/Abstract] OR classvii[Title/Abstract] OR classwide[Title/Abstract] OR classwise[Title/Abstract] OR classwomen[Title/Abstract] OR classwork[Title/Abstract] OR classxagexheart[Title/Abstract] OR classxfollicular[Title/Abstract] OR classxgender[Title/Abstract] OR classy[Title/Abstract] OR classy1[Title/Abstract] OR classyfire[Title/Abstract] OR classyfire's[Title/Abstract] OR classyflu[Title/Abstract] OR classys[Title/Abstract]) OR (profil[Title/Abstract] OR profil1[Title/Abstract] OR profilable[Title/Abstract] OR profilac[Title/Abstract] OR profilacted[Title/Abstract] OR profilactic[Title/Abstract] OR profilactica[Title/Abstract] OR profilactically[Title/Abstract] OR profilactico[Title/Abstract] OR profilactics[Title/Abstract] OR profilactin[Title/Abstract] OR profilage[Title/Abstract] OR profilaggrin[Title/Abstract] OR profilaggrin306[Title/Abstract] OR profilaggrins[Title/Abstract] OR profilakticheskaia[Title/Abstract] OR profilaktika[Title/Abstract] OR profilaktike[Title/Abstract] OR profilaktycznych[Title/Abstract] OR profilaktyki[Title/Abstract] OR profilament[Title/Abstract] OR profilamentous[Title/Abstract] OR profilaments[Title/Abstract] OR profilanalyse[Title/Abstract] OR profilarinema[Title/Abstract] OR profilassi[Title/Abstract] OR profilate[Title/Abstract] OR profilated[Title/Abstract] OR profilatic[Title/Abstract] OR profilatica[Title/Abstract] OR profilatico[Title/Abstract] OR profilation[Title/Abstract] OR profilattico[Title/Abstract] OR profilaxa[Title/Abstract] OR profilaxia[Title/Abstract] OR profilaxis[Title/Abstract] OR profilaxy[Title/Abstract] OR profilaxys[Title/Abstract] OR profile[Title/Abstract] OR profile'[Title/Abstract] OR profile''[Title/Abstract] OR profile's[Title/Abstract] OR profile1[Title/Abstract] OR profile14[Title/Abstract] OR profileacid[Title/Abstract] OR profilealignment[Title/Abstract] OR profileanalysis[Title/Abstract] OR profileatphad[Title/Abstract] OR profilebased[Title/Abstract] OR profilebranching[Title/Abstract] OR profilechaser[Title/Abstract] OR profiled[Title/Abstract] OR profiled'[Title/Abstract] OR profiledata[Title/Abstract] OR profiledb[Title/Abstract] OR profiledriven[Title/Abstract] OR profilees[Title/Abstract] OR profileferator[Title/Abstract] OR profilefingerprints[Title/Abstract] OR profilefor[Title/Abstract] OR profilegraph[Title/Abstract] OR profilegrid[Title/Abstract] OR profilegrids[Title/Abstract] OR profilein[Title/Abstract] OR profileincompatibility[Title/Abstract] OR profileing[Title/Abstract] OR profileittrade[Title/Abstract] OR profilemake[Title/Abstract] OR profilemaker[Title/Abstract] OR profilemeasurements[Title/Abstract] OR profilemembers[Title/Abstract] OR profilemeter[Title/Abstract] OR profilemetrics[Title/Abstract] OR profilemetry[Title/Abstract] OR profilen[Title/Abstract] OR profileness[Title/Abstract] OR profilenj[Title/Abstract] OR profileobserved[Title/Abstract] OR profileof[Title/Abstract] OR profileometer[Title/Abstract] OR profileometric[Title/Abstract] OR profileometry[Title/Abstract] OR profilepeople[Title/Abstract] OR profilepi[Title/Abstract] OR profileplasma[Title/Abstract] OR profileplasty[Title/Abstract] OR profilepstmm[Title/Abstract] OR profilequanttrade[Title/Abstract] OR profiler[Title/Abstract] OR profiler'[Title/Abstract] OR profiler's[Title/Abstract] OR profiler2[Title/Abstract] OR profiler725[Title/Abstract] OR profilerance[Title/Abstract] OR profilerated[Title/Abstract] OR profilerating[Title/Abstract] OR profileration[Title/Abstract] OR profilerations[Title/Abstract] OR profilerative[Title/Abstract] OR profilerator[Title/Abstract] OR profilereflective[Title/Abstract] OR profileres[Title/Abstract] OR profilerplus[Title/Abstract] OR profilers[Title/Abstract] OR profilers'[Title/Abstract] OR profilertm[Title/Abstract] OR profilertrade[Title/Abstract] OR profiles[Title/Abstract] OR profiles'[Title/Abstract] OR profiles''[Title/Abstract] OR profiles's[Title/Abstract] OR profilesacross[Title/Abstract] OR profilesagainst[Title/Abstract] OR profilesand[Title/Abstract] OR profilescan[Title/Abstract] OR profilescope[Title/Abstract] OR profilesdagger[Title/Abstract] OR profilesearch[Title/Abstract] OR profileseq[Title/Abstract] OR profilesfurther[Title/Abstract] OR profilesimilar[Title/Abstract] OR profilesin[Title/Abstract] OR profilesnumbers[Title/Abstract] OR profilesor[Title/Abstract] OR profilesprincipal[Title/Abstract] OR profilesregistry[Title/Abstract] OR profilet[Title/Abstract] OR profilethat[Title/Abstract] OR profileto[Title/Abstract] OR profiletrade[Title/Abstract] OR profileurine[Title/Abstract] OR profileviewer[Title/Abstract] OR profilevortex[Title/Abstract] OR profileweight[Title/Abstract] OR profilewith[Title/Abstract] OR profilggrin[Title/Abstract] OR profili[Title/Abstract] OR profiliation[Title/Abstract] OR profilic[Title/Abstract] OR profilicollis[Title/Abstract] OR profilies[Title/Abstract] OR profilig[Title/Abstract] OR profilimetry[Title/Abstract] OR profilin[Title/Abstract] OR profilin's[Title/Abstract] OR profilin1[Title/Abstract] OR profilin1's[Title/Abstract] OR profilin2[Title/Abstract] OR profilin2a[Title/Abstract] OR profilin3[Title/Abstract] OR profilin4[Title/Abstract] OR profilincis[Title/Abstract] OR profiling[Title/Abstract] OR profiling'[Title/Abstract] OR profilingdagger[Title/Abstract] OR profilingof[Title/Abstract] OR profilings[Title/Abstract] OR profilingtrade[Title/Abstract] OR profilingusing[Title/Abstract] OR profilinic[Title/Abstract] OR profilinii[Title/Abstract] OR profiliniia[Title/Abstract] OR profilins[Title/Abstract] OR profilins'[Title/Abstract] OR profiliocollis[Title/Abstract] OR profilis[Title/Abstract] OR profility[Title/Abstract] OR profilization[Title/Abstract] OR profill[Title/Abstract] OR profillagrin[Title/Abstract] OR profiller[Title/Abstract] OR profilles[Title/Abstract] OR profillin[Title/Abstract] OR profilling[Title/Abstract] OR profilmeter[Title/Abstract] OR profilmetry[Title/Abstract] OR profilnine[Title/Abstract] OR profilninesd[Title/Abstract] OR profilo[Title/Abstract] OR profiloat[Title/Abstract] OR profilocephalometric[Title/Abstract] OR profiloesophagotonometry[Title/Abstract] OR profilogram[Title/Abstract] OR profilograms[Title/Abstract] OR profilograph[Title/Abstract] OR profilographic[Title/Abstract] OR profilography[Title/Abstract] OR profilom[Title/Abstract] OR profilomat[Title/Abstract] OR profilometar[Title/Abstract] OR profilometer[Title/Abstract] OR profilometer's[Title/Abstract] OR profilometers[Title/Abstract] OR profilometery[Title/Abstract] OR profilometre[Title/Abstract] OR profilometric[Title/Abstract] OR profilometric'[Title/Abstract] OR profilometrical[Title/Abstract] OR profilometrically[Title/Abstract] OR profilometricaly[Title/Abstract] OR profilometrie[Title/Abstract] OR profilometries[Title/Abstract] OR profilometry[Title/Abstract] OR profilometry'[Title/Abstract] OR profilometty[Title/Abstract] OR profilomg[Title/Abstract] OR profilomotor[Title/Abstract] OR profiloplasty[Title/Abstract] OR profiloscope[Title/Abstract] OR profilplasty[Title/Abstract] OR profils[Title/Abstract] OR profilses[Title/Abstract] OR profiltrates[Title/Abstract] OR profilyzer[Title/Abstract]) OR (phenotyp[Title/Abstract] OR phenotyp3[Title/Abstract] OR phenotypage[Title/Abstract] OR phenotypal[Title/Abstract] OR phenotypcs[Title/Abstract] OR phenotype[Title/Abstract] OR phenotype'[Title/Abstract] OR phenotype''[Title/Abstract] OR phenotype's[Title/Abstract] OR phenotype1[Title/Abstract] OR phenotype12[Title/Abstract] OR phenotypeable[Title/Abstract] OR phenotypeannotation[Title/Abstract] OR phenotypecally[Title/Abstract] OR phenotypecorrelation[Title/Abstract] OR phenotyped[Title/Abstract] OR phenotyped'[Title/Abstract] OR phenotypedriven[Title/Abstract] OR phenotypefr[Title/Abstract] OR phenotypefrom[Title/Abstract] OR phenotypegene[Title/Abstract] OR phenotypegenotype[Title/Abstract] OR phenotypeic[Title/Abstract] OR phenotypein[Title/Abstract] OR phenotypeinvolves[Title/Abstract] OR phenotypeis[Title/Abstract] OR phenotypeless[Title/Abstract] OR phenotypelevel[Title/Abstract] OR phenotypemarkers[Title/Abstract] OR phenotypemediated[Title/Abstract] OR phenotypen[Title/Abstract] OR phenotypeof[Title/Abstract] OR phenotypepatuents[Title/Abstract] OR phenotypeportal[Title/Abstract] OR phenotyper[Title/Abstract] OR phenotyperemodeling[Title/Abstract] OR phenotyperesulted[Title/Abstract] OR phenotyperevealed[Title/Abstract] OR phenotypers[Title/Abstract] OR phenotypers'[Title/Abstract] OR phenotypes[Title/Abstract] OR phenotypes'[Title/Abstract] OR phenotypes''[Title/Abstract] OR phenotypes1[Title/Abstract] OR phenotypesand[Title/Abstract] OR phenotypeseeker[Title/Abstract] OR phenotypesimulator[Title/Abstract] OR phenotypesin[Title/Abstract] OR phenotypespecific[Title/Abstract] OR phenotypesr[Title/Abstract] OR phenotypesre[Title/Abstract] OR phenotypess[Title/Abstract] OR phenotypesthe[Title/Abstract] OR phenotypesthese[Title/Abstract] OR phenotypesto[Title/Abstract] OR phenotypetargeted[Title/Abstract] OR phenotypetrade[Title/Abstract] OR phenotypetranscriptionally[Title/Abstract] OR phenotypevana[Title/Abstract] OR phenotypexdiet[Title/Abstract] OR phenotypexsurgery[Title/Abstract] OR phenotypextime[Title/Abstract] OR phenotyphes[Title/Abstract] OR phenotyphic[Title/Abstract] OR phenotyphically[Title/Abstract] OR phenotyphicaly[Title/Abstract] OR phenotypia[Title/Abstract] OR phenotypially[Title/Abstract] OR phenotypic[Title/Abstract] OR phenotypic'[Title/Abstract] OR phenotypic2[Title/Abstract] OR phenotypical[Title/Abstract] OR phenotypicality[Title/Abstract] OR phenotypically[Title/Abstract] OR phenotypically'[Title/Abstract] OR phenotypicallynotch[Title/Abstract] OR phenotypicallyobvious[Title/Abstract] OR phenotypicaly[Title/Abstract] OR phenotypicassays[Title/Abstract] OR phenotypice[Title/Abstract] OR phenotypicically[Title/Abstract] OR phenotypicidentification[Title/Abstract] OR phenotypics[Title/Abstract] OR phenotypictransition[Title/Abstract] OR phenotypie[Title/Abstract] OR phenotypies[Title/Abstract] OR phenotypification[Title/Abstract] OR phenotypificationwas[Title/Abstract] OR phenotypig[Title/Abstract] OR phenotyping[Title/Abstract] OR phenotyping'[Title/Abstract] OR phenotyping2[Title/Abstract] OR phenotypings[Title/Abstract] OR phenotypique[Title/Abstract] OR phenotypiques[Title/Abstract] OR phenotypisation[Title/Abstract] OR phenotypization[Title/Abstract] OR phenotyple[Title/Abstract] OR phenotypoc[Title/Abstract] OR phenotypological[Title/Abstract] OR phenotypology[Title/Abstract] OR phenotypos[Title/Abstract] OR phenotyppes[Title/Abstract] OR phenotyps[Title/Abstract] OR phenotyptes[Title/Abstract] OR phenotyptic[Title/Abstract] OR phenotypus[Title/Abstract] OR phenotypw[Title/Abstract] OR phenotypy[Title/Abstract] OR phenotypy'[Title/Abstract] OR phenotypying[Title/Abstract])) |

**2) Embase®**

('diabetes mellitus':ab,ti,kw OR t2dm:ab,ti,kw OR 'insulin dependent diabetes mellitus':ab,ti,kw OR niddm:ab,ti,kw OR 'non insulin dependent diabetes mellitus'/exp) AND ('cluster analysis' OR cluster OR typolog* OR stratif* OR segment* OR class* OR profil* OR phenotyp*)

**3) SCOPUS®**

( TITLE-ABS-KEY ( ( "Diabetes Mellitus" OR "Diabetes Mellitus, Type 2" OR t2dm OR "type 2 diabetes mellitus" OR niddm ) ) AND TITLE-ABS-KEY ( ( 'cluster AND analysis' OR cluster* OR typolog* OR stratif* OR segment* OR class* OR profil* OR phenotyp* ) ) )

**4) PsycInfo®**

((diabetes mellitus) OR ((type 2) (diabetes mellitus)) OR t2dm OR NIDDM OR diabetes mellitus, type 2)) AND ('cluster analysis' OR cluster OR typology OR stratification OR segmentation OR class OR Profile OR Phenotype)
