# Supplementary material for: Population segmentation of type 2 diabetes mellitus patients and its clinical applications - a scoping review
Source: BMC Med Res Methodol. 2021 Mar 11;21:49. doi: 10.1186/s12874-021-01209-w (PMC7953703; doi:10.1186/s12874-021-01209-w)
Supplement: Supplementary file 2 — Additional file 2. Details of included studies [file 12874_2021_1209_MOESM2_ESM.docx]

**Supplementary File 2. Details of included studies**

| **S/No** | **Authors (Year)** | **Article Name** | **Continent** | **Country** | **Study design** | **Sample size** | **T2DM Patient population** | **Mean / Median Age of patients** | **Gender [Male (%)]** | **Duration of diabetes** | **Data source** | **Study setting** | **Objective of segmentation** | **Segmentation variables** | **Statistical methods used** | **Software** | **Number of segments** | **Categories of segments** |
| --- | --- | --- | --- | --- | --- | --- | --- | --- | --- | --- | --- | --- | --- | --- | --- | --- | --- | --- |
| 1 | Aguilar-Salinas CA et al (2001) | Early-onset type 2 diabetes: metabolic and genetic characterization in the mexican population. | North America | Mexico | Cross-sectional study | 40 | Adult T2DM patients between 20 and 40 years of age recruited from outpatient clinics | Expressed as Mean(SD) 1. Insulin Resistant (SI < 4): 35 (5.4) 2. Insulin Sensitive (SI > 4): 35 (8) | 1. Insulin Resistant (SI < 4): 8 (61.5%) 2. Insulin Sensitive (SI > 4): 21 (77.8%) | not specified | primary. lipid and clinical laboratory measurements | tertiary | Health grouping / profiling | 1. Subtypes of early T2DM | Judgemental splitting by patients' clinical characteristics | Stata, statistics/data analysis version 5.0. | 1. Subtypes of early T2DM: 2 | Subtypes of early T2DM 1. Insulin sensitive 2. Insulin resistant |
| 2 | Al-Mukhtar SB et al (2012) | General and gender characteristics of type 2 diabetes mellitus among the younger and older age groups | Asia | Iraq | Cross-sectional study | 432 | Adult T2DM patients (above and below 60 years) attending Outpatient Clinic at Al-Zahrawi Private Hospital in Mosul City-Iraq, | Expressed as Mean(SD) 1, Men < 60 years: 51.6(5.0) 2. Women < 60 years: 51.5(5.0) 3. Men >/= 60 years: 64.7(5.1)  4. Women >/= 60 years: 64.5(4.4) | 1. Men < 60 years: 167 (53.0%) 2. Men >/= 60 years: 77 (52.4%) | Expressed as Mean(SD) 1. Men < 60 years: 7.4(4.1) 2. Women < 60 years: 7.4(3.8) 3. Men >/= 60 years: 4.8(3.4)  4. Women >/= 60 years: 3.9(2.7) | Primary.  -Analyzed in regard to age, duration of diabetes, smoking, socioeconomic status, anthropometric indices, blood pressure, fasting plasma glucose, glycated hemoglobin A1c and serum lipids. - Questionnaires to determine smoking status | tertiary | Health grouping/profiling | 1. Patients' gender 2. Patients' age | Judgemental Splitting by patients' sociodemographic and/or economic attributes | not specified | 4 | 1. Men < 60 years 2. Women < 60 years 3. Men >= 60 years 4. Women >= 60 years |
| 3 | Amato MC et al (2016) | Phenotyping of type 2 diabetes mellitus at onset on the basis of fasting incretin tone: Results of a two-step cluster analysis | Europe | Italy | Cross-sectional study | 96 | Adult T2DM patients within 6 months of onset and in stable treatment for the last 3 months with metformin | Expressed as Mean(SD) 62.40 (6.36) | 1. Cluster 1: 30 (47.6%) 2. Cluster 2: 15 (45.5%) | Expressed as Mean(SD) 4.41 (1.66) | primary. A complete medical history, a complete physical examination, bodyweight, height, and waist and hip circumference were taken | tertiary | Health Grouping/Profiling | 1. Levels of GLP-1, GIP and ghrelin | Cluster analysis | SPSS version 17 | 2 | 1. Cluster 1: Lower levels of GLP-1, GIP and ghrelin  2. Cluster 2: Higher levels of GLP-1, GIP and ghrelin |
| **S/No** | **Authors (Year)** | **Article Name** | **Continent** | **Country** | **Study design** | **Sample size** | **T2DM Patient population** | **Mean / Median Age of patients** | **Gender [Male (%)]** | **Duration of diabetes** | **Data source** | **Study setting** | **Objective of segmentation** | **Segmentation variables** | **Statistical methods used** | **Software** | **Number of segments** | **Categories of segments** |
| 4 | Amutha A et al (2012) | Clinical profile and complications of childhood- and adolescent-onset type 2 diabetes seen at a diabetes center in south India. | Asia | India | Cross-sectional study | 368 | Patients diagnosed with childhood- and adolescent-onset T2DM | Expressed as Mean(SD) 22.2(9.7) | 168 (45.7%) | Expressed as Mean(SD) 5.94 (0.48) | Primary  Clinical assessment, questionnaires and blood tests | tertiary | - Health Grouping/Profiling  - Assess differential risk of diabetic related complications across groups | 1. Glutamic acid decarboxylase status (positive / negative) | Judgemental splitting by patients' clinical characteristics | SPSS for Windows version 15.0 | 2 | 1. GAD positive 2. GAD negative |
| 5 | Barrot-de la Puente J et al (2015) | Older type 2 diabetic patients are more likely to achieve glycaemic and cardiovascular risk factors targets than younger patients: analysis of a primary care database | Europe | Spain | Cross-sectional study | 318020 | Adult T2DM patients aged ≥ 30 years who were diagnosed with T2DM by 31 December 2011 and attended a primary care centre during 2011 | Expressed as Mean (SD) Men Total: 66.9 (11.7)  1. <= 65 years: 56.2 (7.0) 2. 66-75 years: 70.3 (2.9) 3. 76-85 years: 79.8 (2.7) 4. > 85 years: 88.4 (2.5)  Women Total: 71.1 (11.8)  1. <= 65 years: 56.8 (7.0) 2. 66-75 years: 70.6 (2.9)  3. 76-85 years: 80.1 (2.8) 4. >85 years: 89.0 (2.8) | Total: 171219 (53.8%) 1. <= 65 years: 75986 (44.4%) 2. 66-75 years: 50912 (29.7%) 3. 76-85 years: 37090 (21.7%) 4. > 85 years: 7231 (4.22%) | Expressed as Mean (SD) Men Total: 6.9 (5.2) 1. <= 65 years: 5.6 (4.2) 2. 66-75 years: 7.5 (5.2) 3. 76-85 years: 8.5 (5.9) 4. > 85 years: 9.0 (6.5)  Women Total: 7.7 (5.7) 1. <= 65 years: 5.9 (4.5) 2. 66-75 years: 7.8 (5.5)  3. 76-85 years: 8.8 (6.2) 4. >85 years: 9.1 (6.5) | Secondary data Administrative healthcare database | Primary | Assess diabetic control across groups  Assess non-diabetes metabolic derangements (e.g. lipid, blood pressure) across groups | 1. Patients' age 2. Patients' gender | Judgemental Splitting by patients' sociodemographic and/or economic attributes | Stata/SE version 13 for Windows | 8 | 1. Men (=<65 years old) 2. Men (66 - 75 years old) 3. Men (76 - 85 years old) 4. Men (>85 years old) 5. Women (=<65 years old) 6. Women (66 - 75 years old) 7. Women (76 - 85 years old) 8. Women (>85 years old) |
| 6 | Basanta-Alario ML et al (2016) | Differences in clinical and biological characteristics and prevalence of chronic complications related to aging in patients with type 2 diabetes. | Europe | Spain | Cross-sectional study | 405 | Adult T2DM patients age over 18 years with diagnosis of T2DM based on basal blood glucose levels ≥126 mg/dL (measured twice) or glycosy-lated hemoglobin (HbA1c) levels ≥6.5%. | Expressed as Mean(SD) All: 65.97 (10.43) 1. Group 1 (<60 years): 53.05 (6.11) 2. Group 2 (>70 years): 78.49 (4.21) | 1. Group 1 (<60 years): 42 (38.3%) 2. Group 2 (>70 years): 59 (52.7%) | Expressed as Mean(SD) All: 12.67 (9.67) 1. Group 1 (<60 years): 10.07 (8.36) 2. Group 2 (>70 years): 13.96 (10.1) | -primary -physical examination and biochemical variables measured in peripheral blood | -tertiary | - Assess mortality | 1. Patients' age | Judgemental Splitting by patients' sociodemographic and/or economic attributes | Not specified | 2 | 1. Group 1 (<60 years) 2. Group 2 (>70 years) |
| 7 | Benhalima K et al (2011) | Type 2 diabetes in younger adults: clinical characteristics, diabetes-related complications and management of risk factors. | Europe | England | Retrospective cohort study | 185 | Adult T2DM patients aged <35 years old attending a specialist diabetes clinic | Expressed in Mean(SD) 24.5 ( 5.5) | 63 (35%) | Expressed in Mean(SD) 4.5 (3.6) | - Secondary data - Administrative healthcare records | Tertiary | - Health grouping / profiling - Assess differential risk of diabetic related complications across groups - Assess diabetic control across groups  - Assess non-diabetes metabolic derangements (e.g. lipid, blood pressure) across groups | 1. Gender 2. Ethnicity of patients | Judgemental Splitting by patients' sociodemographic and/or economic attributes | SPSS 16.0 | 2 | Ethnicity of patients 1. Caucasian 2. Ethnic minority backgrounds  Gender of patients 1. Male 2. Female |
| **S/No** | **Authors (Year)** | **Article Name** | **Continent** | **Country** | **Study design** | **Sample size** | **T2DM Patient population** | **Mean / Median Age of patients** | **Gender [Male (%)]** | **Duration of diabetes** | **Data source** | **Study setting** | **Objective of segmentation** | **Segmentation variables** | **Statistical methods used** | **Software** | **Number of segments** | **Categories of segments** |
| 8 | Berkowitz SA et al (2013) | Age at type 2 diabetes onset and glycaemic control results from the National Health and Nutrition Examination Survey (NHANES) 2005-2010. | North America | United States of America | Cross-sectional study | 1438 | All adult (Age > 20 years) NHANES participants from 2005–2010 with T2DM | Age at diagnosis, expressed as n(%) 1. <65 years old: 1204 (83.7%) 2. >=65 years old: 234 (16.3%) Current age, expressed as Mean(SD): 59.6 (0.5) | Overall: 700 (48.7%)  1. <65 years old at diagnosis 577 (49.5%)  2. >=65 years old at diagnosis 122 (44.7%) | Not specified | Primary data National Health and Nutrition Examination Survey | Primary | Assess diabetic control across groups  Assess mortality | 1. Age at time of diagnosis | Judgemental splitting by patients' clinical characteristics | SAS version 9.3 | 2 | 1. <65 years at diabetes diagnosis 2. ≥65 years at diabetes diagnosis |
| 9 | Berry E et al (2017) | Illness perception clusters and relationship quality are associated with diabetes distress in adults with Type 2 diabetes | Europe | Ireland | Cross-sectional study | 162 | Adult T2DM patients | Expressed as Mean(SD) 68.29 (10.84) | 107 (66%) | Expressed as Mean(SD)  10.18 (8.004) | primary: Questionnaire and HbA1c measurement | primary | Assess differential risk of psychological outcomes across groups | 1. Illness perception | Cluster analysis | SPSS version 21 | 3 | 1. Cluster 1: Believe that diabetes has severe consequences on daily life, have a strong experience of diabetes symptoms, and feel that these symptoms are unpredictable. Felt that they could not adequately control their diabetes.  2. Cluster 2: Do not have a strong experience of diabetes symptoms, and any symptoms experienced are perceived as infrequent. Do not believe that the diabetes has severe consequences on daily life and believe that they are able to effectively influence their diabetes.  3. Cluster 3: Do not have a strong experience of diabetes symptoms, but who believe that their diabetes is a serious and long-lasting condition. |
| 10 | Bidel S et al (2006) | Coffee consumption and risk of total and cardiovascular mortality among patients with type 2 diabetes. | Europe | Finland | Prospective cohort study | 3837 | T2DM Finnish patients aged 25 to 74 years old with diagnosis of T2DM made between 1968 and 2002 | Expressed as Mean(SD) 1. 0-2 cups of coffee daily: 48.8 (11.3) 2. 3-4 cups of coffee daily: 49.7 (10.3) 3. 5-6 cups of coffee daily: 49.4 (10.3)  4. >= 7 cups of coffee daily: 47.8 (9.9) | 1. 0-2 cups of coffee daily: 340 (52.8%) 2. 3-4 cups of coffee daily: 431 (41.4%) 3. 5-6 cups of coffee daily: 616 (45.4%) 4. >= 7 cups of coffee daily: 491 (61.7%) | not specified | -primary -Self administered questionnaire, clinical data | tertiary | - Assess differential risk of diabetic related complications across groups - Assess non-diabetes metabolic derangements (e.g. lipid, blood pressure) across groups | 1. Daily coffee consumption (no. of cups) | Judgemental splitting based on patients' lifestyle habits | SPSS for windows 13.0 | 4 | 1. 0-2 cups of coffee daily 2. 3-4 cups of coffee daily 3. 5-6 cups of coffee daily 4. >= 7 cups of coffee daily |
| **S/No** | **Authors (Year)** | **Article Name** | **Continent** | **Country** | **Study design** | **Sample size** | **T2DM Patient population** | **Mean / Median Age of patients** | **Gender [Male (%)]** | **Duration of diabetes** | **Data source** | **Study setting** | **Objective of segmentation** | **Segmentation variables** | **Statistical methods used** | **Software** | **Number of segments** | **Categories of segments** |
| 11 | Blak BT et al (2016) | Weight change and healthcare resource use in English patients with type 2 diabetes mellitus initiating a new diabetes medication class. | Europe | United Kingdom | Retrospective cohort study | 42,920 | Adult T2DM patients age ≥ 18 years old with a valid HbA1c value and weight value on or within 90 days prior to inclusion and initiated treatment with a new diabetes medication class between 2005 and 2012 | Expressed in Mean and 95% CI 1. Higher weight loss: 59 (95% CI: 58-60) 2. Moderate weight loss: 58 (95% CI: 59-61) 3. Weight neutral: 59 (95% CI: 59-60) 4. Moderate weight gain: 60 (95% CI: 59-61) 5. Higher weight gain: 58 (95% CI:57-59) | 1. Higher weight loss: 431 (44.7%) 2. Moderate weight loss: 620 (53.5%) 3. Weight neutral: 2902 (59.2%) 4. Moderate weight gain: 691 (63.5%) 5. Higher weight gain: 541 (58.9%) | Expressed in Mean and 95% CI 1. Higher weight loss: 3.4 years (95% CI: 3.0-3.7) 2. Moderate weight loss: 3.2 years (95% CI: 3.0-3.5) 3. Weight neutral: 3.6 years (95% CI: 3.5-3.7) 4. Moderate weight gain: 4.6 years (95% CI: 4.3-4.9) 5. Higher weight gain: 4.2 years (95% CI: 3.8-4.5) | Secondary. Administrative healthcare records | Primary | Assess differential healthcare utilization | 1. Weight change between the first and second weight measure (3-9 months later) | Judgemental splitting by patients' clinical characteristics | SAS version 9.2 | 5 | 1. Higher weight loss (>=5.5% loss) 2. Moderate weight loss (3.0-5.4% loss) 3. Weight neutral (<3% weight change) 4. Moderate weight gain (3.0-5.4% weight gain) 5. Higher weight gain (>=5.5% weight gain) |
| 12 | Bo S et al (2013) | Mortality outcomes of different sulphonylurea drugs: the results of a 14-year cohort study of type 2 diabetic patients. | Europe | Italy | Retrospective cohort study | 1277 | Adult T2DM patients attending the diabetes clinic in Asti | (At baseline enrolment)  Expressed in Mean(SD) 1. Glibenclamide: 65.3 (10.1)  2. Gliclazide: 66.4 (9.1)  3. Tolbutamide: 67.7 (10.7) | (At baseline enrolment)  1. Glibenclamide: 412 (42.2%) 2. Gliclazide: 63 (44.7%) 3. Tolbutamide: 73 (45.9%) | (At baseline enrolment)  Expressed in Median (IQR) 1. Glibenclamide: 10 (11) 2. Gliclazide: 8 (10) 3. Tolbutamide: 7 (10) | primary, baseline data from clinical records, Arterial blood pressure, Retinopathy diagnosed via ophthalmoscopic examination and/or retinal photography | tertiary (diabetes clinic) | - Assess mortality - Assess treatment outcomes | 1. Type of sulphonylurea used at enrolment | Judgemental splitting by patients' clinical characteristics | Stata 11.2 and R (2.15.0). | 3 | 1. Glibenclamide used at enrolment 2. Gliclazide used at enrolment 3. Tolbutamide used at enrolment |
| 13 | Bruce DG et al (2000) | Glycemic control in older subjects with type Glycemic control in older subjects with type 2 diabetes mellitus in the Fremantle Diabetes Study. | Australia | Australia | Cross-sectional study | 1205 | Adult T2DM patients aged over 40 years | Expressed as n(%) (by age category)  1. 40-49 years old: 75 (6.22%) 2. 50-59 years old: 253 (21.0%) 3. 60-69 years old: 473 (39.3%) 4. 70-79 years old: 321 (26.6%) 5. 80+ years old: 83 (6.89%) | 1. 40-49 years old: 35 (46.7%) 2. 50-59 years old: 120 (47.4%) 3. 60-69 years old: 247 (52.2%) 4. 70-79 years old: 150 (46.7%) 5. 80+ years old: 30 (36.1%) | Expressed as Median (Range) 1. 40-49 years old: 1.1 (0 - 7.8 years) 2. 50-59 years old: 2.8 (0 - 19.8 years) 3. 60-69 years old: 4.8 (0 - 26.1 years) 4. 70-79 years old: 5.2 (0 - 34.6 years) 5. 80+ years old: 5.5 (0.2 - 39.3 years) | Secondary data Administrative healthcare records | Tertiary | Assess diabetic control across groups  Assess non-diabetes metabolic derangements (e.g. lipid, blood pressure) across groups | 1. Patients' age | Judgemental Splitting by patients' sociodemographic and/or economic attributes | SPSS for Windows statistical program | 3 | 1. 40-69 years old  2. 70-79 years old 3. >=80 years old |
| 14 | Bruce DG et al (2016) | Comorbid Anxiety and Depression and Their Impact on Cardiovascular Disease in Type 2 Diabetes: The Fremantle Diabetes Study Phase II. | Australia | Australia | Prospective cohort study | 1337 | Adult T2DM patients residing in the study catchment area (postcode definted geographical area surrounding the port city of fremantle in the state of western australia) | Expressed as Mean(SD) 64.9 (14.4) | 722 (54%) | Expressed as Median(IQR) 10.0 (3.0–17.8) | -primary -baseline clinical assessment including questionnaire, physically examination and fasting biochemical tests | not specified | - Health Grouping/Profiling - Assess differential risk of diabetic related complications across groups - Assess non-diabetes metabolic derangements (e.g. lipid, blood pressure) across groups | 1. Presence and degree of anxiety and/or depression | Latent class analysis | IBM SPSS Statistics 21 | 4 | 1. No anxious depression 2. Subsyndromal anxiety 3. Minor anxious depression 4. Major anxious depression |
| **S/No** | **Authors (Year)** | **Article Name** | **Continent** | **Country** | **Study design** | **Sample size** | **T2DM Patient population** | **Mean / Median Age of patients** | **Gender [Male (%)]** | **Duration of diabetes** | **Data source** | **Study setting** | **Objective of segmentation** | **Segmentation variables** | **Statistical methods used** | **Software** | **Number of segments** | **Categories of segments** |
| 15 | Bruno G et al (1999) | Cardiovascular risk profile of type 2 diabetic patients cared for by general practitioners or at a diabetes clinic: a population-based study. | Europe | Italy | Cross-sectional study | 1574 | Italian patients with T2DM cared for by GPs and diabetologists, with clinic visit of 2 or more times in a year | Expressed as Mean(SD) 1. General Practitioners: 71.6 (10.6) 2. Diabetes clinic: 68.5 (10.5) | 1. General Practitioners: 155 (41.8%) 2. Diabetes clinic: 529 (44.4%) | Expressed as Mean(SD) 1. General Practitioners: 11.4 (6.7) 2. Diabetes clinic: 11.4 (7.0) | -Secondary Data -From multiple sources: Diabetes clinic, GPs, hospital discharges, prescriptions and sale records of reagent strips and syringes | -Primary and tertiary | 1) Health grouping/profiling | 1. Type of care provider | Judgemental splitting by patients' clinical characteristics | SAS softward version 6.10 | 2 | 1. General Practitioner 2. Diabetes clinic |
| 16 | Chan JC et al (2014) | Premature mortality and comorbidities in young-onset diabetes: a 7-year prospective analysis. | Asia | Hong Kong | prospective cohort study | 9509 | Adult T2DM patients who were referred to the hospital for comprehensive assessment of metabolic control and diabetes complications with young-onset diabetes was defined by age of diagnosis less than 40 years, and late-onset diabetes was defined by age of diabetes diagnosis at 40 years. | Expressed as Mean(SD) 1. Young-onset diabetes: 41.3 (9.8)  2. Late-onset diabetes: 61.9 (10.3) | 1. Young-onset diabetes: 9090 (44%) 2. Late-onset diabetes: 3534 (47.5%) | Expressed as Median (IQR) 1. Young-onset diabetes: 6 (1-13) 2, Late-onset diabetes: 5 (1-10) | Primary. Baseline Clinical and Laboratory Measurement | Tertiary | Assess differential risk of diabetic related complications across groups - Assess non-diabetes metabolic derangements (e.g. lipid, blood pressure) across groups | 1. Age of diabetes onset | Judgemental splitting by patients' clinical characteristics | SPSS version 15 | 2 | 1. Young-onset diabetes (age of diagnosis less than 40 years) 2. Late-onset diabetes (age of diabetes diagnosis at >= 40 years) |
| **S/No** | **Authors (Year)** | **Article Name** | **Continent** | **Country** | **Study design** | **Sample size** | **T2DM Patient population** | **Mean / Median Age of patients** | **Gender [Male (%)]** | **Duration of diabetes** | **Data source** | **Study setting** | **Objective of segmentation** | **Segmentation variables** | **Statistical methods used** | **Software** | **Number of segments** | **Categories of segments** |
| 17 | Chan KS et al (2012) | Do diabetic patients living in racially segregated neighborhoods experience different access and quality of care? | North America | United States of America | Cross-sectional study | 1598 | Adult T2DM patients from Medical Expenditure Panel Survey (MEPS) who were 18 years or older, had diabetes as one of their priority conditions and if they responded to the Diabetes Care Supplement. | Expressed as N(%) Total Sample (N=1598): Young Adult (18-24): 130 (0.82%)  Working Age (25-44): 195 (12.19%) Pre-retiree (45-64): 792 (49.55%) Elderly (65+): 598 (37.44%)  1. White (N= 774): Young Adult (18-24): 6 (0.78%) Working Age (25-44): 76 (9.82%) Pre-retiree (45-64): 372 (48.02%) Elderly (65+): 320 (41.38%)  2. Black (N=375): Young Adult (18-24): 4 (0.96%) Working Age (25-44): 52 (13.74%) Pre-retiree (45-64): 207 (55.2%) Elderly (65+): 113 (30.1%)  3. HIspanic (N=449): Young Adult (18-24): 4 (0.85%) Working Age (25-44): 89 (19.78%) Pre-retiree (45-64): 224 (49.86%) Elderly (65+): 132 (29.51%) | Total: 786 (49.2%) 1. White: 406 (52.4%) 2. Black: 159 (42.4%) 3. Hispanic: 195 (43.5%) | not specified | Secondary. Individual level socio-demographic, health and service use data were obtained from the 2006 MEPS (www.meps.ahrq.gov). Residential segregation data for Blacks, and Hispanics were abstracted at the Metropolitan Statistical Area or Primary Metropolitan Statistical Area (MSA/PMSA) level from the 2000 Census Residential Housing Patterns Dataset (www.census.gov). Data from the Area Resource File (www.arf.hrsa.gov) for 2006 on outpatient visit rate and number of hospital beds per 100 residents | primary and tertiary | Assess accessibility to providers and healthcare services | 1. Patients' race | Judgemental Splitting by patients' sociodemographic and/or economic attributes | STATA© 11 | 3 | 1. Non-Hispanic White 2. Non-Hispanic Black 3. Hispanic |
| **S/No** | **Authors (Year)** | **Article Name** | **Continent** | **Country** | **Study design** | **Sample size** | **T2DM Patient population** | **Mean / Median Age of patients** | **Gender [Male (%)]** | **Duration of diabetes** | **Data source** | **Study setting** | **Objective of segmentation** | **Segmentation variables** | **Statistical methods used** | **Software** | **Number of segments** | **Categories of segments** |
| 18 | Chao CT et al (2018) | Both pre-frailty and frailty increase healthcare utilization and adverse health outcomes in patients with type 2 diabetes mellitus. | Asia | Taiwan | Retrospective cohort study | 560,795 | Patients with incident T2DM diagnosis, diagnosed at least 3 times, age > 20 years old from all areas of taiwan | Expressed as Mean(SD) 1. No Frailty: 54.8 (13.2) 2. Pre-frail 1 Component: 60.8 (14.3) 3. Pre-frail 2 Component: 69.4 (12.7) 4. Frailty >= 3 components: 75.1 (11.2) | 1. No Frailty: 239499 (54.9%) 2. Pre-frail 1 Component: 54668 (50.7%) 3. Pre-frail 2 Component: 7470 (49.5%) 4. Frailty >= 3 components: 731 (51.6%) | not specified | -Secondary Data -Longitudinal Cohort of Diabetes Patients database (LHDB) | not specified | 1) Assess differential risk of diabetic related complications across groups 2) Assess differential healthcare utilization  3) Assess mortality | 1. Frailty | Judgemental splitting by patients' clinical characteristics | SAS | 4 | 1. No Frailty 2. Pre-frail 1 Component 3. Pre-frail 2 Component 4. Frailty >= 3 components |
| 19 | Chen HL et al (2015) | Changes in prevalence of diabetic complications and associated healthcare costs during a 10-year follow-up period among a nationwide diabetic cohort. | Asia | Taiwan | Retrospective cohort study | 136,372 | Patients age >= 18 years, diagnosed with diabetes mellitus between Jan 1 2000 and Dec 31 2011, and had at least one inpatient or two outpatient visits with a diagnosis of DM, with full medical and pharmacy coverage | Expressed in mean (SD) Total: 55.26 (13.80)  1. aDSCI scores 0: 52.30 (13.30)  2. aDSCI scores 1: 57.00 (12.95) 3. aDSCI scores 2: 60.42 (12.69)  4. aDSCI scores 3: 59.30 (14.35)  5. aDSCI scores 4: 62.69 (13.84)  6. aDSCI scores 5+: 62.95 (14.00) | Total: 68186 (50%)  1. aDSCI scores 0: 37488 (49%)  2. aDSCI scores 1: 13215 (48%)  3. aDSCI scores 2: 6049 (50%)  4. aDSCI scores 3: 5724 (55%)  5. aDSCI scores 4: 2516 (55%)  6. aDSCI scores 5: 2996 (57%) | Expressed in mean (SD) Total: 7.40 (1.99) 1. aDSCI scores 0: 7.43 (1.98) 2. aDSCI scores 1: 7.52 (1.96) 3. aDSCI scores 2: 7.48 (1.96) 4. aDSCI scores 3: 7.20 (2.03) 5. aDSCI scores 4: 7.11 (2.01) 6. aDSCI scores 5+: 6.91 (2.02) | -Secondary data -Taiwan's National Health Insurance Research Database (NHIRD) | not specified | 1) Assess differential risk of diabetic related complications across groups 2) Assess differential healthcare utilization | 1. Adapted Diabetes Complications Severity Index (aDCSI) score | Judgemental splitting by patients' clinical characteristics | SAS Version 9.3 | 6 | 1. aDSCI scores 0 2. aDSCI scores 1 3. aDSCI scores 2 4. aDSCI scores 3 5. aDSCI scores 4 6. aDSCI scores 5+ |
| 20 | Cheng XB et al (2012) | Obesity and low target attainment rates in Chinese with type 2 diabetes. | Asia | China | Cross-sectional study | 520 | Adult T2DM patients who were being followed up regularly in the outpatient department of the hospital for 6 months. | Expressed as Mean(SD) All: 58.2 (12.8)  1. <18.5: 62.3 (12.6)  2. 18.5–24: 58.9 (12.4) 3. 24–28: 56.3 (12.9) 4. >28: 55.8 (14.1) | All: 282 (54.2%) 1. <18.5: 24 (47.1%) 2. 18.5–24: 141 (53.4%) 3. 24–28: 97 (61.8%) 4. >28: 19 (41.3%) | not specified | Primary, interview and comprehensive clinical assessment | tertiary | Assess diabetic control across groups | 1. Body mass index (BMI) | Judgemental Splitting by patients' sociodemographic and/or economic attributes | SAS 9.1 | 4 | 1. BMI <18.5 2. BMI: 18.5–24 3. BMI: 24–28 4. BMI: >28 |
| 21 | Cheng Y et al (2014) | Cardiometabolic risk profiles associated with chronic complications in overweight and obese type 2 diabetes patients in South China. | Asia | China | Cross-sectional study | 2954 | T2DM patients with BMI >= 25kg/m2, aged >= 18, who visited diabetes clinics of 62 hospitals in 21 cities in Guangdong, China | Expressed as Mean(SD) All: 58.5 (13.1) 1. Men: 55.(13.9) 2. Women: 61.2 (11.6) | 1. Diabetic retinopathy [DR]: 1425 (50%) 2. Diabetic neuropathy [DN]: 1429 (50.0%) 3. Diabetic peripheral neuropathy [DPN]: 1426 (50.0%) 4. Cardiovascular disease [CVD]: 1426 (50.0%) 5. Stroke: 1418 (50.0%) | Expressed as Mean(SD) All: 7.0 (6.3) 1. Men: 6.1 (5.8) 2. Women: 7.8 (6.7) | -Primary data -Standard questionnaire, clinical examination and laboratory data | -Tertiary | 1) Health grouping/profiling 2) Assess differential risk of diabetic related complications across groups 3) Assess non-diabetes metabolic derangements (e.g. lipid, blood pressure) across groups | 1. Cardiometabolic complications | Judgemental splitting by patients' clinical characteristics | SPSS version 13.0 | 5 | 1. Diabetic retinopathy  2. Diabetic neuropathy  3. Diabetic peripheral neuropathy 4. Cardiovascular disease 5. Stroke |
| **S/No** | **Authors (Year)** | **Article Name** | **Continent** | **Country** | **Study design** | **Sample size** | **T2DM Patient population** | **Mean / Median Age of patients** | **Gender [Male (%)]** | **Duration of diabetes** | **Data source** | **Study setting** | **Objective of segmentation** | **Segmentation variables** | **Statistical methods used** | **Software** | **Number of segments** | **Categories of segments** |
| 22 | Cheong AT et al (2013) | Poor glycemic control in younger women attending Malaysian public primary care clinics: findings from adults diabetes control and management registry. | Asia | Malaysia | Cross-sectional study | 30,427 | Adult T2DM, Malaysian women aged 18 years old and above and with T2DM duration for at least 1 year | Expressed as Mean(SD) 1. Reproductive: 38.7 (6.2)  2. Non-reproductive: 55.5 (9.1) | N.A (all women participants) | **Expressed in Mean(SD) <5 years  1. Reproductive: 4115 (62.1)  2. Non-reproductive: 10325 (43.4)  5 – 10 years 1. Reproductive: 2103 (31.8)  2. Non-reproductive: 9600 (40.3)  >10 years  1. Reproductive: 404 (6.1)  2. Non-reproductive: 3880 (16.3)** | Secondary, data from the Adult Diabetes Control and Management (ADCM). | Primary | Assess diabetic control across groups | 1. Reproductive and non-reproductive age | Judgemental Splitting by patients' sociodemographic and/or economic attributes | PASW 18.0 | 2 | 1. Reproductive age group 2. Non-reproductive age group |
| 23 | Chew BH et al (2011) | Ethnic differences in glycaemic control and complications: the adult diabetes control and management (ADCM), Malaysia. | Asia | Malaysia | Cross-sectional study | 20330 | T2DM patients aged 18 and above from 54 primary care health centres | Expressed in Mean(SD)  57.9 (11.6) | 8826 (43.4%) | not specified | -Secondary. Data from the Audit of Diabetes Control and Management (ADCM), an ongoing patient registry focused on diabetes control and management | primary | - Assess diabetic control across groups  - Assess differential risk of diabetic related complications across groups  - Health Grouping/Profiling | 1. Patients' ethnicity | Judgemental Splitting by patients' sociodemographic and/or economic attributes | stata version 9 | 3 | 1. Malay 2. Chinese 3. Indian |
| **S/No** | **Authors (Year)** | **Article Name** | **Continent** | **Country** | **Study design** | **Sample size** | **T2DM Patient population** | **Mean / Median Age of patients** | **Gender [Male (%)]** | **Duration of diabetes** | **Data source** | **Study setting** | **Objective of segmentation** | **Segmentation variables** | **Statistical methods used** | **Software** | **Number of segments** | **Categories of segments** |
| 24 | Chew BH et al (2013) | Type 2 diabetes mellitus patient profiles, diseases control and complications at four public health facilities- A cross-sectional study based on the Adult Diabetes Control and Management (ADCM) registry 2009 | Asia | Malaysia | Cross-sectional study | 57780 | Adult T2DM patients (>=18 years old) from public health centres | Expressed as n(%) 1. Hospital with specialist (HS):  Mean 61.7 years old (SD: 12.8) <30 years old: 18 (1.1%) 30-49 years old : 233 (14.8%) 50-69 years old : 871 (55.4%) >=70 years old: 450 (28.6%)  2. Hospital without specialist (HNS):  Mean 58.9 years old (SD: 10.7) <30 years old: 5 (0.5%) 30-49 years old: 179 (17.3%)  50-69 years old : 664 (64.2%) >=70 years old: 186 (18.0%)  3. Health clinics with family medicine specialist (CS):  Mean 58.1 years old (SD: 11.0) <30 years old: 337 (0.8%) 30-49 years old: 8841 (20.5%)  50-69 years old: 27246 (63.2%) >=70 years old: 6676 (15.5%)  4. Health clinic without doctor (CND):  Mean 58.0 years old (SD: 10.9) <30 years old: 103 (1.1%) 30-49 years old: 2460 (19.9%) 50-69 years old: 7586 (57.9%)  >=70 years old: 2777 (21.2%) | Total: 23297 (40.3%) 1. Hospital with specialist (HS): 802 (51%) 2. Hospital without specialist (HNS): 427 (41.3%) 3. Health clinics with family medicine specialist (CS): 17524 (40.7%) 4. Health clinic without doctor (CND): 4544 (37.6%) | Expressed as n(%) 1. Hospital with specialist (HS):  Overall: Mean 11.1 years (SD: 8.7) <5 years: 409 (26.1%) 5-9 years: 387 (24.7%) >=10 years: 771 (49.2%)  2. Hospital without specialist (HNS):  Overall: Mean 6.4 years (SD: 5.72) <5 years: 483 (46.9%) 5-9 years: 347 (33.7%) >=10 years: 199 (19.3%)  3. Health clinics with family medicine specialist (CS):  Overall: Mean 5.9 years (SD: 5.53) <5 years: 21405 (49.9%) 5-9 years: 14116 (32.9%) >=10 years: 7335 (17.1%)  4. Health clinic without doctor (CND):  Overall: Mean 4.9 years (SD: 4.62) <5 years: 6887 (57.3%) 5-9 years: 3664 (30.5%) >=10 years: 1459 (12.1%) | - Secondary data  - Health administrative registry | Primary and tertiary | - Health grouping / profiling - Assess differential risk of diabetic related complications across groups - Assess diabetic control across groups | 1. Type of healthcare utilization | Judgemental splitting by patients' clinical characteristics | STATA 9, SPSS 19.0 | 4 | 1. Hospital with specialist (HS) 2. Hospital without specialist (HNS) 3. Health clinics with family medicine specialist (CS) 4. Health clinic without doctor (CND) |
| **S/No** | **Authors (Year)** | **Article Name** | **Continent** | **Country** | **Study design** | **Sample size** | **T2DM Patient population** | **Mean / Median Age of patients** | **Gender [Male (%)]** | **Duration of diabetes** | **Data source** | **Study setting** | **Objective of segmentation** | **Segmentation variables** | **Statistical methods used** | **Software** | **Number of segments** | **Categories of segments** |
| 25 | Coleman SM et al (2013) | Depression and death in diabetes; 10-year follow-up of all-cause and cause-specific mortality in a diabetic cohort. | North America | United States of America | Prospective cohort study | 4128 | T2DM patients from nine Group Health Cooperative (GHC) primary care clinics | Expressed as Mean(SD) Total: 63.4 (13.4) 1. No depression: 64.0 (13.2) 2. Minor depression: 64.2 (13.8) 3. Major depression: 59.4 (13.8) | Total: 2141 (51.9) 1. No depression: 1754 (53.5%) 2. Minor depression: 178 (51.9%) 3. Major depression: 204 (41.2%) | Expressed as Mean(SD) Total: 9.6 (9.4) 1. No depression: 9.5 (9.5) 2. Minor depression: 10.6 (9.8) 3. Major depression: 9.6 (8.3) | -Secondary -Data from Pathways epidemiologic study -Primary -Baseline surveys including patient data, medical record review and automated laboratory data were utilised | -primary care | - Assess mortality | 1. Severity of depression | Judgemental splitting by patients' clinical characteristics | STATA 12.0 | 3 | 1. No depression 2. Minor depression 3. Major depression |
| 26 | De Cosmo S et al (2014) | Kidney dysfunction and related cardiovascular risk factors among patients with type 2 diabetes. | Europe | Italy | Cross-sectional study | 120,903 | Adult T2DM patients aged 18 years or older with at least 1 outpatient measurement of serum creatinine and albuminuria in the index year | Expressed as Mean(SD) Whole sample: 66.6 (11.0)  1. Alb− and low eGFR− : 64.1 (10.7)  2. Alb− and low eGFR+ : 74.0 (8.0) 3. Alb+ and low eGFR− : 65.0 (10.8) 4. Alb+ and low eGFR+ : 73.4 (8.5) | Whole sample: 70 247 (58.1) 1. Alb− and low eGFR−: 35 822 (56.3)  2. Alb− and low eGFR+ : 5 776 (42.2)  3. Alb+ and low eGFR− : 19 874 (69.0) 4. Alb+ and low eGFR+ : 8741 (59.5) | Expressed as Mean(SD) Whole sample: 11.1 (9.4)  1. Alb− and low eGFR− : 9.7 (8.6)  2. Alb− and low eGFR+ : 13.3 (10.4)  3. Alb+ and low eGFR− : 11.0 (9.0) 4. Alb+ and low eGFR+: 15.3 (10.5) | Secondary. electronic medical records by means of an ad hoc-developed software. | primary | Assess differential risk of diabetic related complications across groups | 1. Presence of albuminuria and low eGFR | Judgemental splitting by patients' clinical characteristics | not specified | 4 | 1. Normoalbuminuria and normal eGFR 2. Normoalbuminuria and low eGFR 3. Albuminuria and normal eGFR 4. Albuminuria and low eGFR |
| 27 | de Rekeneire N et al (2003) | Racial differences in glycemic control in a well-functioning older diabetic population: findings from the Health, Aging and Body Composition Study. | North America | United States of America | Cross-sectional study | 468 | Adults T2DM patients aged 70 –79 years, who at baseline reported no difficulty walking one-quarter mile or up one flight of stairs without resting | Expressed in Mean(SD) Women: 1. Black: 73.4 (2.9)  2. White: 73.3 (3)   Men: 1. Black: 73.7 (2.7) 2. White: 73.9 (2.8) | Black: 120 (21.7%) White: 130 (13.8%) | not specified | primary, baseline home interview and  clinic-based examination | not specified | Assess diabetic control across groups | 1. Patients' race | Judgemental Splitting by patients' sociodemographic and/or economic attributes | SAS | 2 | 1. Black 2. White |
| **S/No** | **Authors (Year)** | **Article Name** | **Continent** | **Country** | **Study design** | **Sample size** | **T2DM Patient population** | **Mean / Median Age of patients** | **Gender [Male (%)]** | **Duration of diabetes** | **Data source** | **Study setting** | **Objective of segmentation** | **Segmentation variables** | **Statistical methods used** | **Software** | **Number of segments** | **Categories of segments** |
| 28 | de Vries McClintock HF et al (2016) | Patterns of Adherence to Oral Hypoglycemic Agents and Glucose Control among Primary Care Patients with Type 2 Diabetes. | North America | United States of America | Randomized controlled trial | 180 | Adult T2DM patients recruited from three primary care practices in Philadelphia, Pennsylvania. | Expressed as Mean(SD) 57.4 (9.5) | 58 (32.2%) | not specified | Primary. Electronic medical records, electronic monitoring data obtained from the Medication Event Monitoring System (MEMS) Caps, standardized questionnaire, Mini-Mental State Examination (MMSE) assessment, self-administered PHQ-9 | primary care | Assess treatment adherence - Assess diabetic control across groups | 1. Patterns of Adherence to Oral Hypoglycemic Agents | Growth Curve Mixture modelling | STATA version 12 for Windows, Mplus version 7 | 3 | 1. Adherent to oral hypoglycemia agents 2. Increasing adherence to oral hypoglycemia agents 3. Non-adherent to oral hypoglycemia agents |
| **S/No** | **Authors (Year)** | **Article Name** | **Continent** | **Country** | **Study design** | **Sample size** | **T2DM Patient population** | **Mean / Median Age of patients** | **Gender [Male (%)]** | **Duration of diabetes** | **Data source** | **Study setting** | **Objective of segmentation** | **Segmentation variables** | **Statistical methods used** | **Software** | **Number of segments** | **Categories of segments** |
| 29 | Demmer RT et al (2015) | Sex Differences in the Association Between Depression, Anxiety, and Type 2 Diabetes Mellitus | North America | United States of America | Prospective cohort study | 3233 | Adult T2DM patients aged 25–74 years old | Expressed in Mean(SD) Anxiety symptoms 1. Men (Low anxiety symptomatology): 52 (0.5) 2. Men (Moderate anxiety symptomatology): 48 (0.6) 3. Men (High anxiety symptomatology): 48 (1.0) 4. Women (Low anxiety symptomatology): 51 (0.5) 5. Women (Moderate anxiety symptomatology): 48 (0.6) 6. Women (High anxiety symptomatology): 49 (0.8)   Depression symptoms 1. Men (Low anxiety symptomatology): 50 (0.4) 2. Men (Moderate anxiety symptomatology): 50 (0.6) 3. Men (High anxiety symptomatology): 53 (1.3) 4. Women (Low anxiety symptomatology): 50 (0.5) 5. Women (Moderate anxiety symptomatology): 49 (0.6) 6. Women (High anxiety symptomatology): 48 (0.1) | 1544 (47.8%) | Not specified | Secondary source  Patient databases from NHANES and Detroit Neighborhood Study | Primary | Assess psychological symptoms across groups | 1. Anxiety symptomatology level 2. Depression symptomatology level | Judgemental splitting by patients' clinical characteristics | SAS version 9.3 SAS-callable SUDAAN version 10 | Anxiety symptoms: 6 Depressive symptoms: 6 | Anxiety symptoms  1. Men (Low anxiety symptomatology) 2. Men (Moderate anxiety symptomatology)  3. Men (High anxiety symptomatology)  4. Women (Low anxiety symptomatology)  5. Women (Moderate anxiety symptomatology)  6. Women (High anxiety symptomatology)   Depression symptoms  1. Men (Low anxiety symptomatology) 2. Men (Moderate anxiety symptomatology)  3. Men (High anxiety symptomatology)  4. Women (Low anxiety symptomatology)  5. Women (Moderate anxiety symptomatology)  6. Women (High anxiety symptomatology) |
| **S/No** | **Authors (Year)** | **Article Name** | **Continent** | **Country** | **Study design** | **Sample size** | **T2DM Patient population** | **Mean / Median Age of patients** | **Gender [Male (%)]** | **Duration of diabetes** | **Data source** | **Study setting** | **Objective of segmentation** | **Segmentation variables** | **Statistical methods used** | **Software** | **Number of segments** | **Categories of segments** |
| 30 | Duan JG et al (2015) | Sex differences in epidemiology and risk factors of acute coronary syndrome in Chinese patients with type 2 diabetes: a long-term prospective cohort study. | Asia | Hong Kong | Prospective cohort study | 2105 | Adult Hong Kong Chinese with T2DM without history of stroke, transient ischemic attack or ischemic heart disease. | Expressed in Mean(SD) All: 55.18 (11.31) 1. Men: 54.61 (11.24)  2. Women: 55.59 (11.35) | 876 (41%) | Expressed in Mean(SD) All: 80.71 (72.79) 1. Men: 71.80 (67.86) 2. Women: 86.90 (75.45) | primary. Questionnaire, clinical data from reviewing medical records | tertiary | Assess non-diabetes metabolic derangements (e.g. lipid, blood pressure) across groups | 1. Patient's gender | Judgemental Splitting by patients' sociodemographic and/or economic attributes | PASW Statistics (version 18.0) | 2 | 1. Men 2. Women |
| 31 | Egede LE et al (2015) | Differential impact of mental health multimorbidity on healthcare costs in diabetes. | North America | United States of America | Retrospective cohort study | 733071 | Veterans who had:  1) DM defined by 2 or more International Classification of Diseases, Ninth Revision, Clinical Modification (ICD-9-CM) codes for DM in the previous 24 months (in 2000 and 2001) 2) ICD-9-CM codes for DM from inpatient stays and/or outpatient visits on separate days (excluding codes from lab tests and other nonclinician visits) in 2002 3) prescriptions for insulin or oral hypoglycemic agents in 2002 based on a previously validated algorithm | 1. All: 65.7 (11.1)  2. MHC = 0: 66.6 (10.8)  3. MHC = 1: 62.1 (11.4)  4. MHC = 2: 57.1 (10.6)  5. MHC = 3: 52.6 (8.0) | 1. All: 716,943 (97.8%) 2. MHC = 0: 599,975 (98.0%) 3. MHC = 1: 95,142 (96.4%)  4. MHC = 2: 17,690 (96.0%)  5. MHC = 3: 3,622 (97.1%) | not specified | -secondary -patient and administrative files from the VHA National Patient Care (NPC) and Pharmacy Benefits Management (PBM) databases | -tertiary | - Assess differential healthcare utilization | 1. Number of mental health comorbidities | Judgemental splitting by patients' clinical characteristics | SAS Proc GLIMMIX | 4 | 1. Number of mental health comorbidities = 0 2. Number of mental health comorbidities = 1 3. Number of mental health comorbidities = 2  4. Number of mental health comorbidities = 3 |
| **S/No** | **Authors (Year)** | **Article Name** | **Continent** | **Country** | **Study design** | **Sample size** | **T2DM Patient population** | **Mean / Median Age of patients** | **Gender [Male (%)]** | **Duration of diabetes** | **Data source** | **Study setting** | **Objective of segmentation** | **Segmentation variables** | **Statistical methods used** | **Software** | **Number of segments** | **Categories of segments** |
| 32 | Elder DH et al (2016) | Mean HbA1c and mortality in diabetic individuals with heart failure: a population cohort study. | Europe | Scotland | Retrospective cohort study | 1447 | Adult T2DM patients who develop incident CHF and had to have at least one HbA1c measurement recorded after CHF diagnosis | Expressed as Mean(SD) All: 71.8(9.95)  <6: 73.5 (9.52)  6.1 - 7.0: 73.6 (9.84)  7.1 - 8.0: 72 (9.49)  8.1 - 9.0: 70.6 (9.69)  >9: 68.1 (10.5) | All: 896 (61.9%)  <6: 107 (62.6%) 6.1 - 7.0: 269 (65.5%) 7.1 - 8.0: 100 (59.3%)  8.1 - 9.0: 141 (59.5%)  >9: 133 (62.4%) | Expressed as Median (IQR) All:7.7 (3.5, 13.2) <6: 5.4 (2.4, 10.6) 6.1 - 7.0: 5.7 (2.35, 10.6) 7.1 - 8.0: 8.1 (3.72, 14)  8.1 - 9.0: 10.1 (5.9, 15.6) >9: 9.9 (5.4, 15.6) | secondary. Regional clinical informatics systems developed in partnership between the University of Dundee and NHS Tayside which makes use of a unique health record identifier, the Community Health Index (CHI) number, to link multiple clinical data sets deterministically through established and robust nonymization protocols within the Health Informatics Centre (HIC) and the University of Dundee at an individual level with high accuracy. | tertiary | Assess mortality | 1. HbA1c level | Judgemental splitting by patients' clinical characteristics | R version 3.2.0 | 5 | 1. Hbac <6% 2. Hba1c: 6.1–7.0% 3. Hba1c: 7.1–8.0% 4. Hba1c: 8.1–9.0% 5. Hba1c: >9% |
| **S/No** | **Authors (Year)** | **Article Name** | **Continent** | **Country** | **Study design** | **Sample size** | **T2DM Patient population** | **Mean / Median Age of patients** | **Gender [Male (%)]** | **Duration of diabetes** | **Data source** | **Study setting** | **Objective of segmentation** | **Segmentation variables** | **Statistical methods used** | **Software** | **Number of segments** | **Categories of segments** |
| 33 | Elissen AMJ et al (2017) | Differences in biopsychosocial profiles of diabetes patients by level of glycaemic control and health-related quality of life: The Maastricht Study. | Europe | Netherlands | Cross-sectional study | 840 | T2DM age between 40 and 75 years, and living in the Maastricht region | Health related quality of life (HrQOL) Expressed as Freqencies and Valid Percentages 1. High HRQoL Class:  41-49: 20 (9.1%) 50-64: 108 (49.1%) 65-76: 92 (41.8%) 2. Low HRQoL Class: 3 (3.4) 41-49: 3 (3.4%) 50-64: 41 (46.6%) 65-76: 44 (50.0%) Level of glycaemic control (HbA1C) Expressed as Mean(SD) 1. HbA1c <= 7.0: 62.9 (7.6)  2. HbA1c > 7.0: 62.3 (7.7) | Health related quality of life (HrQOL) 1. High HRQoL Class: 167 (75.9%) 2. Low HRQoL Class: 51 (58.0%) Level of glycaemic control (HbA1C) 1. HbA1c <= 7.0: 358 (67.3%)  2. HbA1c > 7.0: 218 (70.8%) | Health related quality of life (HrQOL) Expressed as Frequency and valid percentages 1. High HRQoL Class:  < 5 years: 52 (28.9%) 5 - 9 years: 42 (23.3%) >= 10 years: 86 (47.8%) 2. Low HRQoL Class:  < 5 years: 11 (15.5%) 5 - 9 years: 18 (25.4%) >= 10 years: 42 (59.2%) Level of glycaemic control (HbA1C) Expressed as Mean (SD) 1. HbA1c <= 7.0: 6.88 (5.89) 2. HbA1c > 7.0: 11.13 (7.96) | - Secondary - data from The Maastricht Study | not specified | - Health Grouping/Profiling - Assess diabetic control across groups  - Assess differential QoL measures across groups | Health related quality of life (HrQOL) 1. EQ-5D scores 2. SF-36 scores Level of glycaemic control (HbA1C) 1. HbA1c <= 7.0 2. HbA1c > 7.0 | Latent class analysis | IBM SPSS Statistics for Windows, version 23.0 | 2 | 1. High HRQoL 2. Low HRQoL |
| 34 | El-Kebbi IM et al (2003) | Association of Younger Age With Poor Glycemic Control and Obesity in Urban African Americans With Type 2 Diabetes | North America | United States of America | Retrospective cohort study | 2539 | Adult T2DM patients with an initial visit between April 1, 1991, and December 31, 1998, and with HbA1c levels measured at the time of the intake visit and at follow-up 5 to 12 months later. | Expressed as Mean(SD) All: 55 (12) 1. <30 years old: 25 (4) 2. 30 - 49 years old: 43 (5) 3. 50 - 69 years old: 59(5) 4. >69 years old: 76 (4) | All: 25 (34%) 1. <30 years old: 17 (27%) 2. 30 - 49 years old: 298 (38%) 3. 50 - 69 years old: 474 (33%) 4. >69 years old: 74 (29%) | Expressed as Mean(SD) All: 5.2 (7.9) 1. <30 years old: 2.3 (3.2) 2. 30 - 49 years old: 3.0 (5.2) 3. 50 - 69 years old: 6.3 (8.2) 4. >69 years old: 6.4 (8.8) | Primary data Clinic health records | Primary | Assess diabetic control across groups | 1. Patients' age | Judgemental Splitting by patients' sociodemographic and/or economic attributes | SPSS version 9.0 software (SPSS Inc, Chicago, Ill) | 4 | 1. <30 years old 2. 30 - 49 years old 3. 50 - 69 years old 4. >69 years old |
| 35 | Emanuele N et al (2005) | Ethnicity, race, and baseline retinopathy correlates in the veterans affairs diabetes trial. | North America | United States of America | Cross-sectional study | 1283 | Veterans with T2DM from 20 medical centres | (Not specified whether mean or median) 1. Non-Hispanic white: 61 2. Hispanic: 58 3. African American: 58  4. Other: 59 | 1245 (97%) | 1. Non-Hispanic white: 11 2. Hispanic: 11 3. African American: 11  4. Other: 12 | primary Seven-field standard stereoscopic color photographs of both eyes, patient self-reported race/ethnicity, Cardiovascular history by patient recall and supplemented by review of available VA records. | not specified | Assess differential risk of diabetic related complications across groups | 1. Patients' race and ethnicity | Judgemental splitting by patients' clinical characteristics | not specified | 4 | 1. Non-Hispanic White 2. Hispanic 3. African American 4. Others |
| **S/No** | **Authors (Year)** | **Article Name** | **Continent** | **Country** | **Study design** | **Sample size** | **T2DM Patient population** | **Mean / Median Age of patients** | **Gender [Male (%)]** | **Duration of diabetes** | **Data source** | **Study setting** | **Objective of segmentation** | **Segmentation variables** | **Statistical methods used** | **Software** | **Number of segments** | **Categories of segments** |
| 36 | Ernande L et al (2017) | Clinical Implications of Echocardiographic Phenotypes of Patients With Diabetes Mellitus | Australia and Europe | Australia, France | Prospective cohort study | 842 | T2DM patients with normal left ventricular ejection fraction (LVEF >50%) and taking oral hypoglycemic or insulin treatment | Expressed as Mean (SD) 1. Cluster 1: 55.7 (9.0) 2. Cluster 2: 60.0 (8.9) 3. Cluster 3: 56.3 (8.3) | 1. Cluster 1: 176 (55.2%) 2. Cluster 2: 125 (47.0%) 3. Cluster 3: 122 (76.2%) | Expressed as Mean (SD) 1. Cluster 1: 9.6 (7.6) 2. Cluster 2: 9.2 (7.6) 3. Cluster 3: 10.0 (7.5) | -Primary -clinical data, echocardiography, biochemical analysis and physical examination | tertiary | - Health Grouping/Profiling  - Assess differential risk of diabetic related complications across groups | 1. Echocardiographic variables 2. Presence of hypertension 3. Obesity | Cluster analysis | STATA 14.1 | 3 | 1. Cluster 1: Lowest LVMi and E/e′ ratio values, the highest LVEF, and the second highest strain values. Predominantly male patients, with the lowest rate of obesity or HTN.  2. Cluster 2: Highest strain values but the lowest e′ velocities and the highest E/e′ ratio. Comprised of the oldest patients and was predominantly female with the lowest rate of isolated T2DM. Blood pressure levels, BMI, and heart rate were the highest  3. Cluster 3: Highest LVMi and LV volumes and the lowest LVEF and strain. This cluster comprised predominantly males, with similar age and rate of obesity and HTN as cluster 1  Abbreviations: e': mitral annular early diastolic velocity at septaland lateral sites of the mitral annulus; E: Peak mitral early diastolic velocities; LVMi: Left ventricular mass indexed to body surface area; |
| **S/No** | **Authors (Year)** | **Article Name** | **Continent** | **Country** | **Study design** | **Sample size** | **T2DM Patient population** | **Mean / Median Age of patients** | **Gender [Male (%)]** | **Duration of diabetes** | **Data source** | **Study setting** | **Objective of segmentation** | **Segmentation variables** | **Statistical methods used** | **Software** | **Number of segments** | **Categories of segments** |
| 37 | Escalada J et al (2014) | Outcomes and healthcare resource utilization associated with medically attended hypoglycemia in older patients with type 2 diabetes initiating basal insulin in a US managed care setting. | North America | United States of America | Retrospective cohort study | 31,035 | Adult T2DM who previously used OADs/ GLP-1 analogs, who had initiated insulin treatment on a basal insulin only regimen, and had at least 2 years of continuous Medicare Advantage coverage | Expressed as Mean(SD) 1. HG group: 73 (9.6)  2. Non-HG group: 72 (9.1) | 1. HG group: 1410 (46%) 2. Non-HG group: 13425 (48%) | not specified | Secondary Medicare Advantage claims database | not specified | - Assess differential healthcare utilization  - Assess treatment outcomes | 1. Medically attended hypoglycemia (HG) (Yes / No) | Judgemental splitting by patients' clinical characteristics | not specified | 2 | 1. Patients with medically attended hypoglycemia during the first year of BI treatment (HG group)  2. Patients without medically attended hypoglycemia during the first year of BI treatment (HG group) |
| 38 | Escobar C et al (2011) | Prevalence and clinical profile and management of peripheral arterial disease in elderly patients with diabetes. | Europe | Spain | Cross-sectional study | 1462 | Adult T2DM patients of both sexes, > 70 years old, and with an established diagnosis of diabetes mellitus | Expressed in Mean(SD) Overall: 78.05 (5.62)  1. Vascular surgery: 77.07 (5.15) 2. Medical specialties: 79.30 (5.92) | Overall: 864 (59.1%) 1. Vascular surgery: 559 (68.4%) 2. Medical specialties: 305 (47.3%) | not specified | Primary. bio-demographic data, risk factors, history of cardiovascular disease and treatments, were recorded, complete physical examination, including ABI, as well as the Edinburgh Claudication Questionnaire was performed | tertiary | Health Grouping/Profiling - Assess differential risk of diabetic related complications across groups | 1. Medical specialty patients receive care from | Judgemental splitting by patients' clinical characteristics | SPSS version 15. | 2 | 1. Vascular surgery 2. Medical specialties |
| 39 | Ezenwaka CE et al (2002) | Differences in Cardiovascular Disease Risk Factors in Elderly and Younger Patients with Type 2 Diabetes in the West Indies | South America | Trinidad | Cross-sectional study | 191 | Adult T2DM patients visiting outpatient clinics at Arimaand Chaguanas | Expressed in Mean(SD) 1. Men<60 years old: 49.4 (1.4) 2. Women <60 years old: 48.4 (0.8) 3. Men >60 years old: 69.5 (1.1) 4. Women >60 years old: 66.8 (0.8) | 1. Patients <60 years old: 112 (58.6%) 2. Patients >60 years old: 79 (41.4%) | Expressed as Mean(SD) Patients <60 years old: 7.6 +/- 0.7 1. Men <60 years old: 9.2 +/- 1.5 2. Women <60 years old: 7.0 +/- 6.4  Patients >60 years old: 11.3 +/- 1.0 3. Men >60 years old: 12.5 +/- 1.8 4. Women >60 years old: 10.6 +/- 1.3 | Primary  Questionnaires and blood tests | Primary | Assess non-diabetes metabolic derangements (e.g. lipid, blood pressure) across groups Assess diabetic control across groups | 1. Patients' age 2. Patients' gender | Judgemental Splitting by patients' sociodemographic and/or economic attributes | SPSS | 4 | 1. Age <60 years old, Men 2. Age <60 years old, Women 3. Age >60 years old, Men 4. Age >60 years old, Women |
| **S/No** | **Authors (Year)** | **Article Name** | **Continent** | **Country** | **Study design** | **Sample size** | **T2DM Patient population** | **Mean / Median Age of patients** | **Gender [Male (%)]** | **Duration of diabetes** | **Data source** | **Study setting** | **Objective of segmentation** | **Segmentation variables** | **Statistical methods used** | **Software** | **Number of segments** | **Categories of segments** |
| 40 | Fink JT et al (2018) | Blood Pressure Control and Other Quality of Care Metrics for Patients with Obesity and Diabetes: A Population-Based Cohort Study. | North America | United States of America | Retrospective cohort study | 51,229 | Adult patients age 18-75 with diabetes who receive primary care in ambulatory practices within one of the three health systems with two years of electronic health record data | Expressed as mean (SD) All: 58 (11) 1. Normal (18.4–24.9 kg/m2): 57 (14) 2. Overweight (25–29.9 kg/m2): 59 (11) 3. Obese class I (30–34.9 kg/m2): 59 (10) 4. Obese class II (35–39.9 kg/m2): 58 (11) 5. Obese class III (≥ 40 kg/m2): 56 (11) | All: 26127 (51%) 1. Normal (18.4–24.9 kg/m2): 1729 (50%) 2. Overweight (25–29.9 kg/m2): 4464 (58%) 3. Obese class I (30–34.9 kg/m2): 7998 (57%) 4. Obese class II (35–39.9 kg/m2): 5489 (50) 5. Obese class III (≥ 40 kg/m2): 4854 (40) | Not specified | -Secondary Data -Electronic health record data for three health systems that participate in the Wisconsin Collaborative for Healthcare Quality (WCHQ) | Primary | 1) Assess diabetic control across groups 2) Assess non-diabetes metabolic derangements (e.g. lipid, blood pressure) across groups | 1. By extent of obesity using body mass index (BMI) | Judgemental splitting by patients' clinical characteristics | Stata 13.1 | 5 | 1. Normal (18.4–24.9 kg/m2) 2. Overweight (25–29.9 kg/m2) 3. Obese class I (30–34.9 kg/m2) 4. Obese class II (35–39.9 kg/m2) 5. Obese class III (≥ 40 kg/m2) |
| 41 | Franch-Nadal J et al (2014) | Metabolic control and cardiovascular risk factors in type 2 diabetes mellitus patients according to diabetes duration. | Europe | Spain | Cross-sectional study | 3130 | Adult T2DM patients seen in primary care centres during 2007 | Expressed as Mean(SD) Total: 68.0 (11.7) 1. 0-5 years: 66.1 (12.2) 2. 6-10 years: 68.9 (10.9)  3. 11-20 years: 71.5 (10.3)  4. >/= 20 years: 74.6 (9.2) | Total: 1613 (51.5%) 1. 0-5 years: 874 (53.3%)  2. 6-10 years: 475 (51.4%)  3. 11-20 years: 233 (47.6%) 4. >/= 20 years: 29 (34.9%) | Expressed as N(%) 1. 0-5 years: 1634 (52.2%) 2. 6-10 years: 924 (29.5%)  3. 11-20 years: 489 (15.6%) 4. >/= 20 years: 83 (2.65%) | secondary, electronic medical records by physicians participating in the GEDAPS Continuous Quality Improvement (GCQI) program in Catalonia | primary | Assess non-diabetes metabolic derangements (e.g. lipid, blood pressure) across groups | 1. Duration of T2DM | Judgemental splitting by patients' clinical characteristics | not specified | 4 | 1. Duration of T2DM: 0 - 5 years 2. Duration of T2DM: 6 - 10 years 3. Duration of T2DM: 11 - 20 years 4. Duration of T2DM: >20 years |
| 42 | Gao F et al (2017) | Latent class analysis suggests four classes of persons with type 2 diabetes mellitus based on complications and comorbidities in Tianjin, China: a cross-sectional analysis. | Asia | China | Cross-sectional study | 5,500 | Adult T2DM patients who were covered by special disease outpatient service insurance (SDOSI), were mentally competent and able to communicate verbally, and able to provide informed consent. | Expressed in Median (IQR) Overall: 63(14) 1. Class 1: 65(10)  2. Class 2: 64 (13) 3. Class 3: 65(12)  4. Class 4: 62(14) | Overall: 2688 (50.8%) 1. Class 1: 150 (46.4%) 2. Class 2: 692 (50.9%) 3. Class 3: 355 (46.9%) 4. Class 4: 1483 (52.0%) | Expressed in Median (IQR)  1. Class 1: 12(12) 2. Class 2: 10(9) 3. Class 3: 9(10)  4. Class 4: 7(7) | Primary, structured questionnaire | Tertiary | Health Grouping/Profiling | 1. Complications of T2DM  2. Comorbidities of patients | Latent class analysis | Mplus version 7, SAS version 9.4 | 4 | 1. Class 1: Complications & comorbidities group  2. Class 2: High risk of complications group  3. Class 3: High risk of comorbidities and CVD group  4. Class 4: Diabetes without complications and comorbidities group |
| **S/No** | **Authors (Year)** | **Article Name** | **Continent** | **Country** | **Study design** | **Sample size** | **T2DM Patient population** | **Mean / Median Age of patients** | **Gender [Male (%)]** | **Duration of diabetes** | **Data source** | **Study setting** | **Objective of segmentation** | **Segmentation variables** | **Statistical methods used** | **Software** | **Number of segments** | **Categories of segments** |
| 43 | Gao Y et al (2016) | The prevalence of mild cognitive impairment with type 2 diabetes mellitus among elderly people in China: A cross-sectional study | Asia | China | Cross-sectional study | 8213 | Adult T2DM patients aged 65 years and older in 2010 and self designated Han Ethnicity | Expressed as Mean(SD) Total: 72.4 (3.5)  1. MCI: 74.3 (3.5) 2. Demented: 77.4 (4.5)  3. Cognitive intactly: 70.9 (4.7) | Total: 485 (43.7%) 1. MCI: 300 (43.5%)  2. Demented: 57 (43.2%)  3. Cognitive intactly: 128 (44.6%) | Expressed as Mean(SD) Total: 11.2 (3.7)  1. MCI: 12.3 (3.7) 2. Demented: 14.5 (4.9) 3. Cognitive intactly: 10.9 (4.3) | primary. in-person interview of general health and function at the time of study entry followed by a standard assessment, including medical history, physical and neurological examination, and a neuropsychological battery. | not specified | Health Grouping/Profiling | 1. Cognitive status | Judgemental splitting by patients' clinical characteristics | SPSS13.0 software  package | 3 | 1. Mild cognitive impairment (MCI)  2. Demented  3. Cognitive intactly |
| 44 | Gariepy G et al (2011) | Types of smokers in a community sample of individuals with Type 2 diabetes: a latent class analysis. | North America | Canada | Cross-sectional study | 383 | Adults with self-reported T2DM who were current or recurrent smoker at baseline | Expressed as Mean(SD) 1. Class 1: 67.6 (5.8) 2. Class 2: 53.6 (10) 3. Class 3: 50.6 (8.4) | 1. Class 1: 55 (52%) 2. Class 2: 41 (39%) 3. Class 3: 80 (46%) | 1. Class 1 0 - 4.9 years: 17.6% 5 - 9.9 years: 22.7% >= 10 years: 59.7%  2. Class 2 0 - 4.9 years: 36.4% 5 - 9.9 years: 19.7% >= 10 years: 44.0%  3. Class 3 0 - 4.9 years: 51.8% 5 - 9.9 years: 29.3% >= 10 years: 18.9% | - Primary data - Health survey / questionnaire | Not specified | 1) Health Grouping/Profiling Identify distinguishing profiles of smokers in T2DM patients based on their smoking habits, socio-demographic status and health characteristics and the effects on outcomes of depression and disability | 1. Sociodemographic factors - Education, marital status and working status 2. Smoking duration 3. Lifestyle factors - alcohol consumption, physical activity level and weight status | Latent class analysis | SAS version 9.2 | 3 | 1. Class 1: Long-time smokers with long-standing diabetes 2. Class 2: Heavy smokers with deprived socio-economic status, poor health and unhealthy lifestyle characteristics 3. Class 3: Working and active smokers who were more recently diagnosed with diabetes |
| 45 | Genovese S et al (2006) | Clinical phenotype and beta-cell autoimmunity in Italian patients with adult-onset diabetes. | Europe | Italy | Cross-sectional study | 881 | T2DM patients recruited from five outpatient clinics located in different cities, diagnosed at age 40 years or above and age at recruitment >= 70 years | Expressed as Mean(SD) 1. Antibody positive: 60.76 (6.89) 2. Antibody negative: 60.12 (6.89) | 1. Antibody positive: 23 (36.5%) 2. Antibody negative: 438 (53.6%) | Expressed as Mean(SD) 1. Antibody positive: 8.74 (6.68) 2. Antibody negative: 29.20 (5.00) | -primary -Data from patients attending five outpatient clinics located in all italian geographical macro areas representing the north, centre, south and the islands, except Sardinia | tertiary | - Health Grouping/Profiling | 1. Presence of islet cell autoimmune antibodies | Judgemental splitting by patients' clinical characteristics | Strata 6 | 2 | 1. Islet cell antibody positive  2. Islet cell antibody negative |
| **S/No** | **Authors (Year)** | **Article Name** | **Continent** | **Country** | **Study design** | **Sample size** | **T2DM Patient population** | **Mean / Median Age of patients** | **Gender [Male (%)]** | **Duration of diabetes** | **Data source** | **Study setting** | **Objective of segmentation** | **Segmentation variables** | **Statistical methods used** | **Software** | **Number of segments** | **Categories of segments** |
| 46 | Ghane BM et al (2015) | Association of Major Dietary Patterns with General and Abdominal Obesity in Iranian Patients with Type 2 Diabetes Mellitus. | Asia | Iran | Cross-sectional study | 728 | Adult T2DM patients aged 35-65 years old | Expressed as Mean(SD) Quintiles of healthy dietary pattern Q1: 53.1 (6.9) Q3: 54.1 (6.6) Q5: 53.7 (6.5)   Quintiles of unhealthy dietary pattern Q1: 55.9 (6.1) Q3: 53.8 (6.0) Q5: 52.3 (6.8) | Not specified | Quintiles of healthy dietary pattern Quintile 1 <1 years: 5.4% 1-4 years: 36.7% 5-10 years: 29.3% >10 years: 28.6% Quintile 3 <1 years: 9.0% 1-4 years: 37.2% 5-10 years: 26.9% >10 years: 26.9% Quintile 5 <1 years: 5.5% 1-4 years: 42.1% 5-10 years: 26.2% >10 years: 26.2%  Quintiles of unhealthy dietary pattern Quintile 1 <1 years: 4.1% 1-4 years: 28.3% 5-10 years: 35.2% >10 years: 32.4% Quintile 3 <1 years: 6.9% 1-4 years: 38.2% 5-10 years: 29.9% >10 years: 25.0% Quintile 5 <1 years: 9.0% 1-4 years: 46.2% 5-10 years: 26.2% >10 years: 18.6% | Primary  Interview, clinical records, blood test | Tertiary | Health Grouping/Profiling Assess obesity rates across groups | 1. Dietary patterns of patients | Judgemental Splitting by patients' lifestyle habits | SPSS Version 21 | 1) Quintiles of healthy dietary patterns: 5 2) Quintiles of unhealthy dietary patterns: 5 | Quintiles of healthy dietary patterns 1. Quintile 1 2. Quintile 2 3. Quintile 3 4. Quintile 4 5. Quintile 5  Quintiles of unhealthy dietary patterns 1. Quintile 1 2. Quintile 2 3. Quintile 3 4. Quintile 4 5. Quintile 5 |
| 47 | Göbl CS et al (2010) | Sex-specific differences in glycemic control and cardiovascular risk factors in older patients with insulin-treated type 2 diabetes mellitus. | Europe | Austria | Cross-sectional study | 392 | Older T2DM patients aged ≥60 years with insulin-treated T2DM | Expressed in Mean(SD) 1. Women: 70.9 (7.66)  2. Men: 69.56 (6.64) | 209 (53.3%) | Expressed in Mean(SD) 1. Women: 18.94 (10.09)  2. Men: 17.16 (8.86) | Secondary. medical records as well as the clinic’s electronic medical information system and a 2006 prospectively compiled name register. | tertiary | - Assess differential risk of diabetic related complications across groups  - Assess non-diabetes metabolic derangements (e.g. lipid, blood pressure) across groups - Assess diabetic control across groups | 1. Patient's gender | Judgemental Splitting by patients' sociodemographic and/or economic attributes | R 2.9.0 , SPSS 13.0 | 2 | 1. Women 2. Men |
| 48 | Grégoire JP et al (2010) | Persistence patterns with oral antidiabetes drug treatment in newly treated patients--a population-based study. | North America | Canada | Retrospective cohort study | 98,940 | Adult T2DM patients aged 18 years or more newly dispensed an oral anti-diabetic agent between 1998 and 2003 | Expressed as Mean(SD) Age at treatment initiation Total: 64.8 (13.0) 1. Metformin: 64.2 (13.1)  2. Insulin secretagogues sulfonylureas: 66.1 (12.9) | Total: 48036 (48.5) 1. Metformin: 31944 (47.1) 2. Insulin secretagogues sulfonylureas: 16092 (51.6) | not specified | Secondary, databases of the Quebec Health Insurance Board (RAMQ) and the Quebec Registry of Hospitalizations. | not specified | Assess treatment adherence | 1. Initial oral antidiabetes drug (OAD) | Judgemental splitting by patients' clinical characteristics | SAS, version 9.1 | 2 | 1. Metformin group 2. Insulin secretagogues sulfonylureas group |
| **S/No** | **Authors (Year)** | **Article Name** | **Continent** | **Country** | **Study design** | **Sample size** | **T2DM Patient population** | **Mean / Median Age of patients** | **Gender [Male (%)]** | **Duration of diabetes** | **Data source** | **Study setting** | **Objective of segmentation** | **Segmentation variables** | **Statistical methods used** | **Software** | **Number of segments** | **Categories of segments** |
| 49 | Grenier J et al (2018) | Blood Pressure Management in Adults With Type 2 Diabetes: Insights From the Diabetes Mellitus Status in Canada (DM-SCAN) Survey. | North America | Canada | Cross-sectional study | 5172 | T2DM Patients cared for by primary care providers in Canada | Expressed as median (25th percentile, 75th percentile) 1. BP target not achieved (sBP >130mmHg):  64 (56, 72) 2. BP Target achieved (sBP <= 130mmHg):  64 (55, 73) | 1. BP target not achieved (sBP >130mmHg): 1768 (54.0%) 2. BP Target achieved (sBP <= 130mmHg): 1005 (53.7%) | Expressed as median (25th percentile, 75th percentile) 1. BP target not achieved (sBP >130mmHg):  8 (4, 12) 2. BP Target achieved (sBP <= 130mmHg):  8 (4, 12) | -Secondary Data -The Diabetes Mellitus Status in Canada (DM-SCAN) survey | Primary | 1) Health Grouping/Profiling  2) Assess differential risk of diabetic related complications across groups  3) Assess non-diabetes metabolic derangements (e.g. lipid, blood pressure) across groups  4) Assess diabetic control across groups | 1. Blood pressure control | Judgemental splitting by patients' clinical characteristics | SPSS ver 22 | 2 | 1. BP target not achieved (sBP >130mmHg) 2. BP Target achieved (sBP <= 130mmHg) |
| 50 | Griffiths RI et al (2012) | Epidemiology and outcomes of previously undiagnosed diabetes in older women with breast cancer: an observational cohort study based on SEER-Medicare. | North America | United States of America | Retrospective cohort study | 2418 | T2DM patients at least 67 years old, diagnosed with breast cancer between 01/01/2001 and 31/12/2005, breast being the first and only type of cancer at the time they were diagnosed, and diagnosed with diabetes between 24 months before and 3 months after cancer diagnosis, with at least 24 months of medicare coverage | Diabetes status Expressed as Mean(SD) Total: 77.8 (6.9) 1. Previously Diagnosed: 77.8 (7.0) 2. Previously undiagnosed: 77.7 (6.9) | 0 | NA. Participants were included if diagnosed with diabetes between 24 months before and 3 months after cancer diagnosis. | -secondary. SEER-Medicare (Surveillance, Epidemiology, and End Results (SEER) | tertiary | - Health Grouping/Profiling  - Assess differential risk of diabetic related complications across groups | 1. Diabetes status at cancer diagnosis | Judgemental splitting by patients' clinical characteristics | not specified | 2 | 1. Previously diagnosed diabetes at cancer diagnosis 2. Previously undiagnosed diabetes at cancer diagnosis |
| 51 | Gucciardi E et al (2011) | Profiles of smokers and non-smokers with type 2 diabetes: initial visit at a diabetes education centers. | North America | United States of America | Cross-sectional study | 275 | Adult T2DM patients aged > 18 years old attending two diabetes education centers (DEC), new to the center or re-referred to the center after a two-year period, responsible for self-managing their diabetes | Expressed as Mean(SD) Overall: 54.5 (11.8)  1. Smokers: 51.5 (13.3)  2. Non-smokers: 55.1 (11.5) | Overall: 132 (48%) 1. Smokers: 22 (55%) 2. Non-smokers: 110 (46.8%) | (in months) Expressed in Median (IQR) Overall: 4.0 (3.0, 58.5) Smokers: 6.0 (3.0, 62.0) Non-smokers: 4.0 (3.0, 57.25) | Primary. Questionnaire and HbA1c test performed. Patient information including disease-related, clinical and DEC utilization variables were extracted from the centers’ medical records | tertiary | Health Grouping/Profiling | 1. Smoking status | Judgemental Splitting by patients' sociodemographic and/or economic attributes | SPSS 12 | 2 | 1. Smoker 2. Non-smoker |
| 52 | Gunathilake W et al (2010) | Cardiovascular and metabolic risk profiles in young and old patients with type 2 diabetes | Europe | England, Wales | Cross-sectional study | 49919 | Adult T2DM patients aged above 18 years old without previous CVD events and not on lipid or blood pressure lowering therapy | Expressed as Mean 1. <40 years old: 33.8 years old 2. >40 years old: 66.9 years old | Not specified | Not specified | Secondary data Administrative healthcare records | Primary | Assess differential risk of diabetic related complications across groups | 1. Patients' age | Judgemental Splitting by patients' sociodemographic and/or economic attributes | SPSS for Windows | 2 | 1. <40 years old 2. >40 years old |
| **S/No** | **Authors (Year)** | **Article Name** | **Continent** | **Country** | **Study design** | **Sample size** | **T2DM Patient population** | **Mean / Median Age of patients** | **Gender [Male (%)]** | **Duration of diabetes** | **Data source** | **Study setting** | **Objective of segmentation** | **Segmentation variables** | **Statistical methods used** | **Software** | **Number of segments** | **Categories of segments** |
| 53 | Gunzler D et al (2017) | Psychosocial Features of Clinically Relevant Patient Subgroups With Serious Mental Illness and Comorbid Diabetes. | North America | United States of America | Cross-sectional study | 200 | Adult T2DM patients aged ≥ 18 years of age, having schizophrenia, schizoaffective disorder, bipolar disorder or major depression confirmed with the Mini-International Neuropsychiatric Interview (MINI)7 | Expressed as Mean(SD) 52.7 (9.5) years | 72 (36%) | not specified | Secondary. Baseline data from 200 participants enrolled in a randomized controlled  trial testing a novel self-management intervention vs. treatment as usual among seriously mentally ill patients comorbid for diabetes (1R01MH085665) | not specified | Health Grouping/Profiling | 1. Montgomery Asberg Depression Rating Scale score 2. Brief Psychiatric Rating Scale score 3. Multidimensional Scale of Perceived Social Support score 4. Perceived Therapeutic Efficacy Scale score | Latent class analysis | not specified | 2 | 1. Group A “Highly symptomatic, low resources” 2. Group B “Less symptomatic, more resources” |
| 54 | Hanai K et al (2012) | Gender differences in the association between HDL cholesterol and the progression of diabetic kidney disease in type 2 diabetic patients. | Asia | Japan | Prospective cohort study | 723 | Adult T2DM patients from ambulatory cae and hospitalized patients presenting at the Diabetes Centre, Tokyo Women’s Medical University Hospital | Expressed as Mean(SD) 1. Men: 61 (11) 2. Women: 65 (11) | 443 (61.3%) | Expressed as Mean(SD) 1. Men: 13 (8) 2. Women: 13 (8) | Primary. baseline anthropometric and physical examinations. Laboratory examinations | tertiary | Assess differential risk of diabetic related complications across groups | 1. Patient's gender | Judgemental Splitting by patients' sociodemographic and/or economic attributes | SAS version 9.2 | 2 | 1. Men 2. Women |
| 55 | Handisurya A et al (2011) | Clinical characteristics, modalities and complications of diabetic patients with migration background at a Central European University Clinic. | Europe | Austria | Cross-sectional study | 200 | 1. Singleton pregnancies in women with type 2 diabetes 2. Non-pregnant type 2 diabetes subjects age <= 75 and a documented history of presence or absence of coronary heart disease | 1. Expressed as Mean (SD) 2. Non-immigrants: 36.14 (5.22) 3. Immigrants: 35.14 (5.24) | 0% | Not specified | Secondary  Patient register | Tertiary | - Health Grouping/Profiling - Assess differential risk of diabetic related complications across groups - Address pregnancy related outcomes | 1. Citizenship (Immigrant or non-immigrant) | Judgemental Splitting by patients' sociodemographic and/or economic attributes | SAS Enterprise Guide 4.1 | 2 | 1. Immigrants 2. Non-immigrants |
| **S/No** | **Authors (Year)** | **Article Name** | **Continent** | **Country** | **Study design** | **Sample size** | **T2DM Patient population** | **Mean / Median Age of patients** | **Gender [Male (%)]** | **Duration of diabetes** | **Data source** | **Study setting** | **Objective of segmentation** | **Segmentation variables** | **Statistical methods used** | **Software** | **Number of segments** | **Categories of segments** |
| 56 | Hari KKV et al (2014) | Clinical profile of patients using normal, high and very high insulin doses in type 2 diabetes. | Asia | India | Cross-sectional study | 60 | T2DM patients aged 30–75 years, using stable insulin dose for last 6 months, HbA1c between 6–7.5% with negative screening tests for Acromegaly and Cushing’s disease | Expressed as Mean(SD) 1. Group 1: 51.7 (13.3) 2. Group 2: 57.6 (11.5) 3. Group 3: 55.3 (9.7) | 1. Group 1: 13(43.3%) 2. Group 2: 11(55%) 3. Group 3: 3(30%) | Expressed as Mean(SD) 1. Group 1: 9.9 (4.8) 2. Group 2: 10.1 (5) 3. Group 3: 10 (4.1) | Primary. clinical examination | Tertiary | Health Grouping/Profiling | 1. Total daily insulin requirement (TDIR) | Judgemental splitting by patients' clinical characteristics | Graph Pad Prism Software, Version 5 | 3 | 1. Group 1 (TDIR <1 U/kg) 2. Group 2 (TDIR 1–2 U/kg)  3. Group 3 (TDIR > 2 U/kg). |
| 57 | Harris EL et al (1999) | Black-white differences in risk of developing retinopathy among individuals with type 2 diabetes. | North America | United States of America | Prospective cohort study | 105 | Adult T2DM patients aged 40–69 years | Expressed as Median (IQR) Overall: 58 (50-63) 1. White women: 55 (51-60) 2. Black women: 55 (48-62) 3. White men: 60 (54-63) 4. Black men: 60.5 (48-65) | 50 (45.5%) | Expressed as n(%) <5 years: 65 (59.1%) 5-9 years: 22 (20.0%) =>10 years: 23 (20.9%) | Primary  Questionnaires and blood tests | Tertiary | Assess differential risk of diabetic related complications across groups | 1. Patients' gender 2. Patients' ethnicity | Judgemental Splitting by patients' sociodemographic and/or economic attributes | Not specified | 4 | 1. White women 2. Black women 3. White men 4. Black men |
| 58 | Harris MI et al (1999) | Racial and Ethnic Differences in Glycemic Control of Adults With Type 2 Diabetes | North America | United States of America | Prospective cohort study | 1480 | T2DM patients aged 20 years old and above | Mean age at diagnosis 52.0 years | Total: 645 (43.6%) Non-Hispanic White: 278 (47.1%) Non-hispanic Black: 166 (41.0%) Mexican American: 189 (42%) | Expressed as Mean 9.5 years | Primary  National Health and Nutrition Examination Survey | Primary | Assess diabetic control across groups | 1. Patients' ethnicity | Judgemental Splitting by patients' sociodemographic and/or economic attributes | SAS | 3 | 1. Non-Hispanic Caucasian 2. Non-Hispanic African-American 3. Mexican-American |
| 59 | Harris MI et al (2001) | Racial and Ethnic Differences in Health Care Access and Health Outcomes for Adults With Type 2 Diabetes | North America | United States of America | Prospective cohort study | 1480 | T2DM patients aged 25 years old and above | Median Age 1. Non-Hispanic Caucasian: 63.4 2. Non-Hispanic African American: 59.5 3. Mexican-African: 56.1 | 1. Non-Hispanic Caucasian: 46% 2. Non-Hispanic African American: 37.3% 3. Mexican-African: 38.7% | Median duration since diabetes diagnosis 1. Non-Hispanic Caucasian: 6.4 2. Non-Hispanic African American: 7.6 3. Mexican-African: 5.4 | Primary  National Health and Nutrition Examination Survey | Primary | Assess non-diabetes metabolic derangements (e.g. lipid, blood pressure) across groups Assess diabetic control across groups Assess adherence to medical follow-up Assess diabetic control across groups | 1. Patients' ethnicity | Judgemental Splitting by patients' sociodemographic and/or economic attributes | SAS | 3 | 1. Non-Hispanic Caucasian 2. Non-Hispanic African-American 3. Mexican-American |
| 60 | Herman WH et al (2009) | Racial and ethnic differences in mean plasma glucose, hemoglobin A1c, and 1,5-anhydroglucitol in over 2000 patients with type 2 diabetes. | Australia, Asia, Europe and North America | Argentina, Australia, Brazil, Canada, Greece, Hungary, India, Romania, Spain, The Netherlands and the United States | Cross-sectional study | 2094 | Adult T2DM patients aged 30–80 years old with HbA1c more than 7.0% on at least two oral antihyperglycemic agents (OHAs) | Expressed as Mean(SD) All: 57 (10)  1. Caucasian: 59 (10)  2. African: 55 (11) 3. Hispanic: 54 (10) 4. Asian: 53 (9) 5. Others: 54 (9) | All: 1110 (53%) 1. Caucasian: 752 (57%) 2. African: 63 (47%) 3. Hispanic: 108 (43%) 4. Asian: 151 (47%) 5. Others: 33 (49%) | Expressed as Mean(SD) All: 9.5 (6.1) 1. Caucasian: 9.8 (6.1)  2. African: 8.8 (7.4)  3. Hispanic: 9.6 (6.0) 4. Asian: 8.3 (5.5)  5. Others: 10.9 (6.8) | Secondary data from the DURABLE trial where A1c, 1,5-AG, and mean plasma glucose (MPG) were done routinely. | not specified | Assess diabetic control across groups | 1. Patients' race and ethnicity | Judgemental Splitting by patients' sociodemographic and/or economic attributes | SAS version 8.02 | 5 | 1. Caucasian 2. African 3. Hispanic 4. Asian 5. Others |
| **S/No** | **Authors (Year)** | **Article Name** | **Continent** | **Country** | **Study design** | **Sample size** | **T2DM Patient population** | **Mean / Median Age of patients** | **Gender [Male (%)]** | **Duration of diabetes** | **Data source** | **Study setting** | **Objective of segmentation** | **Segmentation variables** | **Statistical methods used** | **Software** | **Number of segments** | **Categories of segments** |
| 61 | Hermans MP et al (2002) | Clinical, biophysical and biochemical variables from African-heritage subjects with type 2 diabetes. | Europe | Belgium | Cross-sectional study | 337 | T2DM patients with Belgian or Bantu ethnicity who followed up in diabetes clinic | Expressed as Mean(SD) 1. Belgian: 66 (11) 2. Bantu: 52 (10) | 1. Belgian: 185 (59%) 2. Bantu: 14 (61%) | Expressed as Mean (SD) 1. Belgian: 14 (9) 2. Bantu: 9 (9) | Primary  Clinical assessment, questionnaires and blood tests | Tertiary | Health Grouping/Profiling Assess differential risk of diabetic related complications across groups Assess non-diabetes metabolic derangements (e.g. lipid, blood pressure) across groups Assess diabetic control across groups Assess treatment outcomes | 1. Patients' ethnicity | Judgemental Splitting by patients' sociodemographic and/or economic attributes | Unspecified | 2 | 1. Belgian 2. Bantu |
| 62 | Hong CY et al (2004) | Ethnic differences among Chinese, Malay and Indian patients with type 2 diabetes mellitus in Singapore | Asia | Singapore | Cross-sectional study | 967 | Adult T2DM patients on follow-up in polyclinic | Expressed in Mean(SD) 1. Chinese: 61.6 (10.6) 2. Malay: 56.0 (11.0) 3. Indian: 59.9 (10.6) | 1. Chinese: 397 (50.1%) 2. Malay: 31 (44.9%) 3. Indian: 52 (49.1%) | Median (IQR) 1. Chinese: 7.0 (9.0) 2. Malay: 4.0 (8.0) 3. Indian: 6.5 (11.0) | Primary  Interview, clinical records, blood tests | Tertiary | Health Grouping/Profiling Assess diabetic control across groups  Assess differential risk of diabetic related complications across groups | 1. Patients' ethnicity | Judgemental Splitting by patients' sociodemographic and/or economic attributes | SPSS for Windows | 3 | 1. Chinese 2. Malay 3. Indian |
| 63 | Hsu CC et al (2014) | Associations between dietary patterns and kidney function indicators in type 2 diabetes | Asia | Taiwan | Cross-sectional study | 635 | Adult T2DM patients aged 30 to 70 years old | Expressed as Mean(SD) High fat dietary pattern scores Tertile 1: 63.83 (7.78) Tertile 2: 62.12 (7.94) Tertile 3: 57.75 (8.54)  Fish and vegetable dietary pattern scores Tertile 1: 60.23 (8.55) Tertile 2: 60.75 (8.27) Tertile 3: 62.70 (8.45)  Traditional Chinese snack dietary pattern score Tertile 1: 62.89 (7.96) Tertile 2: 60.95 (8.41) Tertile 3: 59.86 (8.80) | High fat dietary pattern scores Tertile 1: 61 (28.9%) Tertile 2: 104 (49.1%) Tertile 3: 128 (60.4%)  Fish and vegetable dietary pattern scores Tertile 1: 93 (44.1%) Tertile 2: 95 (44.8%) Tertile 3: 105 (49.5%)  Traditional Chinese snack dietary pattern score Tertile 1: 100 (47.4%) Tertile 2: 106 (50.0%) Tertile 3: 87 (41.0%) | Expressed as Mean(SD)  High fat dietary pattern scores  Tertile 1: 11.28 (7.05)  Tertile 2: 10.72 (5.17) Tertile 3: 9.70 (4.34)   Fish and vegetable dietary pattern scores  Tertile 1: 10.47 (6.24) Tertile 2: 10.22 (5.20)  Tertile 3: 11.02 (5.5)   Traditional Chinese snack dietary pattern score  Tertile 1: 11.62 (7.00)  Tertile 2: 10.61 (5.11)  Tertile 3: 9.48 (4.40) | Primary  Interview, clinical records, blood test | Primary | Assess differential risk of diabetic related complications across groups | 1. High fat dietary pattern scores 2. Fish and vegetable dietary pattern scores 3. Traditional Chinese-snack dietary pattern scores | Judgemental Splitting by patients' lifestyle habits | SPSS for Windows 17 | 1) High fat dietary pattern scores: 3 2) Fish and vegetable dietary pattern scores: 3  3) Traditional Chinese-snack dietary pattern score: 3 | High fat dietary pattern scores 1. Tertile 1 2. Tertile 2 3. Tertile 3  Fish and vegetable dietary pattern scores 1. Tertile 1 2. Tertile 2 3. Tertile 3  Traditional Chinese-snack dietary pattern scores 1. Tertile 1 2. Tertile 2 3. Tertile 3 |
| **S/No** | **Authors (Year)** | **Article Name** | **Continent** | **Country** | **Study design** | **Sample size** | **T2DM Patient population** | **Mean / Median Age of patients** | **Gender [Male (%)]** | **Duration of diabetes** | **Data source** | **Study setting** | **Objective of segmentation** | **Segmentation variables** | **Statistical methods used** | **Software** | **Number of segments** | **Categories of segments** |
| 64 | Jeong JH et al (2016) | Depression and mortality in people with type 2 diabetes mellitus, 2003 to 2013: A nationwide population-based cohort study | Asia | South Korea | Retrospective Cohort Study | 1,043,089‬ | T2DM patients age >30 who accepted at least one prescription of antidiabetic medication. Prevalence of depression included having at least one prescription for antidepressants | Expressed as Mean(SD) 1. With depression: 61.51 (10.81) 2. Without depression: 59.08 (11.48) | 1. With depression: 15,403 (39.2%) 2. Without depression: 526,723 (52.5%) | not specified | -Secondary -National Health Information database | not specified | - Assess mortality | 1. Presence of depression | Judgemental splitting by patients' clinical characteristics | not specified | 2 | 1. With depression 2. Without depression |
| 65 | Ji L et al (2014) | Hyperglycemia and duration of diabetes as risk factors for abnormal lipids: a cross sectional survey of 19,757 patients with type 2 diabetes in China. | Asia | China | Cross-sectional study | 19757 | Adult T2DM patients aged 18 years of age or more who were treated with oral antidiabetes drugs (OADs) alone or OADs combined with other drugs | Expressed in Mean(SD) 1. LDL-C<2.6 mmol/L: 59.0 (11.7)  2. LDL-C >=2.6 mmol/L: 59.4 (10.9) | 1. LDL-C<2.6 mmol/L: 3908 (51.5%) 2. LDL-C >=2.6 mmol/L: 6612 (54.3%) | Expressed as Median (IQR) 1. LDL-C<2.6 mmol/L: 3.93 (1.50–8.28) 2. LDL-C >=2.6 mmol/L: 4.11 (1.89–9.11) | Primary questionnaire to collect demographic information and to record clinical profile, results of laboratory essays and medical records | tertiary | Assess differential risk of metabolic related complications across groups | 1. LDL-C level | Judgemental splitting by patients' clinical characteristics | SAS version 9.3 | 2 | 1. LDL-C<2.6 mmol/L 2. LDL-C >=2.6 mmol/L |
| 66 | Jiang R et al (2018) | Clinical Trajectories, Healthcare Resource Use, and Costs of Diabetic Nephropathy Among Patients with Type 2 Diabetes: A Latent Class Analysis | North America | United States of America | Cross-sectional study | 23,235 | T2DM adult patients (aged > 18 years) with 2 distinct medical claims and with > 2 urine albumin test results after the ﬁrst observed T2DM diagnosis. | Expressed as Mean(SD) 1. Latent Class I: 54.5 (9.2) 2. Latent Class II: 54.4 (8.6)  3. Latent Class III: 49.1 (13.3) 4. Latent Class IV: 58.2 (8.7) | 1. Latent Class I: 5795 (54%) 2. Latent Class II: 3789 (56%)  3. Latent Class III: 1199 (53%) 4. Latent Class IV: 1965 (57%) | Expressed as Mean(SD) 1. Latent Class I: 32.9 (25.1) 2. Latent Class II: 42.0 (26.8) 3. Latent Class III: 42.6 (26.9) 4. Latent Class IV: 46.6 (29.6) | Secondary. Truven Health Analytics MarketScan Commercial and Medicare Supplemental and Lab databases | Not specified | Health grouping / profiling Assess differential healthcare utilization | 1. Microvascular disease (retinopathy disease, neuropathy disease, cardiovascular disease, ischemic heart disease, cerebrovascular disease, chronic heart failure, hypertension) 2. CKD-related disease 3. Metabolic disorder 4. Use of diabetic treatment (Metformin, sulphonylureas, insulin, DPP4 inhibitors, GLP1-based therapy and other antidiabetic agents) | Latent class analysis | SAS version 9.4 | 4 | 1. Low comorbidity/low treatment 2. Low comorbidity/high treatment 3. Moderate comorbidity/high insulin use 4. High comorbidity/moderate treatment |
| **S/No** | **Authors (Year)** | **Article Name** | **Continent** | **Country** | **Study design** | **Sample size** | **T2DM Patient population** | **Mean / Median Age of patients** | **Gender [Male (%)]** | **Duration of diabetes** | **Data source** | **Study setting** | **Objective of segmentation** | **Segmentation variables** | **Statistical methods used** | **Software** | **Number of segments** | **Categories of segments** |
| 67 | Johnson JF et al (2017) | Real-world Clinical Outcomes Among Patients With Type 2 Diabetes Receiving Canagliflozin at a Specialty Diabetes Clinic: Subgroup Analysis by Baseline HbA(1c) and Age. | North America | United States of America | Cross-sectional study | 462 | Adult T2DM patients were aged >= 18 years, of active status in the clinic, had received initial and regular follow-up care in the clinic, had received the initial prescription for canagliflozin from the clinic, and had returned to the clinic for >= 1 follow-up after canagliflozin was prescribed. | Expressed as Mean(SD) 1. Age <65 y: 52.9(8.1) 2. Age >=65 y: 69.8(4.0) | 1. Age <65 y: 234 (59.1%) 2. Age >=65 y: 43 (65.2%) | Expressed as Mean(SD) 1. Age <65 y: 12.0(6.1) 2. Age >=65 y: 16.6(8.3) | secondary. review of data from the electronic health records of adults with T2DM | tertiary | Assess treatment outcomes | 1. Patients' age | Judgemental Splitting by patients' sociodemographic and/or economic attributes | not specified | 2 | 1. Age <65 years old 2. Age >= 65 years old |
| 68 | Kalsekar ID et al (2006) | Impact of depression on utilization patterns of oral hypoglycemic agents in patients newly diagnosed with type 2 diabetes mellitus: a retrospective cohort analysis. | North America | United States of America | Retrospective cohort study | 1237 | Newly diagnosed adult T2DM patients aged <65 years during a 3-year period (1998-2000) | Expressed as Mean(SD) 1. Depressed: 46.79 (9.32) 2. Non-depressed: 49.03 (10.74) | 1. Depressed: 81 (18.4%) 2. Non-depressed: 371 (47.3%) | NA. Newly diagnosed | secondary; West Virginia Medicaid medical and pharmacy claims data for the period January 1, 1997, through December 31, 2002 | tertiary | Assess treatment outcomes | 1. Presence of preexisting depression | Judgemental splitting by patients' clinical characteristics | SPSS version 13.0 | 2 | 1. Depressed 2. Non-depressed |
| 69 | Kaplan SH et al (2013) | Reducing racial/ethnic disparities in diabetes: the Coached Care (R2D2C2) project. | North America | United States of America | Cross-sectional study | 1484 | Adult T2DM patients aged 18 years of age or older who were Mexican American, Vietnamese American or non- Hispanic white | Expressed as Mean(SD) 1. Non-hispanic White: 60.6 (10.7)  2. Mexican American: 55.5 (10.8) 3. Vietnamese American: 67.3 (10.0) | 1. Non-hispanic White: 216 (55.5%) 2. Mexican American: 258 (33.0%) 3. Vietnamese American: 125 (39.9%) | Expressed as Mean(SD) 1. Non-hispanic White: 8.8 (7.3) 2. Mexican American:9.7 (7.4) 3. Vietnamese American:9.3 (7.5) | Primary. Baseline survey. Laboratory, administrative and medical records data | primary and tertiary | Health Grouping/Profiling - Assess diabetic control across groups - Assess differential healthcare utilization - Assess treatment outcomes | 1. Patients' race and ethnicity | Judgemental Splitting by patients' sociodemographic and/or economic attributes | SPSS v. 20.0, Feldt’s W statistic | 3 | 1. Non-Hispanic White 2. Mexican American 3. Vietnamese American |
| 70 | Karpati T et al (2018) | Patient clusters based on HbA1c trajectories: A step toward individualized medicine in type 2 diabetes. | Asia | Israel | Retrospective cohort study | 60,423 | Adult T2DM patients with at least three years of continuous membership at Clalit prior to the included in the Clalit diabetes registry and short to medium duration type 2 diabetes | Expressed as Mean(SD) Overall: 63.6 (13.4)  1. Stable: 66.0 (12.0)  2. Descending: 62.0 (12.2)  3. Ascending: 59.9 (12.0)  4. Undefined: 61.0 (15.4) | Overall: 40654 (47.4%) 1. Stable: 20366 (44.6%) 2. Descending: 3023 (49.7%)  3. Ascending: 4298 (49.6%)  4. Undefined: 12967 (51.1%) | Expressed in months as Mean (SD) Overall: 62.4 (14.1)  1. Stable: 60.8 (14.1)  2. Descending: 65.6 (13.8)  3. Ascending: 64.0 (14.0)  4. Undefined: 62.6 (14.1) | Secondary. All-cause mortality, demographic, clinical, and laboratory data were obtained from the Clalit Health Services (Clalit) healthcare data warehouse | not specified | Health Grouping/Profiling - Assess differential risk of diabetic related complications across groups | 1. HbA1c trajectories | Cluster analysis | R statistical software version 3.2.2, R package (version 1.2) | 4 | 1. Stable cluster: Patients with a stable HbA1c trend over time 2. Decreasing cluster: patients with a descending trend over time  3. Ascending cluster: patients with an ascending trend over time; undefined cluster |
| **S/No** | **Authors (Year)** | **Article Name** | **Continent** | **Country** | **Study design** | **Sample size** | **T2DM Patient population** | **Mean / Median Age of patients** | **Gender [Male (%)]** | **Duration of diabetes** | **Data source** | **Study setting** | **Objective of segmentation** | **Segmentation variables** | **Statistical methods used** | **Software** | **Number of segments** | **Categories of segments** |
| 71 | Kaukua J et al (2001) | Clustering of cardiovascular risk factors in type 2 diabetes mellitus: prognostic significance and tracking. | Europe | Finland | Prospective cohort study | 133 | Patients aged 45-64 years with newly diagnosed T2DM from a defined area in Kuopio | Expressed as Mean (SD) 55.7 (0.8) | 70 (53%) | not specified | -primary -Baseline examination (medical history, questionnaires, patient clinical data and patient attributes | -primary | 1) Health Grouping/Profiling 2) Assess differential risk of diabetic related complications across groups 3) Assess non-diabetes metabolic derangements (e.g. lipid, blood pressure) across groups | Number of risk factors 1. Smoking 2. High body mass index 3. Elevated systolic blood pressure 4. High serum low density lipoprotein (LDL) cholesterol 5. High serum triglycerides 6. Low serum high density lipoprotein (HDL) cholesterol 7. High fasting blood glucose 8. High plasma insulin concentration) | Cluster analysis | SPSS for windows | 4 | 1. 0 risk factors 2. 1 or 2 risk factors 3. 3 or 4 risk factors 4. 5 or more risk factors |
| 72 | Kautzky-Willer A et al (2010) | Sex-specific differences in metabolic control, cardiovascular risk, and interventions in patients with type 2 diabetes mellitus. | Europe | Austria | Cross-sectional study | 201 | Adult T2DM patients age ≤75 years with a documented history of presence or absence of coronary heart disease (CHD) | Expressed in Mean(SE) 1. Women: 60.5 (8.6)  2. Men: 58.2 (9.6) | 108 (53.7%) | Expressed in Mean(SE) 1. Women: 10.1 (7.7) 2. Men: 8.4 (7.0) | primary. Questionnaire and clinical records | tertiary | - Assess differential risk of diabetic related complications across groups  - Assess non-diabetes metabolic derangements (e.g. lipid, blood pressure) across groups  - Assess diabetic control across groups  - Assess treatment outcomes | 1. Patient's gender | Judgemental Splitting by patients' sociodemographic and/or economic attributes | SPSS 16.0 | 2 | 1. Women  2. Men |
| 73 | Ki M et al (2014) | Age-related differences in diabetes care outcomes in Korea: a retrospective cohort study. | Asia | Korea | Retrospective cohort study | 4471 | Age 40-79 with T2DM diagnosed at 2001 or newly diagnosed between 2002 and 2006 and 4 or more outpatient visits for diabetes between 2007 and 2008 | Expressed as N(%) 1. Age =<59: 2291 (51.2%) 2. Age >=60: 2180 (48.8%) | 1. Age =<59: 1515 (66%) 2. Age >=60: 1176 (54%) | Expressed as N(%) 1. Age =<59 3-5 years: 861 (38%) 6-8 years: 608 (27%) >=9 years: 822 (36%)  2. Age >=60 3-5 years: 607 (28%) 6-8 years: 529 (24%) >=9 years: 1044 (48%) | - Secondary data - Korean National Health Insurance claims data | Not specified | 1) Health Grouping/Profiling 2) Assess differential healthcare utilization  3) Assess diabetic control across groups | 1. Patients' age | Judgemental Splitting by patients' sociodemographic and/or economic attributes | SAS 9.1 for Windows | 2 | 1. Age =<59 years old (middle-aged) 2. Age >=60 years old (Older) |
| 74 | Klisic A et al (2019) | Association between unfavorable lipid profile and glycemic control in patients with type 2 diabetes mellitus | Europe | Montanegro | Cross-sectional study | 275 | Sedentary patients with DM2 without acuite inflammatory disease, with no history or presence of malignancy | Expressed as mean(interquartile range) 1. First HbA1C Tertile (<6.26%): 61.00 (52.25 - 66.00) 2. Second HbA1C Tertile (6.27-7.19%): 61.00 (54.25 - 65.00) 3. Third HbA1C Tertile (>=7.20%): 62.00 (55.75 - 68.00) | 1. First HbA1C Tertile (<6.26%): 45 (49.5%) 2. Second HbA1C Tertile (6.27-7.19%): 43 (45.3%) 3. Third HbA1C Tertile (>=7.20%): 46 (52.3%) | Expressed as mean(interquartile range) 1. First HbA1C Tertile (<6.26%): 3.00 (2.00 - 6.75) 2. Second HbA1C Tertile (6.27-7.19%): 3.00 (1.00 - 5.00) 3. Third HbA1C Tertile (>=7.20%): 4.00 (1.75 - 9.25) | - Primary - Self-administered questionnaire | Primary | 1) Assess non-diabetes metabolic derangements (e.g. lipid, blood pressure) across groups | 1. Patients' HbA1c level | Judgemental splitting by patients' clinical characteristics | PASW Statistic Version 18 | 3 | 1. First HbA1C Tertile (<6.26%) 2. Second HbA1C Tertile (6.27-7.19%) 3. Third HbA1C Tertile (>=7.20%) |
| **S/No** | **Authors (Year)** | **Article Name** | **Continent** | **Country** | **Study design** | **Sample size** | **T2DM Patient population** | **Mean / Median Age of patients** | **Gender [Male (%)]** | **Duration of diabetes** | **Data source** | **Study setting** | **Objective of segmentation** | **Segmentation variables** | **Statistical methods used** | **Software** | **Number of segments** | **Categories of segments** |
| 75 | Kuznetsov VA et al (2010) | Clinical manifestations and risk factors of coronary artery disease in patients with diabetes mellitus in western Siberia. | Asia | Russia | Cross-sectional study | 382 | T2DM patients with angiographic CAD (>50% stenosis) from admission records at the Tyumen Cardiology Centre, Russia | Expressed as Mean (SD) 1. Northern Patients: 53.4 (6.7) 2. Southern patients: 56.9 (6.9) | 1. Northern Patients: 197 (81.1%) 2. Southern patients: 103 | Expressed as Mean (SD) 1. Northern Patients: 4.7 (5.4) 2. Southern patients: 6.1 (6.98) | -Secondary -Admission records at the Tyumen Cardiology Centre, Russia | tertiary | - Assess differential risk of diabetic related complications across groups - Assess non-diabetes metabolic derangements (e.g. lipid, blood pressure) across groups | 1. Living at northern vs southern latitudes | Judgemental Splitting by patients' sociodemographic and/or economic attributes | not specified | 2 | 1. Northern Latitude  2. Southern Latitude |
| 76 | Lee CL et al (2018) | Trajectories of fasting plasma glucose variability and mortality in type 2 diabetes. | Asia | Taiwan | Retrospective cohort study | 3569 | Adult T2DM outpatients aged > 18 years | Expressed in Mean(SD) 1. FPG variability trajectory groups Low: 68.1 (13.6) Increasing: 71.2 (13.9) Fluctuating: 71.8 (3.1) Decreasing 70.4 (14.8)  High: 70.0 (18.5)  2. Mean FPG trajectory groups Well controlled: 69.6 (13.6) Stable: 68.7 (13.2) Worsening : 65.8 (14.9) Improving: 67.0 (14.3) Poor: 62.2 (15.7) | 1. FPG variability trajectory groups Low: 1662 (54.9%) Increasing: 64 (58.2%) Fluctuating: 140 (56.5%) Decreasing 60 (50.4%  High: 32 (47.8%)  2. Mean FPG trajectory groups Well controlled: 857 (58.1%) Stable: 774 (53.2%) Worsening : 162 (52.4%) Improving: 121 (50.6%) Poor: 44 (47.3%) | Not specified | primary. FPG was measured every 3 months for 2 years, participants’ demographic data,  laboratory tests and medications were taken from hospital records. | tertiary | - Assess mortality - Assess diabetic control across groups | 1. Fasting plasma glucose variability  2. Mean fasting blood glucose | Group-based trajectory modelling analysis | SAS software  (version 9.4). | Fasting plasma glucose variability: 5 Mean fasting blood glucose: 5 | Fasting plasma glucose variability  1. Low 2. Increasing 3. Fluctuating 4. Decreasing 5. High  Mean fasting blood glucose 1. Well controlled 2. Stable control 3. Worsening control  4. Improving control  5. Poor control |
| **S/No** | **Authors (Year)** | **Article Name** | **Continent** | **Country** | **Study design** | **Sample size** | **T2DM Patient population** | **Mean / Median Age of patients** | **Gender [Male (%)]** | **Duration of diabetes** | **Data source** | **Study setting** | **Objective of segmentation** | **Segmentation variables** | **Statistical methods used** | **Software** | **Number of segments** | **Categories of segments** |
| 77 | Lee PG et al (2018) | Patterns of physical activity in sedentary older individuals with type 2 diabetes | North America | United States of America | Cross-sectional study | 115 | Adult T2DM patients aged ≥60 years with the ability to walk across a small room without an assistive device | Expressed as Mean(SD) Total 70.6 (7.1) 1. Class 1: 70.5 (7.8)  2. Class 2: 70.1 (5.8) 3. Class 3: 71.6 (6.7) | Total: 49 (40%) 1. Class 1: 18 (26.5%)  2. Class 2: 11 (52.4%) 3. Class 3: 13 (72.2%) | not specified | Secondary, Community Healthy Activities Model Program for Seniors (CHAMPS) survey | tertiary | Health Grouping/Profiling | 1. Patterns of physical activity | Latent class analysis | Latent Gold 4.0 and SAS 9.3 software programs. | 3 | 1. Latent class 1: Primarily reported domestic-focused activities (60.9%)  2. Latent class 2: High probability of reporting domestic-focused activities, in addition to leisure-time physical activities and structured exercise activities, specifically heavy gardening, walking to do errands, walking leisurely, and stretching 3. Latent class 3: High probability of reporting domestic-focused activities, in addition to leisure-time physical activities and structured exercise activities, specifically heavy housework, walking leisurely, stretching, and strength training. |
| **S/No** | **Authors (Year)** | **Article Name** | **Continent** | **Country** | **Study design** | **Sample size** | **T2DM Patient population** | **Mean / Median Age of patients** | **Gender [Male (%)]** | **Duration of diabetes** | **Data source** | **Study setting** | **Objective of segmentation** | **Segmentation variables** | **Statistical methods used** | **Software** | **Number of segments** | **Categories of segments** |
| 78 | Li C et al (2007) | Clustering of multiple healthy lifestyle habits and health-related quality of life among U.S. adults with diabetes. | North America | United States of America | Cross-sectional study | 16428 | Adult T2DM (Age >=18 years old) men and nonpregnant women | Expressed as Mean (SE) 58.9 (0.11) | Total: 8674 (52.8%)  Single Healthy Lifestyle Habit (HLH)  1. Not smoking (NSMK): 78.4% 2. Adequste leisure time physical activity (LTPA):23.9% 3. Consuming >= 5 servings of fruit and veg daily (FVC5): 39.8%  Combined Healthy Lifestyle Habit (HLH)  1. 0 HLH: 11.0% 2. 1 HLH: 45.8% 3. 2 HLH: 33.5% 4. 3 HLH: 9.8% | Expressed as Mean (SE) 9.6 (0.08) | -secondary -Behavioural Risk Factor Surveillance SYstem (BRFSS) 2005 data conducted via a standardized telephone survey | -NA, telephone survey | - Health Grouping/Profiling - Assess differential QoL measures across groups | Healthy lifestyle habits 1. Smoking (number of cigarettes) 2. Physical activity level  3. Intake of fruits and vegetables daily  Combined number of healthy lifestyle habits | Judgemental splitting based on patients' lifestyle habits | SUDAAN software release 9.0 | Not smoking (NSMK): 2 Getting adequate leisure time physical activity (LTPA): 2  Consuming five or more servings of fruits and vegetables daily (FVC5): 2  Combined healthy lifestyle habits: 4 | Not smoking 1. Smokers 2. Non-smokers  Getting adequate leisure time physical activity (LTPA) 1. Getting adequate leisure time physical activity  2. Not getting adequate leisure time physical activity  Consuming five or more servings of fruits and vegetables daily (FVC5) 1. Consuming five or more servings of fruits and vegetables daily 2. Not consuming five or more servings of fruits and vegetables daily   Combined healthy lifestyle habits (HLH) 1. 0 HLHs  2. 1 HLHs 3. 2 HLHs 4. 3 HLHs |
| 79 | Li H et al (2019) | The Effect of Symptom Clusters on Quality of Life Among Patients With Type 2 Diabetes | North America | United States of America | Randomized controlled trial | 302 | Adult patients who 1. Were diagnosed with T2DM, hypertension, and hyperlipidemia 2. had taken at least 1 oral medication for each condition 3. had a 1-year or greater history of T2DM in the practice site and were able to be screened for adherence to each of the 3 target medications using the electronic medication monitors 4. were older than 40 years of age | Expressed as Mean(SD) [minimum0 maximum] 63.86 (10.11) [41-91] | 128 (42.4%) | not specified | secondary, data collected in a randomized controlled trial of adherence improving strategies among persons with T2DM, hypertension, and hypercholesterolemia (NIH, NIDDK RO1 DK59048). | primary | - Assess psychological symptoms across groups - Assess differential QoL measures across groups | 1. Severity of fatigue, depression, sleep disturbance, and anxiety at baseline | Hierarchical cluster analysis | SPSS, version 25.0. | 4 | 1. Subgroup 1 —severe symptoms of fatigue, impaired sleep quality, anxiety, and moderate depression. 2. Subgroup 2 —moderate symptoms of fatigue, impaired sleep quality, and mild symptoms of depression and anxiety. 3. Subgroup 3 —moderate symptoms of fatigue, impaired sleep quality, anxiety, and mild depression. 4. Subgroup 4 —low symptoms of fatigue, depression, anxiety, and mild impaired sleep quality. |
| **S/No** | **Authors (Year)** | **Article Name** | **Continent** | **Country** | **Study design** | **Sample size** | **T2DM Patient population** | **Mean / Median Age of patients** | **Gender [Male (%)]** | **Duration of diabetes** | **Data source** | **Study setting** | **Objective of segmentation** | **Segmentation variables** | **Statistical methods used** | **Software** | **Number of segments** | **Categories of segments** |
| 80 | Li L et al (2015) | Identification of type 2 diabetes subgroups through topological analysis of patient similarity. | North America | United States of America | Cross-sectional study | 11,210 | Adult T2DM patients participating in the Mount Sinai BioMe Biobank Program, an ongoing, EMR-linked bio- and data repository | 1. Mean or % subtype 1: 59.76 (0.45) 2. Mean or % subtype 2: 64.25 (0.50) 3. Mean or % subtype 3: 63.65 (0.3) | 4350 (39%) | not specified | primary. high-dimensional electronic medical records (EMRs) and genotype data | not applicable | Health Grouping/Profiling | Clinical variables 1. Full blood count 2. Age 3. Urine protein concentration 4. Blood biochemistries (serum creatinine, calcium, blood urea nitrogen, albumin) 5. BMI 6. Estimated glomerular filtration rate 7. INR, prothrombin time 8. Medication usage (Insulin, metformin, loop diuretics, DPP4 inhibitors, beta-blockers, ARB / ACE-inhibitors, vasodilators, nicotonic acid derivatives) | Cluster analysis | Ayasdi 3.0, SAS 9.3.2 (SAS Institute) and R 2.15.1 | 3 | 1. Subtype 1 (Youngest, with features classically associated with T2DM e.g. BMI, lowest complete blood count and better kidney function) 2. Subtype 2 (Lowest weight) 3. Subtype 3 (Highest systolic blood pressure, chloride levels, troponin I with higher ACE-inhibitors and statin usage) |
| 81 | Lim JH et al (2011) | Association between dietary patterns and blood lipid profiles in Korean adults with type 2 diabetes. | Asia | Korea | Retrospective cohort study | 680 | Age >30 who had completed a 24-hr recall in the dietary survey and undergone a health examination with anthropometric measurements | Expressed as Mean (SD) 1. Bread and Meat and Alcohol Quartile 1: 63.6 (10.4) Quartile 4: 56.5 (12.4)  2. Noodles & Seafood Quartile 1: 62.2 (11.3) Quartile 4: 57.7 (11.8)  3. Rice and vegetables Quartile 1: 60.6 (11.8) Quartile 4: 59.2 (12.3)  4. Korean Healthy Quartile 1: 61.3 (12.3) Quartile 4: 59.3 (11.8) | Expressed as Mean (SD) 1. Bread and Meat and Alcohol Quartile 1: 38.8% Quartile 4: 57.7%  2. Noodles & Seafood Quartile 1: 45.3% Quartile 4: 57.1%  3. Rice and vegetables Quartile 1: 42.4 Quartile 4: 61.2  4. Korean Healthy Quartile 1: 41.8 Quartile 4: 52.9 | Expressed as Mean (SD) 1. Bread and Meat and Alcohol Quartile 1: 9.6 (9.1) Quartile 4: 7.9 (7.7)  2. Noodles & Seafood Quartile 1: 7.8 (7.7) Quartile 4: 8.5 (8.3)  3. Rice and vegetables Quartile 1: 8.5 (8.5) Quartile 4: 8.6 (8.7)  4. Korean Healthy Quartile 1: 9.3 (9.7) Quartile 4: 8.6 (7.6) | - Secondary data - 4th Korean National Health and Nutrition Examination Survey (KNHANES) | Not specified | 1) Health Grouping/Profiling 2) Assess non-diabetes metabolic derangements (e.g. lipid, blood pressure) across groups | 1. Dietary Patterns | Latent class analysis | SAS 9.1 | 4 | 1. Bread & Meat & Alcohol 2. Noodles & Seafood 3. Rice & Vegetables 4. Korean Healthy |
| **S/No** | **Authors (Year)** | **Article Name** | **Continent** | **Country** | **Study design** | **Sample size** | **T2DM Patient population** | **Mean / Median Age of patients** | **Gender [Male (%)]** | **Duration of diabetes** | **Data source** | **Study setting** | **Objective of segmentation** | **Segmentation variables** | **Statistical methods used** | **Software** | **Number of segments** | **Categories of segments** |
| 82 | Lipscombe C et al (2015) | Exploring trajectories of diabetes distress in adults with type 2 diabetes; a latent class growth modeling approach. | North America | Canada | Prospective cohort study | 1135 | Adult T2DM patients aged 40-75 years old | Expressed as Mean(SD) 1. Trajectory 1: 60.96 (8.10) 2. Trajectory 2: 58.73 (7.79) 3. Trajectory 3: 58.00 (7.71) 4. Trajectory 4: 56.38 (8.67) 5. Trajectory 5: 55.93 (8.32) | Overall: 51.1% 1. Trajectory 1: 376 (54.18%) 2. Trajectory 2: 131 (49.62%) 3. Trajectory 3: 35 (43.75%) 4. Trajectory 4: 33 (48.53%) 5. Trajectory 5: 9 (31.03%) | Not specified | Primary  Clinical assessment and questionnaires | Primary | Assess differential risk of psychological outcomes across groups | 1. 17-item Diabetes Distress Scale scores | Latent class growth analysis | Not specified | 5 | 1. Trajectory 1: Persistently low diabetes distress 2. Trajectory 2: Decreasing moderate diabetes distress 3. Trajectory 3: Persistently severe diabetes distress 4. Trajectory 4: Persistently low but at-risk diabetes distress 5. Trajectory 5: Increasing moderate diabetes distress |
| 83 | Liu H et al (2017) | Body mass index and mortality in patients with type 2 diabetes mellitus: A prospective cohort study of 11,449 participants. | Asia | China | Prospective cohort study | 11,449 | Age >= 18 diagnosed with T2DM previously and had been taking glycemic control medications | Expressed as Mean(SD) All: 56.50 (10.65) 1. Underweight group <18.5 kg/m2: 59.80 (14.45) 2. Normal weight group 18.5–24 kg/m2: 57.70 (11.08) 3. Overweight group 24–28 kg/m2; 56.55 (10.22) 4. Obese group ≥28 kg/m2: 55.16 (10.71) | All: 9512 (83.1%) 1. Underweight group <18.5 kg/m2: 78 (84.8%) 2. Normal weight group 18.5–24 kg/m2: 2392 (82.7%) 3. Overweight group 24–28 kg/m2: 4575 (84.2%) 4. Obese group ≥28 kg/m2: 2467 (81.3%) | not specified | -Primary data -Medical examination and health information data collected in the Kailuan Study  - Mortality information from Social Security System of Kailuan | not specified | 1) Assess mortality | 1. Body mass index (BMI) | Judgemental splitting by patients' clinical characteristics | SPSS 13.0 | 4 | 1. Underweight group <18.5 kg/m2; 2. Normal weight group 18.5–24 kg/m2;  3. Overweight group 24–28 kg/m2; 4. Obese group ≥28 kg/m2. |
| 84 | Loh PT et al (2015) | Ethnic disparity in prevalence of diabetic kidney disease in an Asian primary healthcare cluster. | Asia | Singapore | Cross-sectional study | 57954 | Adult T2DM subjects age 21 and who had received care at NHG polyclinics between 2006 and 2009 | Expressed in Mean(SD) 1. Normoalbinuria 63.6 (10.7) 2. Microalbinuria 64.5 (11.6) 3. Macroalbinuria 67.3 (12.1)  4. renal impairment 74.2 (9.7)  5. Total 65.7 (11.5) | 1. Normoalbinuria 12398 (45.3%) 2. Microalbinuria 8485 (46.0%) 3. Macroalbinuria 823 (27.0%) 4. renal impairment 3478 (39.9%)  5. Total 25184 (43.7%) | Expressed in Mean(SD) 1. Normoalbinuria 8.1 (4.6) 2. Microalbinuria 8.2 (5.3)  3. Macroalbinuria 9.1 (6.3)  4. renal impairment 9.6 (6.5) 5. Total 8.4 (5.3) | -Secondary, Chronic Disease Management Registry (CDMR), | primary | - Health Grouping/Profiling  - Assess differential risk of diabetic related complications across groups | 1. Stages of diabetic kidney disease (DKD) | Judgemental splitting by patients' clinical characteristics | spss version 15.0 | 4 | 1. Normoalbuminuria 2. Microalbuminuria  3. Macroalbuminuria 4. Renal impairment |
| 85 | Lu B et al (2007) | High prevalence of chronic kidney disease in population-based patients diagnosed with type 2 diabetes in downtown Shanghai | Asia | China | Cross-sectional study | 1009 | Chinese T2DM patients aged over 30 in downtown Shanghai | **Expressed in Mean(SD) Men 1. CKD (-): 58.23 (11.52)  2. CKD (+): 69.76 (10.38)  Women 1. CKD (-): 60.88 (10.79) 2. CKD (+): 69.68 (10.05)** | 390 (38.7%) | **Expressed in Mean(SD) Men 1. CKD (-): 58.23 (11.52)  2. CKD (+): 69.76 (10.38)  Women 1. CKD (-): 60.88 (10.79) 2. CKD (+): 69.68 (10.05)** | Primary. Questionnaires, anthropometric measurements, laboratory assays and digital nonmydriatic fundus photography and image analysis | primary and tertiary | Assess differential risk of diabetic related complications across groups | 1. Presence of chronic kidney disease (CKD) 2. Patient gender | Judgemental splitting by patients' clinical characteristics, sociodemographic and/or economic attributes | SPSS for Windows version 11.0. | 4 | 1. Men with CKD (CKD+) 2. Men without CKD (CKD-) 3. Women with CKD (CKD+) 4. Men without CKD (CKD-) |
| **S/No** | **Authors (Year)** | **Article Name** | **Continent** | **Country** | **Study design** | **Sample size** | **T2DM Patient population** | **Mean / Median Age of patients** | **Gender [Male (%)]** | **Duration of diabetes** | **Data source** | **Study setting** | **Objective of segmentation** | **Segmentation variables** | **Statistical methods used** | **Software** | **Number of segments** | **Categories of segments** |
| 86 | Luo M et al (2017) | Longitudinal trends in HbA1c and associations with comorbidity and all-cause mortality in Asian patients with type 2 diabetes: A cohort study. | Asia | Singapore | Retrospective record linkage study | 6079 | Adult T2DM patients (21 years and above) from public sector primary care clinics and hospital outpatient clinics, who have at least one HbA1c measurement each year for three consecutive years before the time of recruitment | Age at baseline Expressed as Median(IQR) Total: 62 (55-70) 1. Group 1 (Low-stable): 64 (56–71) 2. Group 2 (Moderate-stable):60 (53–67) 3. Group 3 (Moderate-increase):55 (50–62) 4. Group 4 (High-decrease):57 (51–63) | Total: 2956 (48.6%) 1. Group 1 (Low-stable): 2124 (48.4%) 2. Group 2 (Moderate-stable): 656 (49.0%) 3. Group 3 (Moderate-increase): 86 (48.0%) 4. Group 4 (High-decrease): 90 (52.3%) | Expressed as Median (IQR) 1. Group 1 (Low-stable): 7.5 (4.6–13.2) 2. Group 2 (Moderate-stable): 11.9 (6.7–17.4) 3. Group 3 (Moderate-increase): 12.9 (8.0–17.8) 4. Group 4 (High-decrease):9.9 (4.2–16.1) | Primary. Data from Singapore Consortium of Cohort Studies Diabetes Cohort, including questionnaire, past medical records and prospective data linkage with the National Registry of Disease Office (NRDO) for recording of acute myocardial infarction (AMI), stroke, end stage renal failure (ESRD) and death events | primary and tertiary | Health Grouping/Profiling - Assess differential risk of diabetic related complications across groups - Assess non-diabetes metabolic derangements (e.g. lipid, blood pressure) across groups - Assess diabetic control across groups - Assess mortality | 1. HbA1c patterns | Latent class growth analysis | R version 3.3.2 | 4 | 1. Group 1 Low-stable 2. Group 2 Moderate-stable 3. Group 3 Moderate-increase 4. Group 4 High-decrease |
| 87 | Ma WY et al (2012) | Variability in hemoglobin A1c predicts all-cause mortality in patients with type 2 diabetes | Asia | Taiwan | Retrospective cohort study | 881 | T2DM patients who attended the Diabetes Shared Care Program between 2003 and 2009 at the Cardinal Tien Hospital | Expressed as Mean(SD) Hba1c variability (lower 50%): 60.0 (10.9) Hba1c variability (upper 50%): 59.3 (12.2) | 1. Hba1c variability (lower 50%): 219 (48%) 2. Hba1c variability (upper 50%): 203 (48%) | Expressed as Mean(SD) 1. Hba1c variability (lower 50%): 7.5 (5.6) 2. Hba1c variability (upper 50%): 9.3 (6.9) | Secondary  Electronic clinical records | Teritary | Assess mortality | 1. Hba1c variability | Judgemental splitting by patients' clinical characteristics | Stata/SE 11.0 | 2 | 1. Hba1c coefficient of variation lower 50% 2. Hba1c coefficient of variation upper 50% |
| 88 | Marinho FS et al (2015) | Profile of disabilities and their associated factors in patients with type 2 diabetes evaluated by the Canadian occupational performance measure: the Rio De Janeiro type 2 diabetes cohort study. | North America | Canada | Cross-sectional study | 475 | All adult T2DM aged up to 80 years old with either any microvascular or macrovascular complication, or with at least two other modifiable cardiovascular risk factors. | Expressed in Mean(SD) All: 59.1 (9.1)  1. Average performance >= 5 points: 58.8 (8.4)  2. Average performance <= 4 points: 59.4 (9.8) | All: 171 (36%) 1. Average performance >= 5 points: 92 (38.8%) 2. Average performance <= 4 points: 79 (33.5%) | Expressed in Median (IQR) All: 8 (3–15) 1. Average performance >= 5 points: 7 (3–15) 2. Average performance <= 4 points: 8 (3–15) | Primary. individual interviews conducted | tertiary | Health Grouping/Profiling - Assess differential risk of diabetic related complications across groups - Assess non-diabetes metabolic derangements (e.g. lipid, blood pressure) across groups - ASsess differential risk of psychological outcomes across groups | 1. Canadian occupational performance measure (COPM) average performance | Judgemental splitting by patients' clinical characteristics | SPSS statistical package  version 19.0 | 2 | 1. Canadian Occupational Performance Measure average performance >= 5 points  2. Canadian Occupational Performance Measure average performance < 4 points |
| **S/No** | **Authors (Year)** | **Article Name** | **Continent** | **Country** | **Study design** | **Sample size** | **T2DM Patient population** | **Mean / Median Age of patients** | **Gender [Male (%)]** | **Duration of diabetes** | **Data source** | **Study setting** | **Objective of segmentation** | **Segmentation variables** | **Statistical methods used** | **Software** | **Number of segments** | **Categories of segments** |
| 89 | Moehlecke M et al (2010) | Effect of metabolic syndrome and of its individual components on renal function of patients with type 2 diabetes mellitus. | South America | Brazil | Cross-sectional study | 842 | Adult T2DM patients over 35 years and without the use of insulin during the first 5 years after diagnosis | Expressed as Mean(SD) 1. Group 1 (no MetS): 55.0 (10.7) 2. Group 2 (no MetS): 56.9 (9.7) 3. Group 3: 58.4 (10.2) 4. Group 4: 57.7 (10.0) 5. Group 5: 58.9 (10.1) | 1. Group 1 (no MetS): 29 (70.7%) 2. Group 2 (no MetS): 82 (59.0%) 3. Group 3: 84 (38.2%) 4. Group 4: 74 (28.7%) 5. Group 5: 44 (23.9%) | Expressed as Mean(SD) 1. Group 1 (no MetS): 9.2 (7.5) 2. Group 2 (no MetS): 10.8 (7.7) 3. Group 3: 11.6 (8.6) 4. Group 4: 10.6 (8.1) 5. Group 5: 11.1 (8.2) | Primary  Clinical and laboratory evaluation including estimated eGFR was performed. Patients were interviewed | tertiary | 1) Health Grouping/Profiling 2) Assess differential risk of diabetic related complications across groups | 1. Number of metabolic syndrome (MetS) components | Judgemental splitting by patients' clinical characteristics | not specified | 5 | 1. Group 1 (T2DM with no metabolic syndrome component) 2. Group 2 (T2DM with 2 metabolic syndrome components) 3. Group 3 (T2DM with 3 metabolic syndrome components) 4. Group 4 (T2DM with 4 metabolic syndrome components) 5. Group 5 (T2DM with 5 metabolic syndrome components) |
| 90 | Mohan V et al (2013) | Clinical profile of long-term survivors and nonsurvivors with type 2 diabetes. | Asia | India | Cross-sectional study | 545 | T2DM patients who survived >= 40 years of documented duration of diabetes and non-survivors T2DM patients | Expressed as Mean(SD) Age at time of death 1. Long-term T2DM survivors: 76.7 (6.9) 2. T2DM non-survivors: 56.3 +/- 11.7  Age at diagnosis 1. Long-term T2DM survivors: 33.5 (6.9) 2. T2DM non-survivors: 34 (5.5) | 1. Long-term T2DM survivors: 196 (82.4%) 2. T2DM non-survivors: 253 (82.1%) | 1. Long-term T2DM survivors: 43.7 +/- 3.9 2. T2DM non-survivors (at time of death): 22.4 +/- 11.0 | -Secondary - Dr Mohan's diabetes specialities clinic in Chennai City Electronic database | tertiary | - Health Grouping/Profiling | 1. Survival of >= 40 years of documented diabetes | Judgemental splitting by patients' clinical characteristics | spss for windows version 15.0 | 2 | 1. Long-term T2DM survivors 2. T2DM non-survivors |
| 91 | Morris NS et al (2006) | Literacy and health outcomes: a cross-sectional study in 1002 adults with diabetes. | North America | United States of America | Cross-sectional study | 1002 | Adult T2DM patients who are English speaking from the Vermont Diabetes Information System, a diabetes decision support system in a region-wide sample of primary care practices | Expressed in Median (IQR) All Subjects: 66 (57–74)  1. Inadequate Literacy: 74 (67–79)  2. Marginal Literacy: 74 (67–79)  3. Adequate Literacy: 64 (56–72) | All Subjects: 457 (46%)  1. Inadequate Literacy: 54 (51%) 2. Marginal Literacy: 32 (48%) 3. Adequate Literacy: 371 (45%) | Expresed in Median (IQR) All Subjects: 6.8 (3–14) 1. Inadequate Literacy: 9.5 (4–20) 2. Marginal Literacy: 10.5 (4–20) 3. Adequate Literacy: 6.3 (3–13) | Secondary - Vermont Diabetes Information System. Primary - interview | primary | Assess differential risk of diabetic related complications across groups - Assess non-diabetes metabolic derangements (e.g. lipid, blood pressure) across groups - Assess diabetic control across groups | 1. Level of literacy | Judgemental Splitting by patients' sociodemographic and/or economic attributes | STATA 8.2 | 3 | 1. Inadequate health literacy (0–16 correct answers) 2. Marginal health literacy (17–22 correct answers)  3. Adequate health literacy (23–36 correct answers) |
| 92 | Muggeo M et al (2000) | Fasting Plasma Glucose Variability Predicts 10-Year Survival of Type 2 Diabetic Patients | Europe | Italy | Retrospective cohort study | 1409 | Adult T2DM patients | Expressed as Mean(SD) 66.4 (5.1) | 652 (46.3%) | Expressed as Mean(SD) 12.3 (6.3) | Primary  Questionnaires and blood tests | Tertiary | Assess mortality | 1. Mean fasting plasma glucose 2. Coefficient of variation for fasting plasma glucose | Judgemental splitting by patients' clinical characteristics | SPSS and EGRET | 1) Tertiles of mean FPG: 3 2) Tertiles of coefficient of variation for fasting plasma glucose: 3 | Tertiles of mean FPG 1. Tertile I 2. Tertile 2 3. Tertile 3  Tertiles of coefficient of variation FPG 1. Tertile I 2. Tertile 2 3. Tertile 3 |
| **S/No** | **Authors (Year)** | **Article Name** | **Continent** | **Country** | **Study design** | **Sample size** | **T2DM Patient population** | **Mean / Median Age of patients** | **Gender [Male (%)]** | **Duration of diabetes** | **Data source** | **Study setting** | **Objective of segmentation** | **Segmentation variables** | **Statistical methods used** | **Software** | **Number of segments** | **Categories of segments** |
| 93 | Nakhjavani M et al (2012) | Gender difference in albuminuria and ischemic heart disease in type 2 diabetes. | Asia | Iran | Cross-sectional study | 926 | Adult T2DM patients admitted to Vali Asr hospital | Expressed in Mean(SD) 1. With Albuminuria: 59.64 (2.80)  2, Without Albuminuria: 56.27 (0.549) | 1. With Albuminuria: 367 (39.6%) 2. Without Albuminuria: 429 (46.3%) | Expressed in Mean(SD) 1. With Albuminuria: 10.03 (0.42) 2. Without Albuminuria: 7.94 (0.43) | Secondary  Patient admission records from Vali Asr Hospital | tertiary | - Assess differential risk of diabetic related complications across groups  - Assess non-diabetes metabolic derangements (e.g. lipid, blood pressure) across groups | 1. Presence of Albuminuria | Judgemental splitting by patients' clinical characteristics | SPSS 16 for windows | 2 | 1. With Albuminuria  2. Without Albuminuria |
| 94 | Nefs G et al (2015) | Depressive Symptom Clusters Differentially Predict Cardiovascular Hospitalization in People With Type 2 Diabetes. | Europe | Netherlands | Prospective record linkage study | 1465 | T2DM patients seen in primary care in the netherlands | Expressed as Mean(SD) 67 (10) | 718 (49%) | 60% of participants reported having diabetes for 3 or more years | -Secondary -Data from the DiaDDZoB (diabetes, depression, type D personality Zuidoos-brabant study) | primary | - Health Grouping/Profiling - Assess differential risk of diabetic related complications across groups - Assess non-diabetes metabolic derangements (e.g. lipid, blood pressure) across groups - Assess differential healthcare utilization | 1. Presence of depressive symptoms (dysphoria and anhedonia) and anxiety | Cluster analysis | PASW statistics version 19 | 3 | 1. Dysphoria 2. Anhedonia 3. Anxiety |
| 95 | Nunes S et al (2019) | Three different phenotypes of mild nonproliferative diabetic retinopathy with different risks for development of clinically significant macular edema | Europe | Portugal | Prospective cohort study | 376 | Adult patients diagnosed adult-onset type 2 diabetes, age 40 to 75 years, mild NPDR (20 and 35 of ETDRS classification), best corrected visual acuity (BCVA) as tested in the ETDRS6 of 80 or higher ETDRS letters score (Snellen equivalent ‡20/25) and refraction with a spherical equivalent less than 65 diopters (D) | Expressed as Median (IQR) 62.0 (55.0–67.0) | 259 (63.2%) | Expressed as Median (IQR) 10.0 (6.0–14.0) | primary. Laboratory analyses were performed at baseline (V0) and at the 6-month (V6) and at the 24-month (V24) visits or at the  pretreatment visit. | tertiary | Assess differential risk of diabetic related complications across groups | 1. Microaneurysm (MA) turnover and for the central retinal thickness (RT) | Cluster analysis | STATA software version 12.1 | 3 | 1. Phenotype A (Low MA turnover and normal RT) (48.1%) 2. Phenotype B (Low MA turnover and increased central RT) (23.2%) 3. Phenotype C (High MA turnover) (28.7%) |
| 96 | O’Donnell A et al (2015) | Neighborhood social environment and patterns of depressive symptoms among patients with type 2 diabetes mellitus. | North America | United States of America | Cross-sectional study | 179 | Adult T2DM patients 1) aged 30 years and older, 2) a diagnosis of type 2 DM and a current prescription for an oral hypoglycemic agent and 3) a prescription for an antidepressant during the period April 2010 to April 2011 | Expressed in Mean (SD) 1. Persistent depressive symptoms: 58.9 (7.9)  2. New depressive symptoms: 60.3 (7.9) 3. Remitted depressive symptoms: 56.0 (10.3) 4. No or few depressive symptoms: 57.0 (11.4) | 1. Persistent depressive symptoms: 9 (26.2%) 2. New depressive symptoms: 12 (30.8%) 3. Remitted depressive symptoms: 29 (34.1%) 4. No or few depressive symptoms: 6 (46.2%) | not specified | primary, clinic electronic medical records, self administered PHQ-9 at baseline and 12 weeks, self administered questionnaire, MMSE measurement | primary | Assess psychological symptoms across groups | 1. Patterns of depressive symptoms | Judgemental splitting by patients' clinical characteristics | STATA version 12 for Windows | 4 | 1. Persistent depressive symptoms 2. New depressive symptoms 3. Remitted depressive symptoms  4. No or few depressive symptoms |
| **S/No** | **Authors (Year)** | **Article Name** | **Continent** | **Country** | **Study design** | **Sample size** | **T2DM Patient population** | **Mean / Median Age of patients** | **Gender [Male (%)]** | **Duration of diabetes** | **Data source** | **Study setting** | **Objective of segmentation** | **Segmentation variables** | **Statistical methods used** | **Software** | **Number of segments** | **Categories of segments** |
| 97 | Ogihara T et al (2017) | Relationships between lifestyle patterns and cardio-renal-metabolic parameters in patients with type 2 diabetes mellitus: A cross-sectional study. | Asia | Japan | Cross-sectional study | 726 | Adult T2DM Japanese outpatients free of history of cardiovascular diseases | Expressed in Mean(SD) 57.8 (8.6) | 456 (62.9%) | Expressed as Mean(SD) 9.9 (7.2) | primary. self-administered questionnaires, Biochemical tests, Measurement of baPWV | tertiary | - Assess differential risk of diabetic related complications across groups  - Assess non-diabetes metabolic derangements (e.g. lipid, blood pressure) across groups | Lifestyle patterns 1. Type 1 pattern: Morningness-eveningness, sleep quality and depressive status 2. Type 2 pattern: Consumption of food, alcohol and cigarettes 3. Type 3 pattern: Physical activity | Factor analysis | SAS software version 9.3 | 1. Type 1 pattern: Morningness-eveningness, sleep quality and depressive status: 5  2. Type 2 pattern: Consumption of food, alcohol and cigarettes: 5  3. Type 3 pattern: Physical activity: 5 | Type 1 pattern: Morningness-eveningness, sleep quality and depressive status  1. Quintile 1 2. Quintile 2 3. Quintile 3 4. Quintile 4 5. Quintile 5  Type 2 pattern: Consumption of food, alcohol and cigarettes  1. Quintile 1  2. Quintile 2  3. Quintile 3  4. Quintile 4  5. Quintile 5  Type 3 pattern: Physical activity 1. Quintile 1  2. Quintile 2  3. Quintile 3  4. Quintile 4  5. Quintile 5 |
| 98 | Okosun IS et al (2014) | Clustering of cardiometabolic risk factors and risk of elevated HbA1c in non-Hispanic White, non-Hispanic Black and Mexican-American adults with type 2 diabetes. | North America | United States of America | Cross-sectional study | 2910 | Adults 18 years and older (Non-hispanic whites (NHW), Non-hispanic black (NHB) and Mexican-American), who havediagnosed and undiagnosed T2DM and with values for the following variables: sex, age, income, education, marital status, race/ethnicity, waist circumference, height, weight, and fasting blood glucose (FGP), oral glucose tolerance test (OGTT), triglycerides, high-density lipoprotein (HDL), low-density lipoprotein (LDL), blood pressure (BP), and HbA1c | Not specified | All: 55.7 1. Non-Hispanic White: 57 2. Non-Hispanic Black: 44 3. Mexican-American: 60.8 | Not specified | Secondary Retrospective data from the united states national health and nutritional examination surveys (NHANES) | Not applicable | Assess non-diabetes metabolic derangements (e.g. lipid, blood pressure) across groups Assess diabetic control across groups | 1. Patient ethnicity | Judgemental Splitting by patients' sociodemographic and/or economic attributes | SAS for windows version 9.2 and SAS callable SUDAAN | 3 | 1. Non-Hispanic White 2. Non-Hispanic Black 3. Mexican-African |
| **S/No** | **Authors (Year)** | **Article Name** | **Continent** | **Country** | **Study design** | **Sample size** | **T2DM Patient population** | **Mean / Median Age of patients** | **Gender [Male (%)]** | **Duration of diabetes** | **Data source** | **Study setting** | **Objective of segmentation** | **Segmentation variables** | **Statistical methods used** | **Software** | **Number of segments** | **Categories of segments** |
| 99 | Osborn CY et al (2010) | Racial disparities in the treatment of depression in low-income persons with diabetes. | North America | United States of America | Cross-sectional study | 69068 | Adult T2DM patients aged between 40 and 79 years old, to speak English, and to not have been treated for cancer | Expressed in Mean(SD) Total: 54.5 (9.0)  Expressed in N(%) 1. White only 40-49 years: 1072 (30.2%) 50-59 years: 1304 (36.7%) 60-69 years: 919 (25.9%) 70-79 years: 257 (7.2%)  2. AFrican American only 40-49 years: 3469 (34.1%) 50-59 years: 3954 (38.9%) 60-69 years: 2046 (20.1%) 70-79 years: 695 (6.8%)  3. Others/mixed race 40-49 years: 176 (31.3%)  50-59 years: 235 (41.7%) 60-69 years: 119 (21.1%) 70-79 years: 33 (5.9%) | 1, White only: 1136 (32.0%) 2. African American: 3419 (33.6%) 3. Others/ mixed race: 205 (36.4%) | not specified | Primary. baseline interview contained questions on demographic information, medical history, family history, environmental and occupational exposures, diet, physical activity, and psychosocial variables | primary | Assess differential healthcare utilization | 1. Patients' race | Judgemental Splitting by patients' sociodemographic and/or economic attributes | SPSS version 17.0 | 3 | 1. White 2. African American 3. Others/mixed race |
| 100 | Penno G et al (2013) | Gender differences in cardiovascular disease risk factors, treatments and complications in patients with type 2 diabetes: the RIACE Italian multicentre study. | Europe | Italy | Cross-sectional study | 15773 | Consecutive patients with type 2 diabetes from the Renal Insufﬁciency And Cardiovascular Events (RIACE) Italian multicentre study, attending 19 hospital-based diabetes clinics in 2007–2008 | Expressed in Median (IQR) Total: 67 (59–73) 1. Men: 66 (59–72)  2. Women: 68 (60–75) | 8960 (56.8%) | Expressed in Median (IQR) Total: 11 (5–20) 1. Men: 10 (5-20) 2. Women: 11 (5-20) | Secondary. Data collected at the baseline visit for the Renal Insufﬁciency and Cardiovascular Events (RIACE) Italian multicentre study were used in the present analysis. | Tertiary | - Assess differential risk of diabetic related complications across groups  - Assess non-diabetes metabolic derangements (e.g. lipid, blood pressure) across groups | 1. Patient's gender | Judgemental Splitting by patients' sociodemographic and/or economic attributes | SPSS version 13.0 | 2 | 1. Men 2. Women |
| **S/No** | **Authors (Year)** | **Article Name** | **Continent** | **Country** | **Study design** | **Sample size** | **T2DM Patient population** | **Mean / Median Age of patients** | **Gender [Male (%)]** | **Duration of diabetes** | **Data source** | **Study setting** | **Objective of segmentation** | **Segmentation variables** | **Statistical methods used** | **Software** | **Number of segments** | **Categories of segments** |
| 101 | Penno G et al (2013) | HbA1c variability as an independent correlate of nephropathy, but not retinopathy, in patients with type 2 diabetes: the Renal Insufficiency And Cardiovascular Events (RIACE) Italian multicenter study. | Europe | Italy | Cross-sectional study | 8260 | Adult Caucasian T2DM patients attending 19 hospital-based diabetes clinics of the National Health Service throughout Italy in years 2007–2008. | Expressed in Mean(SD) HbA1c-MEAN below  1. HbA1c-SD below: 67.4 (9.7)  2. HbA1c-SD above: 66.2 (10.1)    HbA1c-MEAN above  1. HbA1c-SD below: 69.1 (9.3)  2. HbA1c-SD above: 66.6 (10.2) | HbA1c-MEAN below  1. HbA1c-SD below: 1627 (58.5%) 2. HbA1c-SD above: 836 (61.2%)   HbA1c-MEAN above  1. HbA1c-SD below: 677 (49.6%) 2. HbA1c-SD above: 1586 (57.1%) | HbA1c-MEAN below  1. HbA1c-SD below: 14.0 (10.2)  2. HbA1c-SD above: 12.0 (9.6)   HbA1c-MEAN above  1. HbA1c-SD below: 19.4 (9.6) 2. HbA1c-SD above: 16.6 (10.0) | Secondary. Data collected at the baseline visit for the RIACE Italian Multicenter Study | tertiary | Assess differential risk of diabetic related complications across groups | 1. HbA1c variability | Latent class analysis | SPSS 13.0 software | 4 | 1. HbA1c-Mean below and HbA1c-SD below 2. Hba1c-Mean below and Hba1c-SD above 3. Hba1c-Mean above and Hba1c-SD below 4. Hba1c-Mean above and Hba1c-SD above  Abbreviations: Hba1c-Mean - Intra-individual variability in Hba1c, Hba1c-SD - Standard deviation of Hba1c |
| 102 | Prentice JC et al (2012) | Primary care and health outcomes among older patients with diabetes. | North America | United States of America | Retrospective cohort study | 116292 | All T2DM veterans diagnosed with diabetes before 2001, with a prescription for diabetes medication in the current year and/or 2+ visits or stays with a diabetes code for inpatient and/or outpatient visits | Expressed as N <65 years old: 36088  65-69: 27042  70-74: 22790  75-80: 18946  >80: 11426  Total sample: 116292 | <65 years old: 35186 (97.5%)  65-69: 26663 (98.6%)  70-74: 22585 (99.1%) 75-80: 18567 (98.0%)  >80: 11163 (97.7%)  Total sample: 114199 (98.2% | not specified | Secondary data from VA administrative databases and Medicare claims. | primary | Assess differential healthcare utilization | 1. Patient age | Judgemental Splitting by patients' sociodemographic and/or economic attributes | STATA 10.0. | 5 | 1. Age <65 years old 2. Age between 65 and 69, 3. Age between 70 and 74 4. Age between 75 and 80 5. Age>80 years old |
| **S/No** | **Authors (Year)** | **Article Name** | **Continent** | **Country** | **Study design** | **Sample size** | **T2DM Patient population** | **Mean / Median Age of patients** | **Gender [Male (%)]** | **Duration of diabetes** | **Data source** | **Study setting** | **Objective of segmentation** | **Segmentation variables** | **Statistical methods used** | **Software** | **Number of segments** | **Categories of segments** |
| 103 | Rabi DM et al (2007) | Clinical and medication profiles stratified by household income in patients referred for diabetes care. | North America | Canada | Cross-sectional study | 4687 | T2DM patients who go to the Diabetes Education Centre in Clgary, ALberta | Expressed as Median (IQR) 1. Income quintile 1, less than $40877: 56.95 (22.9) 2. Income quintile 2, $40878 – $53065: 56.52 (21.48) 3. Income quintile 3, $53066 – $62921: 56.95 (19.94) 4. Income quintile 4, $62922 – 79828: 55.24 (19.44) 5. Income quintile 5, more than $7982: 55.27 (18.5) | Not specified | Expressed in Median (IQR) 1. Income quintile 1, less than $40877: 4 (9) 2. Income quintile 2, $40878 – $53065: 3 (8) 3. Income quintile 3, $53066 – $62921: 3 (10) 4. Income quintile 4, $62922 – 79828: 3 (9) 5. Income quintile 5, more than $7982: 3 (7) | -Secondary  -Statistics Canada Census Data (2001) for neighbourhood income data -Primary Data - Referral form containing patient's clinical data (HbA1c, serum lipid profiles, microalbumin, height and weight) which was completed by the referring physician | -Tertiary | - Health Grouping/Profiling  - Assess differential healthcare utilization | 1. Household income | Judgemental Splitting by patients' sociodemographic and/or economic attributes | STATA Version 8 | 5 | 1. Income quintile 1, less than $40877 2. Income quintile 2, $40878 – $53065 3. Income quintile 3, $53066 – $62921 4. Income quintile 4, $62922 – 79828 5. Income quintile 5, more than $79829 |
| 104 | Ravera M et al (2009) | Chronic kidney disease and cardiovascular risk in hypertensive type 2 diabetics: a primary care perspective. | Europe | Italy | Cross-sectional study | 7582 | Hypertensive type 2 diabetics (age 25–89 years old) who visited the GP in year 2005 who had serum creatinine data | Expressed in mean (SD) 1. GFR <60: 75 (8)  2. GFR >= 60: 67 (10) | 1. GFR <60: 643 (33%) 2. GFR >= 60: 2873 (51%) | not specified | -Secondary -Health Search Database (HSD) | primary | 1) Assess differential risk of diabetic related complications across groups 2) Assess non-diabetes metabolic derangements (e.g. lipid, blood pressure) across groups | 1. Patients' glomerular filtration rate (GFR) | Judgemental splitting by patients' clinical characteristics | -SPSS version 13.0 | 2 | 1. GFR <60 2. GFR >= 60 |
| 105 | Ravona-Springer R et al (2014) | Trajectories in Glycemic Control over Time Are Associated with Cognitive Performance in Elderly Subjects with Type 2 Diabetes | Asia | Israel | Retrospective cohort study | 835 | Elderly (≥65 years old) with T2DM, cognitively normal | Expressed as Mean(SD) Overall: 72.75 (4.63) 1. Lower stable: 72.99 (4.75) 2. Higher stable: 72.91 (4.66) 3. Lower Increasing: 72.52 (4.46) 4. Higher Increasing: 70.78 (4.16) 5. Lower Decreasing: 73.63 (4.48) 6. Higher Decreasing: 69.73 (3.35) | 501 (60%) | Not specified | Secondary  Health administrative database | not applicable | Assess cognitive related outcomes across groups | 1. Long term trajectory of Hba1c | Latent class analysis | SAS | 6 | 1. Lower stable 2. Higher stable 3. Lower increasing 4. Higher increasing 5. Lower decreasing 6. Higher decreasing |
| **S/No** | **Authors (Year)** | **Article Name** | **Continent** | **Country** | **Study design** | **Sample size** | **T2DM Patient population** | **Mean / Median Age of patients** | **Gender [Male (%)]** | **Duration of diabetes** | **Data source** | **Study setting** | **Objective of segmentation** | **Segmentation variables** | **Statistical methods used** | **Software** | **Number of segments** | **Categories of segments** |
| 106 | Rosa MQM et al (2017) | Disease and Economic Burden of Hospitalizations Attributable to Diabetes Mellitus and Its Complications: A Nationwide Study in Brazil. | Europe | Brazil | Cross-sectional study | 313273 | Adult T2DM patients aged 20 years and older in Brazil with hospitalisation for T2DM related hospitalizations | not specified | 148404 (47.4%) | not specified | -Secondary -Hospitalization and cost data from the National Hospitalization Information System (SIH) -PRevalence of self-reported diabetes obtained from the 2013 NHS | tertiary | - Assess differential healthcare utilization | 1. Patients' age | Judgemental Splitting by patients' sociodemographic and/or economic attributes | Microsoft Excel (2007) | 4 | 1. Age 20–44 years old  2. Age 45–64 years old 3. Age 65–74 years old 4. Age 75+ years old |
| 107 | Safai N et al (2018) | Stratification of type 2 diabetes based on routine clinical markers | Europe | Denmark | Cross-sectional study | 2290 | Adult T2DM patients referred to a short term structured treatment program providing self-management tools, professional support for behavioral changes, and multifactorial treatment and risk factor evaluation and intervention | Expressed as Mean(SD) All: 59.4 (12.2)  1: 56.2 (11.7)  2: 56.8 (11.7)  3: 57.1 (12.1)  4: 66.2 (10.1) 5: 54.8 (11.4) | All: 1393 (61%)  1: 33 (51%)  2: 301 (61%) 3: 318 (62%) 4: 444 (61%) 5: 298 (60% | Expressed in Mean(SD) All: 7.9 (7.0)  1: 6.9 (8.8)  2: 0.9 (0.6) 3: 13.2 (7.8) 4: 9.3 (5.4) 5: 7.4 (5.6) | Secondary. Demographic,c linical, and laboratory data from the electronic medical records and laboratory database from Steno Diabetes Center  Copenhagen | tertiary | - Health Grouping/Profiling | 1. HbA1c level 2. Age at T2DM diagnosis 3. Diabetes duration 4. Patients' BMI 5. HOMA2-IR, HOMA2-β, and GAD65 autoantibody titre | cluster analysis | SAS software  (version 9.4). | 5 | 1. Autoimmune b-cell failure (3%) 2. Insulin resistance with short disease duration (21%)  3. Non-autoimmune b-cell failure (22%)  4. Insulin resistance with long disease duration (32%) 5. Presence of metabolic syndrome (22%) |
| 108 | Sakurai T et al (2014) | Differential subtypes of diabetic older adults diagnosed with Alzheimer's disease. | Asia | Japan | Cross-sectional study | 91 | Elderly T2DM patients aged between 65 and 85 years who had been diagnosed with AD and treated in the NCGG (National Center for Geriatrics and Gerontology) | Expressed in Mean (SD) 1. Diabetes total: 76.8 (5.0) 2. Non-diabetes: 77.1 (5.3) 3. Diabetes HbA1c <7.0%: 76.9 (4.7) 4. Diabetes HbA1c >/= 7.0%: 76.7 (5.3) | 1. Diabetes total: 42 (46.2%) 2. Non-diabetes: 40 (24.8%) 3. Diabetes HbA1c <7.0%: 21 (53.8%) 4. Diabetes HbA1c >/= 7.0%: 21( 40.4%) | not specified | -primary -Comprehensive patient assessment, laboratory measurements, neuroimaging studies | tertiary | - Health Grouping/Profiling | 1. Patients' HbA1c level (>/= 7.0 or <7.0) | Judgemental splitting by patients' clinical characteristics | SPSS 19.0 for windows | 2 | 1. Diabetes, HbA1c <7% 2. Diabetes, HbA1c >= 7% |
| 109 | Sancho-Mestre C et al (2016) | Pharmaceutical cost and multimorbidity with type 2 diabetes mellitus using electronic health record data. | Europe | Spain | Cross-sectional study | 350,015 | Adult T2DM patients being treated with antidiabetics and/or blood glucose reagent strips | Expressed as Mean 67.4 | 181658 (51.9%) | not specified | Secondary, data from the Electronic Health Record (EHR) and the Electronic Prescriber system (GAIA) | not specified | Assess differential healthcare utilization - Assess differential risk of diabetic related complications across groups - Assess non-diabetes metabolic derangements (e.g. lipid, blood pressure) across groups | 1. Clinical Risk Groups (CRG) patient grouping system | Judgemental splitting by patients' clinical characteristics | SPSS version 20.0 | 6 | 1. Clinical Risk Groups 1 to 4  2. Clinical Risk Group 5 3. Clinical Risk Group 6 4. Clinical Risk Group 7  5. Clinical Risk Group 8  6. Clinical Risk Group 9 |
| **S/No** | **Authors (Year)** | **Article Name** | **Continent** | **Country** | **Study design** | **Sample size** | **T2DM Patient population** | **Mean / Median Age of patients** | **Gender [Male (%)]** | **Duration of diabetes** | **Data source** | **Study setting** | **Objective of segmentation** | **Segmentation variables** | **Statistical methods used** | **Software** | **Number of segments** | **Categories of segments** |
| 110 | Sarmento RA et al (2018) | Eating patterns and health outcomes in patients with type 2 diabetes | South America | Brazil | Cross-sectional study | 197 | Adult T2DM patients between 30 and 80 years of age at onset of diabetes, with no previous episode of ketoacidosis or dumented keonuria who had not been using insulin in the 5 years since diagnosis | Expressed as Mean(SD) 1. Unhealthy: 61.1 (9.0) 2. Healthy: 63.9 (9.1) | 1. Unhealthy: 44 (44%) 2. Healthy: 28 (28.9%) | Expressed as Median (IQR) 1. Unhealthy: 10 (4.0 - 17.7 ) 2. Healthy:10 (5.0 - 19.5) | Primary.  Clincal, laboratory, nutritional evaluations, quantitative food frequency questionnaire and eating patterns | tertiary | 1) Health Grouping/Profiling 2) Assess diabetic control across groups | 1. Dietary patterns | Cluster analysis | SPSS version 20.0 | 2 | 1. Unhealthy eating patterns 2. Healthy eating patterns |
| 111 | Schäfer I et al (2013) | Identifying groups of nonparticipants in type 2 diabetes mellitus education. | Europe | Germany | Cross-sectional study | 297 | Adult T2DM patients aged < 80 years old | Expressed in Mean(SD) 1. Cluster 1: 70 (9) 2. Cluster 2: 67 (8) 3. Cluster 3: 65 (12) 4. Cluster 4: 66 (10) | 1. Cluster 1: 23 (59%) 2. Cluster 2: 13 (59%) 3. Cluster 3: 29 (58%) 4. Cluster 4: 7 (50%) | Expressed as Mean(SD) 1. Cluster 1: 8.6 (8.0) 2. Cluster 2: 8.2 (6.1) 3. Cluster 3: 8.4 (8.4) 4. Cluster 4: 6.5 (5.1) | primary. standardized postal patient survey and chart review | primary | Health Grouping/Profiling | 1. Reasons for not participating in diabetes education program | Cluster analysis | S-PLUS 8.1 and Stata 11.0 | 4 | 1. Cluster 1 “Informed and responsible”  2. Cluster 2 “Unconcerned without desire for more information”  3. Cluster 3 “Uninformed but responsible”  4. Cluster 4 “Anxious and burdened” |
| 112 | Schillinger D et al (2002) | Association of health literacy with diabetes outcomes. | North America | United States of America | Cross-sectional study | 408 | English or Spanish speaking age >30 and had T2DM identified from a clinical database with a recorded visit in the prior 12 months and at least 1 additional visit with the same physician within the prior 6 months | Expressed as mean (SD) Total: 58.1 (11.4)  1. Inadequate health Literacy: 62.7 (10.9) 2. Marginal Health literacy : 59.8 (9.8) 3. Adequate health literacy: 54.0 (10.7) | All: 42 Total: 173 (42) 1. Inadequate health Literacy: 52 (33%) 2. Marginal Health literacy : 24 (44%) 3. Adequate health literacy: 97 (49%) | Expressed in Mean (SD)  All: 9.5 (8.0%) 1. Inadequate health Literacy: 11.4 (8.7%) 2. Marginal Health literacy : 10.4 (8.3%) 3. Adequate health literacy: 7.7 (6.9%) | - Primary and Secondary - Clinical Database of 2 primary care clinics, orally administered questionnaire and participant's health literacy scores (assessed using short-form Test of Functional Health Literacy (s-TOFHLA) ). | Primary | 1) Assess differential risk of diabetic related complications across groups  2) Assess non-diabetes metabolic derangements (e.g. lipid, blood pressure) across groups 3) Assess diabetic control across groups | 1. Health Literacy Level | Judgemental Splitting by patients' sociodemographic and/or economic attributes | SAS version 8 | 3 | 1. Inadequate health Literacy (s-TOFHLA score 0-16)  2. Marginal Health literacy (s-TOFHLA score 17-22) 3. Adequate health literacy (s-TOFHLA score 23-36) |
| **S/No** | **Authors (Year)** | **Article Name** | **Continent** | **Country** | **Study design** | **Sample size** | **T2DM Patient population** | **Mean / Median Age of patients** | **Gender [Male (%)]** | **Duration of diabetes** | **Data source** | **Study setting** | **Objective of segmentation** | **Segmentation variables** | **Statistical methods used** | **Software** | **Number of segments** | **Categories of segments** |
| 113 | Seok H at al (2013) | Clinical characteristics and insulin independence of Koreans with new-onset type 2 diabetes presenting with diabetic ketoacidosis. | Asia | Korea | Prospective cohort study | 60 | Age >= 18 years with newly diagnosed diabetes presenting with DKA at 3 university hospitals in Seoul | Expressed as Mean (SD) 1. All patients: 37.1 (13.1)  Type of anti-diabetic treatment 1. On insulin: 33.7 (11.0) 2. Not on insulin and on oral anti-hyperglycemic agents: 37.2 (15.4) 3. Not on insulin and oral anti-hyperglycemic agents: 36.9 (14.5)  Characteristics of T2DM 1. Ketosis prone T2DM: 37.1 (13.7) 2. Previously diagnosed T2DM with diabetic ketoacidosis: 55.9 (11.6) | Total: 65%  Type of anti-diabetic treatment 1. On insulin: 3 (100%) 2. Not on insulin and on oral anti-hyperglycemic agents: 7 (77.8%) 3. Not on insulin and oral anti-hyperglycemic agents: 7 (87.5%)  Characteristics of T2DM 1. Ketosis prone T2DM: 18 (85.7%) 2. Previously diagnosed T2DM with diabetic ketoacidosis: 13 (72.2%) | Expressed as Mean (SD) Characteristics of T2DM 1. Ketosis prone T2DM: - 2. Previously diagnosed T2DM with diabetic ketoacidosis: 9.9 (8.7) years | -Secondary -Data from the three university hospitals and medical records, laboratory data from day of admission | -not specified | -Health grouping/profiling -Assess diabetic control across groups | 1. Type of antidiabetic treatment 2. Characteristics of T2DM | Judgemental splitting by patients' clinical characteristics | Windows version 18.0 | 1. Type of antidiabetic treatment: 3 2. Characteristics of T2DM: 2 | Type of anti-diabetic treatment 1. On insulin 2. Not on insulin and on oral anti-hyperglycemic agents 3. Not on insulin and oral anti-hyperglycemic agents  Characteristics of T2DM 1. Ketosis prone T2DM 3. Previously diagnosed T2DM with diabetic ketoacidosis |
| 114 | Shamshirgaran SM et al (2017) | Age differences in diabetes-related complications and glycemic control. | Asia | Iran | Cross-sectional study | 694 | Age >25 years old and had a caring record in diabetes clinics in two Northwestern provinces of Iran | Expressed as N(%) 1. Age =<59: 454 (69%) 2. Age >=60: 240 (31%) | 1. Age =<59: 117 (26%) 2. Age >=60: 90 (38%) | Expressed as N(%) Age =<59 =<3 years: 97 (22%) 4-7 years: 115 (25%) >=7 years: 240 (53%)  Age >=60 =<3 years: 38 (16%) 4-7 years: 46 (19%) >=7 years: 156 (65%) | - Primary data - Structured questionnaire and clinic records | Primary | 1) Health Grouping/Profiling 2) Assess differential risk of diabetic related complications across groups 3) Assess diabetic control across groups | 1. Patients' age | Judgemental Splitting by patients' sociodemographic and/or economic attributes | SPSS Version 23 | 2 | 1. Age =<59 years old (Younger age) 2. Age >=60 years old (Older age) |
| 115 | Sheu SJ et al (2012) | High HbA1c level was the most important factor associated with prevalence of diabetic retinopathy in Taiwanese type II diabetic patients with a fixed duration. | Asia | Taiwan | Cross-sectional study | 901 | Adult non-insulin dependent T2DM patients enrolled from 2002 to 2009 who had been in the program at least 1year. | Expressed as Mean(SD) 1. No retinopathy: 53.6 (13.4)  2. Retinopathy: 52.3 (12.2) | 1. No retinopathy: 384 (57.2%) 2. Retinopathy: 113 (49.1%) | Expressed as Mean(SD) 1. No retinopathy: 4.8 (5.9)  2. Retinopathy: 9.3 (7.4) | Secondary. information in the data bank of the Diabetes Shared Care System, Kaohsiung Veterans General Hospital, | tertiary | Assess differential risk of diabetic related complications across groups | 1. Presence of diabetic retinopathy | Judgemental splitting by patients' clinical characteristics | SPSS version 12.0 | 2 | 1. No retinopathy 2. Retinopathy |
| **S/No** | **Authors (Year)** | **Article Name** | **Continent** | **Country** | **Study design** | **Sample size** | **T2DM Patient population** | **Mean / Median Age of patients** | **Gender [Male (%)]** | **Duration of diabetes** | **Data source** | **Study setting** | **Objective of segmentation** | **Segmentation variables** | **Statistical methods used** | **Software** | **Number of segments** | **Categories of segments** |
| 116 | Siaw MY et al (2016) | Metabolic parameters in type 2 diabetic patients with varying degrees of glycemic control during Ramadan: An observational study. | Asia | Singapore | Retrospective cohort study | 5,172 | Adult Malay, T2DM patients aged 21 years and older | Total: 60.1 (10.7) 1. Group 1: 55.2 (9.8)  2. Group 2: 59.0 (10.2)  3. Group 3: 62.7 (10.9) | Total: 2006 (38.8%) 1. Group 1: 163 (41.3%) 2. Group 2: 1110 (38.6%) 3. Group 3: 732 (38.6%) | Not specified | Seconday. Chronic Disease Management System (CDMS) of the National Healthcare Group in Singapore | primary and tertiary | Health Grouping/Profiling Assess diabetic control across groups | 1. HbA1c level | Judgemental splitting by patients' clinical characteristics | SPSS for Windows, version 19.0 | 3 | 1. Group 1: HbA1c ≥ 10% 2. Group 2: HbA1c 7.1 – 9.9% 3. Group 3: HbA1c ≤7% |
| 117 | Sidorenkov G et al (2018) | HbA1c response after insulin initiation in patients with type 2 diabetes mellitus in real life practice: Identifying distinct subgroups. | Europe | Netherlands | Retrospective cohort study | 1459 | Adult T2DM patients initiating insulin treatment between January 1, 2007 and December 31, 2013 | All: 65.6 (12.4) 1. Group 1: 60.3 (12.9) 2. Group 2: 66.0 (12.1) 3. Group 3: 66.9 (13.5) | All: 683 (46.8%) 1. Group 1: 58 (48.7%) 2. Group 2: 574 (46.8%) 3. Group 3: 51 (45.1%) | T2DM duration >2 y, n (%) 1. Group 1: 106 (89.1) 2. Group 2: 1044 (85.1) 3. Group 3: 80 (70.8) | Secondary. Groningen Initiative to Analyze Type 2 Diabetes Treatment (GIANTT) database. | primary | Assess treatment outcomes | 1. HbA1c trend | Latent class growth analysis | Mplus 7.4. | 3 | 1. Class 1: Initial a moderate decrease followed by an increase in HbA1c 2 years later, despite receiving more comedication. 2. Class 2: Stable decrease.  3. Class 3: High initial level of HbA1c and a rapid decline within the first year, followed by a slow increase thereafter. |
| 118 | Solini A et al (2014) | Resistant hypertension in patients with type 2 diabetes: clinical correlates and association with complications. | Europe | Italy | Prospective cohort study | 15773 | Adult Caucasian patients not on dialysis or renal transplantation | Expressed in Mean(SD) 1. RH: 69.0 (8.7)  2. NRH1: 65.6 (10.4)  3. NRH 2: 67.5 (9.6) 4. NRH 3: 67.7 (9.7) 5. UH: 66.3 (10.0) | 1. RH: 1246 (52.7%) 2. NRH1: 943 (60.1%)  3. NRH 2: 766 (56.0%) 4. NRH 3: 422 (52.6%) 5. UH: 4201 (56.5%0 | Expressed in Mean(SD) 1. RH: 14.4 (10.2) 2. NRH1: 13.2 (10.2)  3. NRH 2: 13.4 (10.4) 4. NRH 3: 13.3 (9.9) 5. UH: 13.4 (10.3) | secondary. Baseline data from the RIACE Italian Multicenter Study | tertiary | Health Grouping/Profiling - Assess non-diabetes metabolic derangements (e.g. lipid, blood pressure) across groups | 1. Resistant hypertension  2. Number of anti-hypertensive agents | Judgemental splitting by patients' clinical characteristics | SPSS version 13.0 | 5 | 1. Resistant hypertension  2. Controlled non-resistant hypertension on one drug 3. Controlled non-resistant hypertension on two drugs 4. Controlled non-resistant hypertension on three drugs 5. Uncontrolled hypertension with no or less than three drugs |
| 119 | Spauwen PJJ et al (2016) | Lower verbal intelligence is associated with diabetic complications and slower walking speed in people with type 2 diabetes: the Maastricht Study | Europe | Netherlands | Cross-sectional study | 253 | Adult T2DM patients aged 40-75 years old and were living in the Southern part of the Netherland | Expressed as Mean (SD) 1, Low verbal intelligence: 63.7 (6.7) 2. Medium verbal intelligence: 63.7 (7.6) 3. High verbal intelligence: 63.3 (6.6) | 1. Low verbal intelligence: 54 (74.0%) 2. Medium verbal intelligence: 59 (62.8%) 3. High verbal intelligence: 43.4 (80.3%) | Expressed as Median (IQR) 1, Low verbal intelligence: 7.0 (2.5–11.5) 2. Medium verbal intelligence: 4.5 (1.0–10.0)  3. High verbal intelligence: 5.0 (2.0–9.5) | Primary  Questionnaires and survey | Tertiary | Assess differential risk of diabetic related complications across groups Assess physical function across groups Assess non-diabetes metabolic derangements (e.g. lipid, blood pressure) across groups  Assess diabetic control across groups | 1. Verbal intelligence | Judgemental splitting by patients' clinical characteristics | SPSS Version 20 | 3 | 1. Low verbal intelligence 2. Medium verbal intelligence 3. High verbal intelligence |
| **S/No** | **Authors (Year)** | **Article Name** | **Continent** | **Country** | **Study design** | **Sample size** | **T2DM Patient population** | **Mean / Median Age of patients** | **Gender [Male (%)]** | **Duration of diabetes** | **Data source** | **Study setting** | **Objective of segmentation** | **Segmentation variables** | **Statistical methods used** | **Software** | **Number of segments** | **Categories of segments** |
| 120 | Tan ED et al (2016) | Changes in characteristics and management of Asian and Anglo-Celts with type 2 diabetes over a 15-year period in an urban Australian community: The Fremantle Diabetes Study. | Australia | Australia | Cross-sectional study | 1426 | T2DM patients living in a zip code-defined geographic area around the port city of Fremantle in the Australian state of Western Australia | Expressed as Mean(SD) 1. Phase 1: Anglo-Celt: 64.9 (11.4) Asian: 57.4 (11.4)  2. Phase 2: Anglo-Celt: 67.2 (10.6) Asian: 61.1 (10.2) | 1. Phase 1: Anglo-Celt: 389 (48.9%) Asian: 21 (47.7%)  2. Phase 2: Anglo-Celt: 404 (50.9%) Asian: 37 (56.9%) | Expressed as median (IQR) 1. Phase 1: Anglo-Celt: 4.0 (1.0-8.0) Asian: 2.6 (0.6-8.8)  2. Phase 2: Anglo-Celt: 8.0 (2.2-15.0) Asian: 10.0 (3.5-17.5) | -Secondary data -Fremantle Diabetes study Phase I (1993-96) and phase II (2008-11) | -not specified | Health Grouping/Profiling Assess differential risk of diabetic related complications across groups Assess non-diabetes metabolic derangements (e.g. lipid, blood pressure) across groups Assess diabetic control across groups | 1. Race / ethnicity of patients | Judgemental Splitting by patients' sociodemographic and/or economic attributes | SPSS Version 20 | 2 | 1. Phase I of the FDS (1993-96) 2. Phase II of the FDS (2008-11) |
| **S/No** | **Authors (Year)** | **Article Name** | **Continent** | **Country** | **Study design** | **Sample size** | **T2DM Patient population** | **Mean / Median Age of patients** | **Gender [Male (%)]** | **Duration of diabetes** | **Data source** | **Study setting** | **Objective of segmentation** | **Segmentation variables** | **Statistical methods used** | **Software** | **Number of segments** | **Categories of segments** |
| 121 | Tao X et al (2016) | Association between socioeconomic status and metabolic control and diabetes complications: a cross-sectional nationwide study in Chinese adults with type 2 diabetes mellitus. | Asia | China | Cross-sectional study | 25,454 | Age > 18 with a diabetes duration of at least 6 months | Expressed as Mean (SD)  Male: All male patients : 60.9 (12.65) years 1, Educational level: Illiteracy: 70.9 (10.76) Primary Eduation: 67.5 (11.28) Secondary Education: 60.3 (11.88) College and above: 58.2 (13.04)  2. Household income (¥): <2000: 62.1 (11.90) 2000-5000: 61.1 (12.70) >= 5000: 57.9 (13.53)  Female: All female patients: 64 (10.87) years 1. Educational level: Illiteracy: 71.0 (9.34) Primary Eduation: 67.2 (9.91) Secondary Education: 61.3 (10.19) College and above: 61.1 (11.63)  2. Household income (¥): <2000: 64.2 (10.58) 2000-5000: 64.1 (10.83) >= 5000: 62.4(12.13) | All: 11,955 (47.0%)  1. Educational level: Illiteracy: 277 (16.3%) Primary Eduation: 1836 (32.4%) Secondary Education: 5738 (48.1%) College and above: 4104 (67.0%)  2. Household income (¥): <2000: 3941 (39.3%) 2000-5000: 5796 (50.0%) >= 5000: 1804 (60.9%) | Diabetes duration expressed as median (IQR) All patients : 5.8 (2.4-10.9)  Male: 1. Educational level: Illiteracy: 5.8 (2.5 - 11.0) Primary Eduation: 5.7 (2.6 - 11.0) Secondary Education:5.8 (2.5 - 11.0) College and above: 5.8 (2.3 - 10.9)  2. Household income (¥): <2000: 5.5 (2.3 - 10.9) 2000-5000: 5.9 (2.6 - 11.0) >= 5000: 5.8 (2.3 - 10.9)Female:All: 6.8 (2.8 - 12.0)  Female: 1. Educational level: Illiteracy: 7.1 (3.0 - 12.2) Primary Eduation: 7.2 (3.0 - 12.5) Secondary Education: 6.6 (2.8 - 11.8) College and above: 6.7 (2.6 - 12.3)  2. Household income (¥): <2000: 6.6 (2.7 - 11.5)  2000-5000: 6.9 (2.9 - 12.6) >= 5000: 7.0 (2.9 - 12.9) | -Secondary Data -CCMR-3B study (China Cardio Metabolic Registries Nationwide Assessment of Cardiovascular Risk Factors: Blood Glucose, Blood pressure and Blood Lipid in Chinese Patients with Type 2 Diabetes) | Not specified | 1) Health Grouping/Profiling 2) Assess differential risk of diabetic related complications across groups | 1. Education level 2. Household net income | Judgemental Splitting by patients' sociodemographic and/or economic attributes | SAS 9.2 | 1. Education: 4 2. Household net income: 3 | Education  1. Illteracy 2. Primary Education 3. Secondary Education 4. College and above  Household net income  1. <¥2000 2. ¥2000-¥5000 3. >=¥5000) |
| 122 | Toh MPHS et al (2011) | Association of Younger Age With Poor Glycemic and Cholesterol Control in Asians With Type 2 Diabetes Mellitus in Singapore | Asia | Singapore | Cross-sectional study | 58057 | Adult T2DM patients who had at least 2 visits to the public-sector primary care clinic for diabetes treatment | Mean age: 64 (SD:11.6)  Age group n (%) 1. <45 : 2537 (4.3%) 2. 45 - 54: 9712 (16.7%) 3. 55 - 64: 18200 (31.3%) 4. 65 - 74: 16390 (28.2%) 5. 75 - 84: 9245 (15.9%) 6. 85+: 1973 (3.4%) | 26834 (46%) | Not specified | Secondary data Chronic disease registry | Primary | Health grouping / profiling Assess differential risk of diabetic related complication across groups Assess diabetic control across groups | 1. Patients' age | Judgemental Splitting by patients' sociodemographic and/or economic attributes | PASW (Version 18) | 5 | 1. Age <45 years old 2. Age 45 - 54 years old 3. Age 55 - 64 years old 4. Age 75 - 84 years old 5. Age >85 years old |
| **S/No** | **Authors (Year)** | **Article Name** | **Continent** | **Country** | **Study design** | **Sample size** | **T2DM Patient population** | **Mean / Median Age of patients** | **Gender [Male (%)]** | **Duration of diabetes** | **Data source** | **Study setting** | **Objective of segmentation** | **Segmentation variables** | **Statistical methods used** | **Software** | **Number of segments** | **Categories of segments** |
| 123 | Twito O et al (2013) | New-onset diabetes in elderly subjects: association between HbA1c levels, mortality, and coronary revascularization. | Asia | Israel | Retrospective cohort study | 2,994 | Adult T2DM patients from Sharon-Shomron District, Israel who were medically insured by Clalit Health Services (CHS), 65 years of age or older and had at least two blood glucose values of 126 mg/dL and above during the same year | Express as Mean(SD) 1. <6.5%: 76.1 (6.8)  2. 6.5–6.99%: 75.3 (6.6)  3. 7–7.49%: 74.8 (6.1)  4. >/= 7.5%: 74.8 (6.9) | 1. <6.5%: 790 (50%)  2. 6.5–6.99%: 277 (45%) 3. 7–7.49%: 179 (49%) 4. >/= 7.5%: 202 (46%) | not specified | Secondary, computerized database of the Sharon-Shomron District of Clalit Health Services in Israel. | primary | Assess differential risk of diabetic related complications across groups - Assess non-diabetes metabolic derangements (e.g. lipid, blood pressure) across groups - Assess diabetic control across groups - Assess mortality | 1. HbA1c level | Judgemental splitting by patients' clinical characteristics | SPSS statistical software for windows version 20.0 | 4 | 1. HbA1c: < 6.5% (48 mmol/mol) 2. HbA1c: 6.5 - 6.99% (48–52 mmol/mol) 3. HbA1c: 7–7.49% (53–57 mmol/mol) 4. HbA1c >= 7.5% (58 mmol/mol). |
| 124 | Ustulin M et al (2017) | Characteristics of frequent emergency department users with type 2 diabetes mellitus in Korea. | Asia | Korea | Cross-sectional study | 109,412 | T2DM patients in the korean healthcare system who made insurance claims in 2009 with T2DM a a main or secondary diagnosis on arrival at the ED. | Expressed in mean (95% CI) 1. No ED: 62.1 (61.9–62.2)  2. OED: 62.9 (62.6–63.2) 3. FED: 63.1 (62.2–64.1) | 1. No ED: 49,524 (49%)  2. OED: 3,943 (53%) 3. FED: 494 (58%) | not specified | -Secondary -Insurance claims made from 1 Jan 2009 to 31 Dec 2009 from the Health Insurance Review and Assessment Service (HIRA) | -tertiary | - Health Grouping/Profiling - Assess differential healthcare utilization | 1. Frequency of emergency department presentation | Judgemental splitting by patients' clinical characteristics | SAS version 9.3 | 3 | 1. No ED 2. Occasional ED (OED) 3. Frequent ED (FED) |
| **S/No** | **Authors (Year)** | **Article Name** | **Continent** | **Country** | **Study design** | **Sample size** | **T2DM Patient population** | **Mean / Median Age of patients** | **Gender [Male (%)]** | **Duration of diabetes** | **Data source** | **Study setting** | **Objective of segmentation** | **Segmentation variables** | **Statistical methods used** | **Software** | **Number of segments** | **Categories of segments** |
| 125 | van Dijk CE et al (2013) | Type II diabetes patients in primary care: profiles of healthcare utilization obtained from observational data. | Europe | Netherlands | Retrospective cohort study | 6721 | Adult T2DM patients aged 18 years and above who had consulted their GP for T2DM at least once in 2007 and were registered with the practice during the whole year in 2008 | 1. Profile 1 (n=393) 18-34 years old: 0.3% 35-44 years old: 0.8% 45-54 years old: 1.0% 55-64 years old: 3.3% 65-74 years old: 17% >=75 years old: 77.9%  2. Profile 2 (n=3231) 18-34 years old: 1.7% 35-44 years old: 5.9% 45-54 years old: 14% 55-64 years old: 26% 65-74 years old: 26% >=75 years old: 26%  3. Profile 3 (n=3097) 18-34 years old: 0.5% 35-44 years old: 3.7% 45-54 years old: 13% 55-64 years old: 29% 65-74 years old: 31% >=75 years old: 23% | 1. Profile 1 (n=393): 26% 2. Profile 2 (n=3231): 49% 3. Profile 3 (n=3097): 52% | Not specified | - Secondary data - Administrative healthcare records | Primary | - Health Grouping/Profiling - Assess differential healthcare utilization | 1. Type of healthcare utilisation - contacts with general practice, drug prescriptions and referrals to allied healthcare | Latent class analysis | STATA, Mplus and MLwiN. | 3 | 1. Profile 1: high utilisation and frequent home visits 2. Profile 2: low utilisation, GP only 3. Profile 3: high utilisation, GP and nurse |
| 126 | Vepsäläinen T et al (2011) | Physical activity, high-sensitivity C-reactive protein, and total and cardiovascular disease mortality in type 2 diabetes. | Europe | Finland | Prospective cohort study | 569 | Adult T2DM patients aged 45–64 years, who were free of CVD at baseline | Expressed in Mean(SD) 1. 0–4 METs: 58.2 (5.1)  2. >4 METs: 56.5 (5.3) | 1. 0–4 METs: 173 (49.9%) 2. >4 METs: 141 (61.3%) | Expressed in Mean(SD) 1. 0–4 METs:7.9 (4.0) 2. >4 METs: 7.8 (4.0) | Primary. Interview, Medical records, biochemical and symptom assessment, Assessment of physical activity using Metabolic equivalent task (MET) | tertiary | Assess non-diabetes metabolic derangements (e.g. lipid, blood pressure) across groups Assess differential risk of diabetic related complications across groups | 1. Physical activity level assessed according to metabolic equivalent tasks (METs) | Judgemental splitting based on patients' lifestyle habits | SPSS for Windows version 15.0 | 2 | 1. 0–4 metabolic equivalent tasks 2. >4 metabolic equivalent tasks |
| **S/No** | **Authors (Year)** | **Article Name** | **Continent** | **Country** | **Study design** | **Sample size** | **T2DM Patient population** | **Mean / Median Age of patients** | **Gender [Male (%)]** | **Duration of diabetes** | **Data source** | **Study setting** | **Objective of segmentation** | **Segmentation variables** | **Statistical methods used** | **Software** | **Number of segments** | **Categories of segments** |
| 127 | Vitale M et al (2016) | Sex differences in food choices, adherence to dietary recommendations and plasma lipid profile in type 2 diabetes - The TOSCA.IT study. | Europe | Italy | Cross-sectional study | 2573 | Adult T2DM patients aged 50 - 75 years old | Expressed in Mean(SD) Total: 62.1 (6.5) 1. Men: 62.0 (6.5)  2. Women: 62.3 (6.4) | 1535 (59.7%) | Expressed in Mean(SD) Total: 8.5 +/- 5.7  1. Men: 8.4 (5.6)  2. Women: 8.6 (5.8) | primary. baseline anthropometric and clinical data were measured, Use of medications, and dietary habits were assessed | not specified | - Health Grouping/Profiling - Assess non-diabetes metabolic derangements (e.g. lipid, blood pressure) across groups | 1. Patient's gender | Judgemental Splitting by patients' sociodemographic and/or economic attributes | SPSS software for Windows, version19.0. | 2 | 1. Men 2. Women |
| 128 | Walker JJ et al (2011) | Effect of socioeconomic status on mortality among people with type 2 diabetes: a study from the Scottish Diabetes Research Network Epidemiology Group. | Europe | Scotland | Cross-sectional study | 210,994 | Adult T2DM patients between age 35 - 84 in Scotland during 2001 - 2007 | Expressed as Mean(SD) Age of people with T2DM in 2007 1 (least deprived): M: 64.6 (10.7), F: 66.8 (10.9) 2: M: 64.7 (10.8), F: 66.8 (11.0) 3: M: 64.4 (10.9), 66.3 (11.1) 4: M: 64.0 (11.0), F: 66.3 (11.2) 5 (most deprived): M: 62.8 (11.2), F: 65.2 (11.5)   Age at death of people with T2DM in 2001-2007 1 (least deprived): M: 73.2 (8.2), F: 75.2 (7.6)  2: M: 73.1 (8.3), F: 74.5 (7.6)  3: M: 72.2 (8.6), F: 74.0 (8.1)  4: M: 71.8 (8.6), F: 73.4 (8.5)  5 (most deprived): M: 70.3 (9.0), F: 72.5 (8.6) | 1 (least deprived): 15309 (59.9%) 2: 18349 (58.0%) 3: 19962 (55.6%) 4: 21142 (53.3%) 5 (most deprived): 21086 (51.5%) | Expressed as Mean(SD) 1 (least deprived): M: 7.7 (6.8), F: 7.5 (6.7)  2: M: 7.7 (6.8), F: 7.7 (6.8) 3: M: 7.6 (6.6), F: 7.7 (6.6) 4: M: 7.6 (6.6), F: 7.8 (6.6)  5 (most deprived): M: 7.4 (6.3), 7.6 (6.3) | secondary. Population-based data from the Scottish Care Information - diabetes collaboration (SCI-DC) dataset | primary and secondary care | - Assess mortality | 1. Socioeconomic status (SES) of patients using area-based measure of SES assigned to individual people on the basis of where they live by using the Scottish Index of Multiple Deprivation (SIMD) 2006 | Judgemental Splitting by patients' sociodemographic and/or economic attributes | not specified | 5 | 1. Scottish Index of Multiple Deprivation (SIMD) quintile 1 (least deprived) 2. Scottish Index of Multiple Deprivation (SIMD) quintile 2 3. Scottish Index of Multiple Deprivation (SIMD) quintile 3 4. Scottish Index of Multiple Deprivation (SIMD) quintile 4 5. Scottish Index of Multiple Deprivation (SIMD) quintile 5 (most deprived) |
| **S/No** | **Authors (Year)** | **Article Name** | **Continent** | **Country** | **Study design** | **Sample size** | **T2DM Patient population** | **Mean / Median Age of patients** | **Gender [Male (%)]** | **Duration of diabetes** | **Data source** | **Study setting** | **Objective of segmentation** | **Segmentation variables** | **Statistical methods used** | **Software** | **Number of segments** | **Categories of segments** |
| 129 | Walraven I et al (2014) | Distinct HbA1c trajectories in a type 2 diabetes cohort. | Europe | Netherlands | Retrospective cohort study | 5423 | Adult T2DM patients from a managed primary care system | Expressed in Mean (SD) Cohort: 60.6 (11.6)  1. Good glycemic control: 61.2 (11.3)  2. Fast responders: 58.9 (12.3)  3. Insufficient glycemic control: 56.9 (11.7)  4. Bad responders: 55.4 (13.9) | Cohort: 2902 (53.3%) 1. Good glycemic control: 2355 (52.2%) 2. Fast responders: 268 (60.4%) 3. Insufficient glycemic control: 162 (60.4%) 4. Bad responders: 117 (62.6%) | Expressed in Mean (SD) Cohort: 1.0 (0.2–3.6)  1. Good glycemic control: 1.0 (0.2–3.3)  2. Fast responders: 0.3 (0.1–1.3)  3. Insufficient glycemic control: 4.1 (0.9–8.8) 4. Bad responders: 1.4 (0.3–5.7) | Secondary data from the DCS West-Friesland, | primary | Health Grouping/Profiling | 1. HbA1c trends of patients over 9 years | Latent class growth analysis | SPSS 20.0, Mplus 5.21, MLwin 2.25 | 4 | 1. Good glycemic control 2. Fast responders  3. Insufﬁcient glycemic control 4. Bad responders |
| 130 | Wan EY et al (2017) | Association of Visit-to-Visit Variability of Systolic Blood Pressure With Cardiovascular Disease and Mortality in Primary Care Chinese Patients With Type 2 Diabetes-A Retrospective Population-Based Cohort Study. | Asia | Hong Kong | Retrospective cohort study | 124,105 | Age >= 18 years with T2DM without clinical diagnosis of CVD managed in a public general oupatient clinic, with >=5 SBP measurements during the follow up period (24 months after 1st measurement). | Expressed as mean (SD) All: 63.19 (11.26) 1. SD <5 mmHg: 57.52 (10.94) 2. SD >=5 and <7.5 mmHg: 59.40 (10.87) 3. SD >=7.5 and <10 mmHg: 61.28 (11.00) 4. SD >=10 and <12.5 mmHg: 63.01 (10.90) 5. SD >=12.5 and <15 mmHg: 64.65 (10.92) 6. SD >=15 and <17.5 mmHg: 66.29 (10.83) 7. SD >=17.5 and <20 mmHg: 67.34 (10.88) 8. SD >=20 and <22.5 mmHg: 68.28 (10.88) 9. SD >=22.5 and <25 mmHg: 69.00 (10.69) 10. SD >=25 mmHg: 70.50 (10.88) | All: 55103 (44.4%) 1. SD <5 mmHg: 1462 (49.5%) 2. SD >=5 and <7.5 mmHg: 7583 (48.1%) 3. SD >=7.5 and <10 mmHg: 13422 (46.2%) 4. SD >=10 and <12.5 mmHg: 13222 (44.6%) 5. SD >=12.5 and <15 mmHg: 8921 (42.9%) 6. SD >=15 and <17.5 mmHg: 5000 (41.6%) 7. SD >=17.5 and <20 mmHg: 2788 (41.3%) 8. SD >=20 and <22.5 mmHg: 1352 (38.7%) 9. SD >=22.5 and <25 mmHg: 18 (37.8%) 10. SD >=25 mmHg: 674 (37.0%) | Expressed as mean(SD) All: 6.24 (6.48) 1. SD <5 mmHg: 5.34 (5.49) 2. SD >=5 and <7.5 mmHg: 5.74 (6.56) 3. SD >=7.5 and <10 mmHg: 5.91 (6.22) 4. SD >=10 and <12.5 mmHg: 6.23 (6.62)  5. SD >=12.5 and <15 mmHg: 6.51 (6.51) 6. SD >=15 and <17.5 mmHg: 6.67 (6.90) 7. SD >=17.5 and <20 mmHg: 6.84 (7.00) 8. SD >=20 and <22.5 mmHg: 6.84 (7.27) 9. SD >=22.5 and <25 mmHg: 7.10 (7.50) 10. SD >=25 mmHg: 7.27 (7.33) | - Secondary Data - Territory-wide study evaluating effectiveness of a risk assessmennt and management programme for patients with diabetes | Tertiary | 1) Assess differential risk of diabetic related complications across groups 2) Assess non-diabetes metabolic derangements (e.g. lipid, blood pressure) across groups 3) Assess mortality | 1. Visit-to-Visit Variability (VVV) of SBP (mmHg) | Judgemental splitting by patients' clinical characteristics | STATA Version 13.0 | 10 | 1. SD <5 mmHg, 2. SD >=5 and <7.5 mmHg 3. SD >=7.5 and <10 mmHg 4. SD >=10 and <12.5 mmHg  5. SD >=12.5 and <15 mmHg 6. SD >=15 and <17.5 mmHg 7. SD >=17.5 and <20 mmHg 8. SD >=20 and <22.5 mmHg 9. SD >=22.5 and <25 mmHg 10. SD >=25 mmHg. |
| **S/No** | **Authors (Year)** | **Article Name** | **Continent** | **Country** | **Study design** | **Sample size** | **T2DM Patient population** | **Mean / Median Age of patients** | **Gender [Male (%)]** | **Duration of diabetes** | **Data source** | **Study setting** | **Objective of segmentation** | **Segmentation variables** | **Statistical methods used** | **Software** | **Number of segments** | **Categories of segments** |
| 131 | Wang RH et al (2013) | Determinants for quality of life trajectory patterns in patients with type 2 diabetes. | Asia | Taiwan | Prospective cohort study | 466 | Patients (age between 20 and 80 years) diagnosed with T2DM for more than 6 months from 5 diabetic clinics in taiwan and able to read chinese language questionnaires | Expressed as Mean(SD) 1. Steadily poor: 51.50 (16.98) 2. Consistently moderate: 56.87 (11.65)  3. Consistently good: 60.24 (9.95) | 1. Steadily poor: 10 (37.0%) 2. Consistently moderate: 75 (43.1%) 3. Consistently good: 123 (46.4) | 1. Steadily poor 8.68 (6.49)  2. Consistently moderate 10.39 (7.59) 3. Consistently good 9.66 (6.85) | -primary -questionnaire, including Demographic and disease characteristics, biomedical factors, psychosocial factors and QoL were collected at baseline. QoL was further measured every 6 months across four waves after baseline. | primary | - Health Grouping/Profiling - Assess differential QoL measures across groups | 1. Trajectory of quality of life | Latent class growth analysis | SAS 9.2 | 3 | 1. Quality of life steadily poor 2. Quality of life consistently moderate 3. Quality of life consistently good |
| 132 | Wang X et al (2017) | Identifying Patterns of Lifestyle Behaviors among People with Type 2 Diabetes in Tianjin, China: A Latent Class Analysis | Asia | China | Cross-sectional study | 1504 | Adult T2DM patients aged 18 years or older who were local residents for at least 5 years and were covered by special disease outpatient service insurance (SDOSI) | Expressed as Median (IQR) Overall: 63 (13)  1. Class I: 63 (12)  2. Class II: 63 (10)  3. Class III: 60 (13)  4. Class IV: 64 (13) | Overall: 741 (49.3%) 1. Class I: 436 (49.0%)  2. Class II: 33 (55.0%) 3. Class III: 109 (87.2%) 4. Class IV: 163 (38.0%) | Expressed as Mean(SD) Overall: 9 (9) 1. Class I: 9 (9) 2. Class II: 12 (11) 3. Class III: 8 (9) 4. Class IV: 9 (9) | primary. Questionnaire, clinical data from reviewing medical records | tertiary | Health Grouping/Profiling | 1. Regular exercise  2. Physical activity/week  3. Sedentariness  4. Staple food/day  5. Vegetables/day  6. Cooking oil/day  7. Salt intake/day  8. Current smoker  9. Current drinker | Latent class analysis | SAS 9.3 | 4 | 1. Healthy behavioral group  2. Unhealthy diet and less activity group  3. Smoking and drinking group  4. Sedentary and extremely inactive group |
| 133 | Wang Y et al (2003) | Phenotypic heterogeneity and associations of two aldose reductase gene polymorphisms with nephropathy and retinopathy in type 2 diabetes. | Asia | Hong Kong | Cross-sectional study | 738 | Adult T2DM patients in Hong Kong without microscopichematuria or known history of non– diabetes-related renal diseases and not on ACE inhibitors | Expressed as Mean (SD) 1. No nephropathy: 52.3 (12.6) 2. Nephropathy: 60.8 (11.1)  3. No retinopathy: 53.4 (13.0) 4. Retinopathy: 62.0 (9.6) | 1. No nephropathy: 178 (38.9%) 2. Nephropathy: 129 (46.1%) 3. No retinopathy: 229 (41.6%) 4. Retinopathy: 78 (41.7%) | Expressed in Mean(SD) 1. No nephropathy: 4.2 (4.7) 2. Nephropathy: 8.1 (6.3) 3. No retinopathy: 4.4 (4.6) 4. Retinopathy: 9.4 (6.9) | primary. Clinical examination and biochemical assays, Genotyping | tertiary | Health Grouping/Profiling - Assess differential risk of diabetic related complications across groups | 1. Presence of nephropathy  2. Presence of retinopathy | Judgemental splitting by patients' clinical characteristics | version 9.0; SPSS | 1. Nephropathy: 2 2. Retinopathy: 2 | Presence of nephropathy 1. No nephropathy 2. Nephropathy  Presence of retinopathy  1. No retinopathy 2. Retinopathy |
| **S/No** | **Authors (Year)** | **Article Name** | **Continent** | **Country** | **Study design** | **Sample size** | **T2DM Patient population** | **Mean / Median Age of patients** | **Gender [Male (%)]** | **Duration of diabetes** | **Data source** | **Study setting** | **Objective of segmentation** | **Segmentation variables** | **Statistical methods used** | **Software** | **Number of segments** | **Categories of segments** |
| 134 | Wang Y et al (2014) | Racial disparities in cardiovascular risk factor control in an underinsured population with Type 2 diabetes. | North America | United States of America | Cross-sectional study | 27704 | Adult African American and White patients who were newly diagnosed with type 2 diabetes | Expressed as Mean(SD) Men 1. African american: 50.8 (10.4)  2. White: 54.4 (10.5)   Women 1. African american: 51.6 (10.3)  2. White: 54.0 (10.7) | 1. White: 5447 (43.3%) 2. African American: 5633 (37.7%) | NA, newly diagnosed | Secondary. Administrative, anthropometric, laboratory, and clinical diagnosis  data collected at public hospitals and affiliated clinics in Louisiana | primary and tertiary | - Assess diabetic control across groups  - Assess treatment outcomes | 1. Patients' race | Judgemental Splitting by patients' sociodemographic and/or economic attributes | SAS for Windows, version 9.3 | 2 | 1. African American 2. White |
| 135 | Wolffenbuttel BH et al (2013) | Ethnic differences in glycemic markers in patients with type 2 diabetes. | Australia, Asia, Europe, North America and South America | Argentina, Australia, Brazil, Canada, Greece, Hungary, India, Romania, Spain, The Netherlands, and United States of America | Cross-sectional study | 1879 | Adult T2DM patients aged 30 to 80 years of age with HbA1c.>7.0% (53 mmol/mol) with use of at least two oral BG–lowering agents for at least 90 days before the study | Expressed in Mean(SD) 1. All: 57 (10) 2. Caucasian: 59 (10) 3. Asian: 53 (9) 4. Hispanic: 54 (10) 5. African descent: 55 (11) | 1. All: 985 (52.4%) 2. Caucasian 702 (56.6%) 3. Asian: 139 (45.7%) 4. Hispanic: 92 (40.9%) 5. African descent 51 (46.8%) | Expressed as Median (IQR) 1. All: 8.0 (5.0-12.5) 2. Caucasian 9.0 (6.0 - 13.0) 3. Asian 7.0 (4.0-12.0) 4. Hispanic 8.0 (5.0-13.0) 5. African descent 7.0 (4.0 - 12.0) | -secondary. From the DURABLE trial | not specified | - Health Grouping/Profiling | 1. Patients' ethnicity | Judgemental Splitting by patients' sociodemographic and/or economic attributes | PASW Statistics (Version 20) | 4 | 1. Caucasian 2. Asian 3. Hispanic 4. African descent |
| 136 | Won JC et al (2017) | Clinical Phenotype of Diabetic Peripheral Neuropathy and Relation to Symptom Patterns: Cluster and Factor Analysis in Patients with Type 2 Diabetes in Korea | Asia | Korea | Cross-sectional study | 1073 | T2DM patients with diabetic peripheral neuropathy (DPN) | Expressed as Mean(SD) 1. Cluster 1: 61.7 (9.9)  2. Cluster 2: 62.6 (11.5)  3. Cluster 3: 62.7 (10.5) | Entire Patients 1. Cluster 1: 246 (54.9%) 2. Cluster 2: 217 (38.6%)  3. Cluster 3: 130 (39.6%) | Not specified | -secondary -Data from previous cross-sectional observational study conducted on patients with DPN in 2010 which include medical records and self-reported questionnaires | tertiary | - Health Grouping/Profiling - Assess differential risk of diabetic related complications across groups | 1. Michigan Neuropathy Screening Instrument (MNSI) scores 2. Modified Korean version of Brief Pain Inventory-Short Form (BPI-SF) scores 3. Medical outcomes study (MOS) sleep scale scores 4. Korean version of the EuroQOL (EQ-5D) scores | Hierarchical cluster analysis | SPSS statistics for windows. version 18.0 & SAS 9.4 | 3 | 1. Cluster 1: Asymptomatic 2. Cluster 2: Moderate symptoms and disturbed sleep 3. Cluster 3: Severe symptoms and decreased quality of life |
| 137 | Xu D et al (2016) | Fasting plasma glucose variability and all-cause mortality among type 2 diabetes patients: a dynamic cohort study in Shanghai, China. | Asia | China | Retrospective cohort study | 8871 | Adult T2DM patients enrolled in a standardized management system of diabetes in Shanghai, China in 2007 with ≥ 3 records fasting plasma glucose (FPG) in the first one-year of following-up | Expressed as Mean (SD) 1 (≤ 4.25): 72.27 (10.90) 2 (4.25~7.75): 72.31 (10.96) 3 (7.75~13.45): 71.70 (10.84) 4 (> 13.45): 71.45 (11.36) | 1 (≤ 4.25): 1025 (46.28)  2 (4.25~7.75): 993 (44.73)  3 (7.75~13.45): 910 (41.03)  4 (> 13.45): 981 (44.23) | 1 (≤ 4.25): 12.83 (5.55)  2 (4.25~7.75): 12.70 (5.30)  3 (7.75~13.45): 12.85 (5.50)  4 (> 13.45): 13.70 (6.39) | secondary. Data from standardized management system of diabetes in Shanghai, China | primary | 1) Health Grouping/Profiling 2) Assess mortality | 1. Coefficient of variation of FPG levels | Judgemental splitting by patients' clinical characteristics | SAS version 9.1 | 4 | 1. Coefficient of variation of fasting plasma glucose levels (≤ 4.25) 2. Coefficient of variation of fasting plasma glucose levels (4.25 - 7.75) 3. Coefficient of variation of fasting plasma glucose levels (7.75 - 13.45)  4. Coefficient of variation of fasting plasma glucose levels (> 13.45) |
| **S/No** | **Authors (Year)** | **Article Name** | **Continent** | **Country** | **Study design** | **Sample size** | **T2DM Patient population** | **Mean / Median Age of patients** | **Gender [Male (%)]** | **Duration of diabetes** | **Data source** | **Study setting** | **Objective of segmentation** | **Segmentation variables** | **Statistical methods used** | **Software** | **Number of segments** | **Categories of segments** |
| 138 | Xu ZR et al (2001) | Clustering of cardiovascular risk factors with diabetes in Chinese patients: the effects of sex and hyperinsulinaemia. | Asia | China | Cross-sectional study | 654 | Adult chinese patients with T2DM presenting for the first time to the hospital in Beijing | Expressed as Median(IQR 25-75%) 1. G0: 54 (48-64) 2. G1: 57 (46-65) 3. G2: 58 (50-65) 4. G3: 58 (45-62) 5. G4/5: 60 (51-65) | 1. G0: 87 (64%) 2. G1: 117 (63%) 3. G2: 82 (52%) 4. G3: 57 (49%) 5. G4/5: 19 (33%) | Expressed as Median (IQR 25-75%) 1. G0: 1.5 (0.08-6) 2. G1: 3 (0.13-7) 3. G2: 2 (0.08-8) 4. G3: 1.2 (0.08-6) 5. G4/5: 1 (0.08-5.1) | primary Clinical laboratory results, detailed history, and physical examination | tertiary | 1) Health Grouping/Profiling 2) Assess differential risk of diabetic related complications across groups 3) Assess non-diabetes metabolic derangements (e.g. lipid, blood pressure) across groups | Number of cardiovascular risk factors 1. Hypertension 2. Hypertriglyceridemia 3. Abnormal HDL level 4. Obesity 5. Central obesity | Cluster analysis | NCSS97 (number cruncher statistical system) | 5 | 1. G0 (no risk factors) 2. G1 (1 risk factor) 3. G2 (2 risk factors) 4. G3 (3 risk factors) 5. G4/5 (4 or 5 risk factors) |
| 139 | Yang W et al (2016) | Clinical Characteristics of Young Type 2 Diabetes Patients with Atherosclerosis. | Asia | China | Cross-sectional study | 2199 | T2DM patients aged 20 to 85 who were hospitalized for treatment in the ward of the Department of endocrinology and metabolism of Peking university of People's Hospital from 2009-2014 | Expressed as Median (25th percentile, 75th percentile) All: 56.0(20,85)  1. Young atherosclerosis group: 42.0(27,45) 2. Young non-atherosclerosis group: 36.0(20,45) 3. Older atherosclerosis group: 60.0(46,85) 4. Older non-atherosclerosis group: 53.0(46,84) | All: 59.8(1316)  1. Young atherosclerosis group: 82.3(153)  2. Young non-atherosclerosis group: 75.1(154)  3. Older atherosclerosis group: 56.4(912) 4. Older non-atherosclerosis group: 50.5(97) | Expressed as Median (25th percentile, 75th percentile) All: 9.0(0.1, 47.0) 1. Young atherosclerosis group: 4.0(0.1,19.0)  2. Young non-atherosclerosis group: 2.0(0.1,15.0) 3. Older atherosclerosis group: 10.0(0.1,47.0) 4. Older non-atherosclerosis group: 6.0(0.1,30.8) | -Secondary -Inpatient database at Peking University People's Hosital | Tertiary | - Health Grouping/Profiling | 1. Presence of atherosclerosis 2. Patient age group (Young: <= 45 years old, Older: > 45 years old) | Judgemental splitting by patients' clinical characteristics, sociodemographic and/or economic attributes | SPSS version 19.0 | 4 | 1. Young atherosclerosis group 2. Young non-atherosclerosis group 3. Older atherosclerosis group 4. Older non-atherosclerosis group |
| 140 | Yeung RO et al (2014) | Metabolic profiles and treatment gaps in young-onset type 2 diabetes in Asia (the JADE programme): a cross-sectional study of a prospective cohort. | Asia | Hong Kong, India, Philippines, China, South Korea, Vietnam, Singapore, Thailand, Taiwan | Cross-sectional study | 41 029 | Adult T2DM patients aged 18 years or older from participating institutions | Expressed in Mean(SD) 1. Young-onset diabetes: 44.7 (10.5)  2. Late-onset diabetes:60.3 (0.7) | 1. Young-onset diabetes: 4255 (57%) 2. Late-onset diabetes: 17446 (52%) | Expressed in Median (IQR) 1. Young-onset diabetes: 10.0 (3.0-18.0) 2. Late-onset diabetes: 5.0 (2.0-11.0) | Primary, clinical history and existing health records from attending physicians | primary and tertiary | Health Grouping/Profiling - Assess differential risk of diabetic related complications across groups - Assess non-diabetes metabolic derangements (e.g. lipid, blood pressure) across groups | 1. Age of diabetes onset | Judgemental splitting by patients' clinical characteristics | SPSS (version 20.0) | 2 | 1. Young-onset diabetes (age of diagnosis of younger than 40) 2. Late-onset diabetes (40 years and above) |
| 141 | Yeung RO et al (2018) | Determinants of hospitalization in Chinese patients with type 2 diabetes receiving a peer support intervention and JADE integrated care: The PEARL randomised controlled trial | Asia | China | Randomized controlled trial | 372 | Chinese patients with T2DM receiving multidisciplinary care in the Joint Asia Diabetes Evaluation (JADE) programme | Expressed as Mean(SD) 1. Hospital admission: 54.1 (9.4) 2. No hospital admission: 56.7 (8.6) | 1. Hospital admission: 274 (56.6%) 2. No hospital admission: 81 (56.3%) | Expressed as Mean(SD) 1. Hospital admission: 9.1 (7.7) 2. No hospital admission: 10.3 (7.8) | -secondary -electronic medical record (Clinical Managemant Sytem, CMS) | tertiary | - Health Grouping/Profiling - Assess differential healthcare utilization | 1. Hospitalization admission | Judgemental splitting by patients' clinical characteristics | SPSS (version 20.0, Chicago, USA) and SAS (version 9.3) | 2 | 1. Hospital admission 2. No hospital admission |
| **S/No** | **Authors (Year)** | **Article Name** | **Continent** | **Country** | **Study design** | **Sample size** | **T2DM Patient population** | **Mean / Median Age of patients** | **Gender [Male (%)]** | **Duration of diabetes** | **Data source** | **Study setting** | **Objective of segmentation** | **Segmentation variables** | **Statistical methods used** | **Software** | **Number of segments** | **Categories of segments** |
| 142 | Yoda N et al (2008) | Classification of adult patients with type 2 diabetes using the Temperament and Character Inventory. | Asia | Japan | Cross-sectional study | 89 | Adult patients with T2DM under treatment with outpatient clinics of the Department of Endocrinology and Metabolism University Hospital | Expressed as mean (SD) All: 63.34 (10.21) 1. Cluster 1: 65 (9.71) 2. Cluster 2: 61.78 (10.53) | All: 54 (60.7%) 1. Cluster 1: 27 (62.8%) 2. Cluster 2: 27 (58.7%) | Expressed in mean (SD) All: 14.10 (10.60) 1. Cluster 1: 15.2 (10.85) 2. Cluster 2: 13.1 (10.36) | -primary -psychological test questionnaire -Patient's HbA1c levels | tertiary | - Health Grouping/Profiling | 1. Psychological characteristics based on Temperament and Character Inventory (TCI) assessment | Cluster analysis | SPSS version 12.0 | 2 | 1. Cluster 1: Low novelty seeking, high harm avoidance, low reward dependence 2. Cluster 2: High novelty seeking, low harm avoidance, high reward dependence |
| 143 | Zhang XL et al (2018) | The effects of cardiovascular risk factor combined anti-platelet therapy and the risk of cerebrovascular events in patients with T2DM in an urban community over 96-months follow-up: The Beijing communities diabetes study 19. | Asia | China | Prospective cohort study | 3413 | Adult T2DM patients aged between 20 and 80 years with ability to provide consent | Expressed as Mean(SD) 1: 66 (9) 2: 65 (10) 3: 63 (10) 4: 59 (11) total: 63 (10) | 1: 334 (39%) 2: 332 (37%) 3: 338 (41%) 4: 342 (41% total: 1365 (40%) | Expressed as Mean(SD) 1: 8.5 (6.6)  2: 8.9 (7.0)  3: 8.2 (6.6)  4: 7.3 (6.3) total: 8.2 (6.7) | primary Laboratory data was obtained | not specified | - Assess differential risk of diabetic related complications across groups - Assess non-diabetes metabolic derangements (e.g. lipid, blood pressure) across groups - Assess treatment outcomes | 1. Total metabolic score (TMS) | Cluster analysis | SAS software  Version 9.1 | 4 | 1. Group 1 (24 < Total metabolic score < 40) 2. Group 2 (40 < Total metabolic score < 47) 3. Group 3 (47 < Total metabolic score < 55) 4. Group 4 (55 < TMS < 87) |
| 144 | Zhao W et al (2014) | Sex differences in the risk of stroke and HbA(1c) among diabetic patients. | North America | United States of America | Prospective cohort study | 30,154‬ | Adult T2DM patients who had newly diagnosed diabetes without a history of stroke or coronary heart disease (CHD) at baseline | Expressed in Mean(SD) 1. Male: 50.90 (10.1)  2. Female: 51.48 (10.1) | 10876 (36.1%) | NA, newly diagnosed | secondary, electronic medical records | tertiary | - Assess differential risk of diabetic complications across groups - Assess diabetic control across groups | 1. Patient's gender | Judgemental Splitting by patients' sociodemographic and/or economic attributes | PASW for Windows, version 20.0 | 2 | 1. Male 2. Female |
| 145 | Zheng W et al (2011) | Factor analysis of diabetic nephropathy in Chinese patients | Asia | China | Cross-sectional study | 873 | Patients with T2DM admitted to Endocrinology Department of Peking University People’s Hospital from February 2006 to July 2009 | Expressed as M(quantile)  Total: 56.0 (48.0–66.0)   Expressed as Mean(SD) 1. Normo-Alb: 55.96 (11.92) 2, Micro-Alb: 59.74 (11.85) 3. Macro-Alb: 64.42 (9.62) | Total: 527 (60.4%)  1. Normo-Alb: 412 (61.7%) 2. Micro-Alb: 82 (55.4%) 3. Macro-Alb: 33 (57.9%) | Expressed as M(Quantile)  Total: 7.0 (1.0–12.0) 1. Normo-Alb: 6.0 (1.0–11.0)  2. Micro-Alb: 8.5 (3.0–14.8) 3. Macro-Alb: 13.4 +/- 7.2 | Primary  Clinical data gathered through history inquiry, urine ketone dip test, blood sampling and biochemical assay | tertiary | 1) Health Grouping/Profiling 2) Assess differential risk of diabetic related complications across groups | 1. Albuminuria status | Cluster analysis | SPSS software package | 3 | 1. Normoalbuminuria 2. Microalbuminuria 3. Macroalbuminuria |
| **S/No** | **Authors (Year)** | **Article Name** | **Continent** | **Country** | **Study design** | **Sample size** | **T2DM Patient population** | **Mean / Median Age of patients** | **Gender [Male (%)]** | **Duration of diabetes** | **Data source** | **Study setting** | **Objective of segmentation** | **Segmentation variables** | **Statistical methods used** | **Software** | **Number of segments** | **Categories of segments** |
| 146 | Zhou X et al (2016) | Prevalence of Obesity and Its Influence on Achievement of Cardiometabolic Therapeutic Goals in Chinese Type 2 Diabetes Patients: An Analysis of the Nationwide, Cross-Sectional 3B Study. | Asia | China | Cross-sectional study | 24,512 | Adult T2DM patients aged 18 years of age or older and were diagnosed with type 2 diabetes at least 6 months prior to study screening | Expressed in Mean(SD) Total: 62.5 (11.8)  BMI:  1. < 24: 63.5 (11.6)  2. 24-27.9: 62.2 (11.7)  3. >= 28.0: 60.8 (12.4)  WC: 1. Normal WC: 62.0 (11.8)  2. Central obesity: 60.6 (12.1) | Total:11543 (47.1%) BMI:  1. < 24: 4524 (45.9%)  2. 24-27.9: 5291 (50.2%)  3. >= 28.0: 1728 (42.2%)  WC: 1. Normal WC: 1345 (46.3%) 2. Central obesity: 4614 (69.5%) | Expressed in Median (25th, 75th percentile) Total: 6.2 (2.7, 11.3) BMI:  1. < 24: 6.7 (2.7, 12.0) 2. 24-27.9: 6.0 (2.7,11.0)  3. >= 28.0: 5.9 (2.5, 10.9) WC: 1. Normal WC: 6.0 (2.6, 11.0) 2. Central obesity: 5.7 (2.4, 10.8) | Secondary. Patient demographic data, anthropometric measurements, medications, and blood glucose and lipid profiles of 24,512 type 2 diabetes patients from a large, geographically diverse study (CCMR-3B) | tertiary | Assess non-diabetes metabolic derangements (e.g. lipid, blood pressure) across groups - Assess diabetic control across groups | 1. Body mass index (BMI)  2. Waist circumference (WC) | Judgemental splitting by patients' clinical characteristics | SAS version 9.2 | 1. Body mass index (BMI): 3  2. BMI and Waist circumference (WC): 2 | Body mass index (BMI)  1. <24  2. 24 - 27.9 3. >=28  BMI and Waist circumference 1. BMI>=24 and normal waist circumference 2. BMI>=24 and central obesity |
| 147 | Zinman B et al (2004) | Phenotypic characteristics of GAD antibody-positive recently diagnosed patients with type 2 diabetes in North America and Europe. | Europe and North America | U.S. and Canada, Austria, Belgium, Czech Republic, Denmark, Finland, France, Germany, Hungary, Italy, Ireland, the Netherlands, Norway, Spain, Sweden, and United Kingdom | Randomized double-blind parallel-group trial | 4,134 | Adult T2DM patients aged 30–75 years with insufﬁciently controlled fasting plasma glucose concentration (between 7 and 10 mmol/l) with routine diet and exercise intervention during the screening period | Expressed in Mean(SE) 1. GAD positive: 57.0 +/- 0.799 2. GAD negative: 56.5 +/- 0.159 | 1. GAD positive: 97 (55.8%) 2. GAD negative: 2285 (57.7%) | not specified | Primary, using A Diabetes Outcome Progression Trial (ADOPT) data including standard anthropometric measurements and blood pressure, routine lipid measurements and HbA1c | not specified | Health Grouping/Profiling | 1. Glutamic acid decarboxylase (GAD) antibody status | Judgemental splitting by patients' clinical characteristics | not specified | 2 | 1. GAD antibody positive 2. GAD antibody negative |
| **S/No** | **Authors (Year)** | **Article Name** | **Continent** | **Country** | **Study design** | **Sample size** | **T2DM Patient population** | **Mean / Median Age of patients** | **Gender [Male (%)]** | **Duration of diabetes** | **Data source** | **Study setting** | **Objective of segmentation** | **Segmentation variables** | **Statistical methods used** | **Software** | **Number of segments** | **Categories of segments** |
| 148 | Zou X et al (2017) | The characteristics of newly diagnosed adult early-onset diabetes: a population-based cross-sectional study. | Asia | China | Cross-sectional study | 2801 | Adult, Chinese T2DM patients aged 20–75 years without known diabetes or pre-diabetes in a national cross-sectional survey | Expressed as Mean (95% CI) 1. 20-43: 36 (35, 36) 2. 44-52: 48 (47, 48) 3. 53- 61: 57 (57, 57) 4. >61: 68 (68, 69) | 20-43: 338 (51.7%) 44-52: 346 (48.5%) 53- 61: 290 (40.1%) >61: 308 (43.3%) | NA. Newly diagnosed | Primary. Complete oral glucose tolerance test (OGTT) data and demographic information were collected via trained and qualified physicians using questionnaire surveys and physical examinations | tertiary | - Health Grouping/Profiling | 1. Age of diabetes onset | Judgemental splitting by patients' clinical characteristics | SPSS for Windows 19.0 software | 4 | 1. Diabetes onset at 20–43 years 2. Diabetes onset at 44–52 years 3. Diabetes onset at 53–61 years 4. Diabetes onset at more than 61 years |
