# Supplementary material for: Population segmentation of type 2 diabetes mellitus patients and its clinical applications - a scoping review
Source: BMC Med Res Methodol. 2021 Mar 11;21:49. doi: 10.1186/s12874-021-01209-w (PMC7953703; doi:10.1186/s12874-021-01209-w)
Supplement: Supplementary file 3 — Additional file 3 Funding sources for included studies (n = 148) [file 12874_2021_1209_MOESM3_ESM.docx]

**Supplementary File 3.** **Funding sources for included studies (n=148)**

| **S/No** | **Authors (Year)** | **Article name** | **Funding source** | **Funding source category** |
| --- | --- | --- | --- | --- |
| 1 | Aguilar-Salinas CA et al (2001) | Early-onset type 2 diabetes: metabolic and genetic characterization in the mexican population. | Grant IN207493 from the Direccio ́n General de Asuntos del Personal Acade ́mico, UNAM. | Government |
| 2 | Al-Mukhtar SB et al (2012) | General and gender characteristics of type 2 diabetes mellitus among the younger and older age groups | No funding |  |
| 3 | Amato MC et al (2016) | Phenotyping of type 2 diabetes mellitus at onset on the basis of fasting incretin tone: Results of a two-step cluster analysis | University of Palermo ‘Fondo Ricerca di Ateneo 2012’ | Professional organizations |
| 4 | Amutha A et al (2012) | Clinical profile and complications of childhood- and adolescent-onset type 2 diabetes seen at a diabetes center in south India. | No funding |  |
| 5 | Barrot-de la Puente J et al (2015) | Older type 2 diabetic patients are more likely to achieve glycaemic and cardiovascular risk factors targets than younger patients: analysis of a primary care database | 1. Catalan Diabetes Association, the Catalan Health Department  2. Novartis Farmaceutica S.A. | 1. Professional organizations 2. Private industry |
| 6 | Basanta-Alario ML et al (2016) | Differences in clinical and biological characteristics and prevalence of chronic complications related to aging in patients with type 2 diabetes. | 1. Department of Health of the Valencian Government 2. CIBER of diabetes and associated metabolic diseases (CIBERDEM) (an initiative of Instituto de Salud Carlos III.) | 1. Government 2. Professional organization |
| 7 | Benhalima K et al (2011) | Type 2 diabetes in younger adults: clinical characteristics, diabetes-related complications and management of risk factors. | No funding |  |
| 8 | Berkowitz SA et al (2013) | Age at type 2 diabetes onset and glycaemic control results from the National Health and Nutrition Examination Survey (NHANES) 2005-2010. | 1. Institutional National Research Service Award T32HP10251 2. Ryoichi Sasakawa Fellowship Fund 3. General Medicine Division at Massachusetts General Hospital. | 1. Government 2. Professional organization 3. Government |
| 9 | Berry E et al (2017) | Illness perception clusters and relationship quality are associated with diabetes distress in adults with Type 2 diabetes | No funding |  |
| 10 | Bidel S et al (2006) | Coffee consumption and risk of total and cardiovascular mortality among patients with type 2 diabetes. | 1. Juho Vainio Foundation 2. Finnish Foundation for Cardiovascular Research 3. Academy of Finland | 1. Foundation 2. Foundation  3. Professional organizations |
| 11 | Blak BT et al (2016) | Weight change and healthcare resource use in English patients with type 2 diabetes mellitus initiating a new diabetes medication class. | AstraZeneca, Global Payer Evidence and Pricing, Global Medicines Development | Private industry |
| 12 | Bo S et al (2013) | Mortality outcomes of different sulphonylurea drugs: the results of a 14-year cohort study of type 2 diabetic patients. | Grant from Regione Piemonte 2009 | Government |
| 13 | Bruce DG et al (2000) | Glycemic control in older subjects with type Glycemic control in older subjects with type 2 diabetes mellitus in the Fremantle Diabetes Study. | Raine Foundation, Perth, Western Australia. | Foundation |
| 14 | Bruce DG et al (2016) | Comorbid Anxiety and Depression and Their Impact on Cardiovascular Disease in Type 2 Diabetes: The Fremantle Diabetes Study Phase II. | 1. National Health and Medical Research Council of Australia (Project grants 513781 and 1042231). 2. National Health and Medical Research Council of Australia Practitioner Fellowship. | 1. Government 2. Government |
| 15 | Bruno G et al (1999) | Cardiovascular risk profile of type 2 diabetic patients cared for by general practitioners or at a diabetes clinic: a population-based study. | No funding |  |
| 16 | Chan JC et al (2014) | Premature mortality and comorbidities in young-onset diabetes: a 7-year prospective analysis. | 1. Hong Kong Foundation for Research and Development  2. Liao Wun Yuk Memorial Diabetes Research Fund of the Chinese University of Hong Kong. | 1. Foundation 2. Professional organizations |
| 17 | Chan KS et al (2012) | Do diabetic patients living in racially segregated neighborhoods experience different access and quality of care? | Grant# 1P60MD00214-07 from the National Institute of Minority Health and Health Disparities (NIMHD) of the National Institutes of Health (NIH). | Government |
| 18 | Chao CT et al (2018) | Both pre-frailty and frailty increase healthcare utilization and adverse health outcomes in patients with type 2 diabetes mellitus. | 1. National Taiwan University Hospital BeiHu branch 2. Ministry of Science and Technology, Taiwan | 1. Professional organizations 2. Government |
| 19 | Chen HL et al (2015) | Changes in prevalence of diabetic complications and associated healthcare costs during a 10-year follow-up period among a nationwide diabetic cohort. | National Science Council | Government |
| 20 | Cheng XB et al (2012) | Obesity and low target attainment rates in Chinese with type 2 diabetes. | No funding |  |
| **S/No** | **Authors (Year)** | **Article name** | **Funding source** | **Funding source category** |
| 21 | Cheng Y et al (2014) | Cardiometabolic risk profiles associated with chronic complications in overweight and obese type 2 diabetes patients in South China. | 1. Diabetes Society of Guangdong Provincial Medical Association  2. Special grant for overweight and obese type 2 diabetic patients in Guangdong province (No. 2011-DM-01). | 1. Professional organization 2. Government |
| 22 | Cheong AT et al (2013) | Poor glycemic control in younger women attending Malaysian public primary care clinics: findings from adults diabetes control and management registry. | National Institutes of Health, Ministry of Health, Malaysia research grant | Government |
| 23 | Chew BH et al (2011) | Ethnic differences in glycaemic control and complications: the adult diabetes control and management (ADCM), Malaysia. | No funding |  |
| 24 | Chew BH et al (2013) | Type 2 diabetes mellitus patient profiles, diseases control and complications at four public health facilities- A cross-sectional study based on the Adult Diabetes Control and Management (ADCM) registry 2009 | Ministry of Health,  Malaysia. | Government |
| 25 | Coleman SM et al (2013) | Depression and death in diabetes; 10-year follow-up of all-cause and cause-specific mortality in a diabetic cohort. | National Institute of Mental Health | Government |
| 26 | De Cosmo S et al (2014) | Kidney dysfunction and related cardiovascular risk factors among patients with type 2 diabetes. | No funding |  |
| 27 | de Rekeneire N et al (2003) | Racial differences in glycemic control in a well-functioning older diabetic population: findings from the Health, Aging and Body Composition Study. | Contracts NO1-AG-6-2101, NO1-AG-6-2103, and NO1-AG-6-2106 of National  Institute on Aging. | Government |
| 28 | de Vries McClintock HF et al (2016) | Patterns of Adherence to Oral Hypoglycemic Agents and Glucose Control among Primary Care Patients with Type 2 Diabetes. | No funding |  |
| 29 | Demmer RT et al (2015) | Sex Differences in the Association Between Depression, Anxiety, and Type 2 Diabetes Mellitus | National Institutes of Health Grants | Government |
| 30 | Duan JG et al (2015) | Sex differences in epidemiology and risk factors of acute coronary syndrome in Chinese patients with type 2 diabetes: a long-term prospective cohort study. | 1. Direct Grant for Research (reference no. 2005.1.082)  2. S. H. Ho Cardiovascular and Stroke Center of the Chinese University of Hong Kong. 3. Croucher Senior Medical Research Fellowship | 1. Professional organizations 2. Professional organizations 3. Professional organizations |
| 31 | Egede LE et al (2015) | Differential impact of mental health multimorbidity on healthcare costs in diabetes. | Grant #IIR-06-219 funded by the VHA’s HSR&D program. | Government |
| 32 | Elder DH et al (2016) | Mean HbA1c and mortality in diabetic individuals with heart failure: a population cohort study. | 1. Chest Heart & Stroke Scotland Fellowship grant. 2. European Foundation for the Study of Diabetes (EFSD) Research Fellowship grant. | 1. Government 2. Foundation |
| 33 | Elissen AMJ et al (2017) | Differences in biopsychosocial profiles of diabetes patients by level of glycaemic control and health-related quality of life: The Maastricht Study. | Novo Nordisk Farma B.V. | Private industry |
| 34 | El-Kebbi IM et al (2003) | Association of Younger Age With Poor Glycemic Control and Obesity in Urban African Americans With Type 2 Diabetes | 1. Agency for Healthcare and Research Quality 2. Emory Medical Care Foundation | 1. Government 2. Foundation |
| 35 | Emanuele N et al (2005) | Ethnicity, race, and baseline retinopathy correlates in the veterans affairs diabetes trial. | No funding |  |
| 36 | Ernande L et al (2017) | Clinical Implications of Echocardiographic Phenotypes of Patients With Diabetes Mellitus | 1. Grant from the Société Francophone du Diabète (formerly the Association of French Language for the Study of Diabetes Mellitus and Metabolic Diseases, grant number D20515)  2. Grant from the Programme Hospitalier de Recherche Clinique (PHRC 2009-A00089-48) 3. French National Agency through the Recherche Hospital-Universitaire-Cardiac & Skeletal Muscle Alteration in Relation to Metabolic Diseases and Ageing: Role of Adipose Tissue (RHU-CARMMA) Grant ANR-15-RHUS-0003. | 1. Professionl Organizations 2. Government 3. Government |
| 37 | Escalada J et al (2014) | Outcomes and healthcare resource utilization associated with medically attended hypoglycemia in older patients with type 2 diabetes initiating basal insulin in a US managed care setting. | Sanofi | Private industry |
| 38 | Escobar C et al (2011) | Prevalence and clinical profile and management of peripheral arterial disease in elderly patients with diabetes. | Sanofi | Private industry |
| 39 | Ezenwaka CE et al (2002) | Differences in Cardiovascular Disease Risk Factors in Elderly and Younger Patients with Type 2 Diabetes in the West Indies | University of the West Indies, St Augustine,  Trinidad. | Professional organization |
| 40 | Fink JT et al (2018) | Blood Pressure Control and Other Quality of Care Metrics for Patients with Obesity and Diabetes: A Population-Based Cohort Study. | Grant R21 HS021899 from the Agency for Healthcare Research and Quality. | Professional organizations |
| **S/No** | **Authors (Year)** | **Article name** | **Funding source** | **Funding source category** |
| 41 | Franch-Nadal J et al (2014) | Metabolic control and cardiovascular risk factors in type 2 diabetes mellitus patients according to diabetes duration. | 1. Merck Sharp & Dohme Spain 2. Fundació d’Atenció Primària. | 1. Private industry 2. Foundation |
| 42 | Gao F et al (2017) | Latent class analysis suggests four classes of persons with type 2 diabetes mellitus based on complications and comorbidities in Tianjin, China: a cross-sectional analysis. | 1. Project of Tianjin Municipal Human Resources and Social Security Bureau (TMIR 201502) 2. Grants of National Natural Science Foundation of China (71373175)  3. Ministry of Education Humanities and Social Science Planning | 1. Government 2. Foundation 3. Government |
| 43 | Gao Y et al (2016) | The prevalence of mild cognitive impairment with type 2 diabetes mellitus among elderly people in China: A cross-sectional study | National Natural Science Foundation of China (grant number: 81130053). | Foundation |
| 44 | Gariepy G et al (2011) | Types of smokers in a community sample of individuals with Type 2 diabetes: a latent class analysis. | Canadian  Institute of Health Research | Government |
| 45 | Genovese S et al (2006) | Clinical phenotype and beta-cell autoimmunity in Italian patients with adult-onset diabetes. | No funding |  |
| 46 | Ghane BM et al (2015) | Association of Major Dietary Patterns with General and Abdominal Obesity in Iranian Patients with Type 2 Diabetes Mellitus. | No funding |  |
| 47 | Göbl CS et al (2010) | Sex-specific differences in glycemic control and cardiovascular risk factors in older patients with insulin-treated type 2 diabetes mellitus. | No funding |  |
| 48 | Grégoire JP et al (2010) | Persistence patterns with oral antidiabetes drug treatment in newly treated patients--a population-based study. | Merck Frosst Canada Ltd | Private industry |
| 49 | Grenier J et al (2018) | Blood Pressure Management in Adults With Type 2 Diabetes: Insights From the Diabetes Mellitus Status in Canada (DM-SCAN) Survey. | Merck Canada Inc | Private industry |
| 50 | Griffiths RI et al (2012) | Epidemiology and outcomes of previously undiagnosed diabetes in older women with breast cancer: an observational cohort study based on SEER-Medicare. | No funding |  |
| 51 | Gucciardi E et al (2011) | Profiles of smokers and non-smokers with type 2 diabetes: initial visit at a diabetes education centers. | Canadian Diabetes Associ-  ation | Professional organizations |
| 52 | Gunathilake W et al (2010) | Cardiovascular and metabolic risk profiles in young and old patients with type 2 diabetes | No funding |  |
| 53 | Gunzler D et al (2017) | Psychosocial Features of Clinically Relevant Patient Subgroups With Serious Mental Illness and Comorbid Diabetes. | No funding |  |
| 54 | Hanai K et al (2012) | Gender differences in the association between HDL cholesterol and the progression of diabetic kidney disease in type 2 diabetic patients. | No funding |  |
| 55 | Handisurya A et al (2011) | Clinical characteristics, modalities and complications of diabetic patients with migration background at a Central European University Clinic. | No funding |  |
| 56 | Hari KKV et al (2014) | Clinical profile of patients using normal, high and very high insulin doses in type 2 diabetes. | No funding |  |
| 57 | Harris EL et al (1999) | Black-white differences in risk of developing retinopathy among individuals with type 2 diabetes. | 1. American Diabetes Association's Lions SightFirst Retinopathy Research Program  2. National Institutes of Health-National Center for Research Resources Outpatient General Clinical Research Center Grant RR00722 | 1. Professional organization 2. Government |
| 58 | Harris MI et al (1999) | Racial and Ethnic Differences in Glycemic Control of Adults With Type 2 Diabetes | No funding |  |
| 59 | Harris MI et al (2001) | Racial and Ethnic Differences in Health Care Access and Health Outcomes for Adults With Type 2 Diabetes | No funding |  |
| 60 | Herman WH et al (2009) | Racial and ethnic differences in mean plasma glucose, hemoglobin A1c, and 1,5-anhydroglucitol in over 2000 patients with type 2 diabetes. | Lilly USA, LLC | Private industry |
| 61 | Hermans MP et al (2002) | Clinical, biophysical and biochemical variables from African-heritage subjects with type 2 diabetes. | 1. European Association for the Study of Diabetes (EASD) 2. Association Belge du Diabète (ABD). | 1. Professional organization 2. Professional organization |
| 62 | Hong CY et al (2004) | Ethnic differences among Chinese, Malay and Indian patients with type 2 diabetes mellitus in Singapore | Singapore National Medical Research Council grant I.C.  RP 3950340. | Government |
| 63 | Hsu CC et al (2014) | Associations between dietary patterns and kidney function indicators in type 2 diabetes | National Health Research Institute and National Science Council Grant  423 (NSC 98-2314-B-037 -044 -MY3) | Government |
| 64 | Jeong JH et al (2016) | Depression and mortality in people with type 2 diabetes mellitus, 2003 to 2013: A nationwide population-based cohort study | No funding |  |
| **S/No** | **Authors (Year)** | **Article name** | **Funding source** | **Funding source category** |
| 65 | Ji L et al (2014) | Hyperglycemia and duration of diabetes as risk factors for abnormal lipids: a cross sectional survey of 19,757 patients with type 2 diabetes in China. | Novo Nordisk, China | Private industry |
| 66 | Jiang R et al (2018) | Clinical Trajectories, Healthcare Resource Use, and Costs of Diabetic Nephropathy Among Patients with Type 2 Diabetes: A Latent Class Analysis | Takeda Development Center Americas, Inc | Private industry |
| 67 | Johnson JF et al (2017) | Real-world Clinical Outcomes Among Patients With Type 2 Diabetes Receiving Canagliflozin at a Specialty Diabetes Clinic: Subgroup Analysis by Baseline HbA(1c) and Age. | Janssen Scientific Affairs, LLC | Private industry |
| 68 | Kalsekar ID et al (2006) | Impact of depression on utilization patterns of oral hypoglycemic agents in patients newly diagnosed with type 2 diabetes mellitus: a retrospective cohort analysis. | No funding |  |
| 69 | Kaplan SH et al (2013) | Reducing racial/ethnic disparities in diabetes: the Coached Care (R2D2C2) project. | 1. The Robert Wood Johnson Foundation (Grants # 1051084 and #59758) 2. The NovoNordisk Foundation, Corporate Diabetes Programmes, Novo Nordisk, Bagsvaerd, Denmark 2. National Institute of Diabetes, Digestive and Kidney Diseases (R18DK69846 and K01DK078939) | 1. Foundation 2. Foundation 3. Government |
| 70 | Karpati T et al (2018) | Patient clusters based on HbA1c trajectories: A step toward individualized medicine in type 2 diabetes. | 1. Clalit Research Institute 2. Eli Lilly and Company. | 1. Professional 2. Private industry |
| 71 | Kaukua J et al (2001) | Clustering of cardiovascular risk factors in type 2 diabetes mellitus: prognostic significance and tracking. | No funding |  |
| 72 | Kautzky-Willer A et al (2010) | Sex-specific differences in metabolic control, cardiovascular risk, and interventions in patients with type 2 diabetes mellitus. | Austrian National Bank (ÖNB Jubiläumsfonds”: Nr: 13244) | Private industry |
| 73 | Ki M et al (2014) | Age-related differences in diabetes care outcomes in Korea: a retrospective cohort study. | Korean Health Insurance Service | Private Industry |
| 74 | Klisic A et al (2019) | Association between unfavorable lipid profile and glycemic control in patients with type 2 diabetes mellitus | Ministry of Education, Science and Technological Development, Republic of Serbia (Project number 175035). | Government |
| 75 | Kuznetsov VA et al (2010) | Clinical manifestations and risk factors of coronary artery disease in patients with diabetes mellitus in western Siberia. | No funding |  |
| 76 | Lee CL et al (2018) | Trajectories of fasting plasma glucose variability and mortality in type 2 diabetes. | No funding |  |
| 77 | Lee PG et al (2018) | Patterns of physical activity in sedentary older individuals with type 2 diabetes | 1. VA Rehabilitation Research and Development Career Development Award (5-IK2-RX001190) 2. VA Rehabilitation Research and Development Merit Award (E0–9606) 3. Office of Research and Development of the Department of Veterans Affairs 4. American Diabetes Association Award (1–06-JF-20). 4. National Institute of Health Claude Pepper Center (AG024824), Michigan Institute for Clinical and Health Research (UL1TR000433) 5. Michigan Diabetes Research and Training Center (DK020572) and K24 (AG109675) 6. Dorothy and Herman Miller Fund for Mobility Research in Older Adults. | 1. Government 2. Government 3. Governement 4. Professional organization 5. Government |
| 78 | Li C et al (2007) | Clustering of multiple healthy lifestyle habits and health-related quality of life among U.S. adults with diabetes. | No funding |  |
| 79 | Li H et al (2019) | The Effect of Symptom Clusters on Quality of Life Among Patients With Type 2 Diabetes | National Institutes of Health, National Institute of Diabetes and Digestive and Kidney Diseases (R01-DK59048). | Government |
| 80 | Li L et al (2015) | Identification of type 2 diabetes subgroups through topological analysis of patient similarity. | 1. NIH National Institute of Diabetes and Digestive and Kidney Diseases (NIDDK) (R01DK098242)  2. National Cancer Institute (NCI) (U54CA189201). | 1. Government 2. Government |
| 81 | Lim JH et al (2011) | Association between dietary patterns and blood lipid profiles in Korean adults with type 2 diabetes. | Catholic University of Korea, Research Fund, 2010 | Professional Organizations |
| 82 | Lipscombe C et al (2015) | Exploring trajectories of diabetes distress in adults with type 2 diabetes; a latent class growth modeling approach. | No funding |  |
| 83 | Liu H et al (2017) | Body mass index and mortality in patients with type 2 diabetes mellitus: A prospective cohort study of 11,449 participants. | No funding |  |
| 84 | Loh PT et al (2015) | Ethnic disparity in prevalence of diabetic kidney disease in an Asian primary healthcare cluster. | No funding |  |
| **S/No** | **Authors (Year)** | **Article name** | **Funding source** | **Funding source category** |
| 85 | Lu B et al (2007) | High prevalence of chronic kidney disease in population-based patients diagnosed with type 2 diabetes in downtown Shanghai | 1. Shanghai Science and Technology Commission (04dz19504) 2. Science Foundation of China (30230380) 3. National Natural Science Foundation of China (39900072) 4. Chinese High Tech Program (2002BA711A05 and 2001AA221201) 5. National Key Basic Research and Development Program (2002CB713703). | 1. Government 2. Foundation 3. Foundation 4. Government 5. Government |
| 86 | Luo M et al (2017) | Longitudinal trends in HbA1c and associations with comorbidity and all-cause mortality in Asian patients with type 2 diabetes: A cohort study. | Biomedical Research Council of A*STAR. | Private industry |
| 87 | Ma WY et al (2012) | Variability in hemoglobin A1c predicts all-cause mortality in patients with type 2 diabetes | No funding |  |
| 88 | Marinho FS et al (2015) | Profile of disabilities and their associated factors in patients with type 2 diabetes evaluated by the Canadian occupational performance measure: the Rio De Janeiro type 2 diabetes cohort study. | 1. Conselho Nacional de Desenvolvimento Cientı ́fico e Tecnolo ́gico (CNPq) a 2. Fundac ̧a ̃o Carlos Chagas Filho de Amparo a Pesquisa do Estado do Rio de Janeiro (FAPERJ). | 1. Government 2. Government |
| 89 | Moehlecke M et al (2010) | Effect of metabolic syndrome and of its individual components on renal function of patients with type 2 diabetes mellitus. | No funding |  |
| 90 | Mohan V et al (2013) | Clinical profile of long-term survivors and nonsurvivors with type 2 diabetes. | No funding |  |
| 91 | Morris NS et al (2006) | Literacy and health outcomes: a cross-sectional study in 1002 adults with diabetes. | US National Institute of Diabetes and Digestive and Kidney Disease (R01 DK61167 and K24 DK68380). | Government |
| 92 | Muggeo M et al (2000) | Fasting Plasma Glucose Variability Predicts 10-Year Survival of Type 2 Diabetic Patients | 1. Italian National Research Council 2. Italian Ministry of University and Scientific and Technological Research 3. National Institute of Diabetes and Digestive and Kidney Diseases (NIDDK) 4. Regione Veneto 5. Astra, 6. Bayer 7. Boehringer Mannheim 8. Fournier Pierrel Pharma 9. Glaxo Wellcome 10. Laboratori Guidotti 11. Hoechst Marion Roussel 12. Servier Italia 13. A. Menarini 14. Merck Sharp & Dohme 15. Molteni Farmaceutici 16. Zeneca 17. Pfizer Italiana 18. Roering Farmaceutici Italiana 19. Parke-Davis 20. Novo Nordisk Farmaceutici 21. Neopharmed 22. LifeScan | 1-4. Government 5-22: Private industry |
| 93 | Nakhjavani M et al (2012) | Gender difference in albuminuria and ischemic heart disease in type 2 diabetes. | No funding |  |
| 94 | Nefs G et al (2015) | Depressive Symptom Clusters Differentially Predict Cardiovascular Hospitalization in People With Type 2 Diabetes. | ZonMW Grant from the Netherlands Organisation for Health Research and Development | Government |
| 95 | Nunes S et al (2019) | Three different phenotypes of mild nonproliferative diabetic retinopathy with different risks for development of clinically significant macular edema | Funda ̧ca ̃o para a Ciencia e a Tecnologia, Portugal, under the research project PTDC/SAU-OSM/72635/2006 | Foundation |
| 96 | O’Donnell A et al (2015) | Neighborhood social environment and patterns of depressive symptoms among patients with type 2 diabetes mellitus. | 1. American Heart Association Award #13GRNT17000021 2. National Institute of Mental Health R21 MH094940 3. National Institute of Mental Health R34 MH085880. | 1. Professional organization 2. Government 3. Government |
| 97 | Ogihara T et al (2017) | Relationships between lifestyle patterns and cardio-renal-metabolic parameters in patients with type 2 diabetes mellitus: A cross-sectional study. | Manpei Suzuki Diabetes Foundation (to T.M., Number 26-76). | Foundation |
| 98 | Okosun IS et al (2014) | Clustering of cardiometabolic risk factors and risk of elevated HbA1c in non-Hispanic White, non-Hispanic Black and Mexican-American adults with type 2 diabetes. | No funding |  |
| **S/No** | **Authors (Year)** | **Article name** | **Funding source** | **Funding source category** |
| 99 | Osborn CY et al (2010) | Racial disparities in the treatment of depression in low-income persons with diabetes. | 1. National Institutes of Health (Grants R01-CA-92447, HL-67715, and P01-DK-20593).  2. Diversity Supplement Award, National Institute of Diabetes and Digestive and Kidney Diseases (P60-DK-020593). | 1. Government 2. Government |
| 100 | Penno G et al (2013) | Gender differences in cardiovascular disease risk factors, treatments and complications in patients with type 2 diabetes: the RIACE Italian multicentre study. | 1. Eli-Lilly 2. Takeda 3. Chiesi Pharmaceuticals  4. Boehringer-Ingelheim 5. Research Foundation of the Italian Society of Diabetology and the Diabetes 6. Endocrinology and Metabolism Foundatio | 1. Private industry 2. Private industry 3. Private industry  4. Private industry 5. Foundation 6. Foundation |
| 101 | Penno G et al (2013) | HbA1c variability as an independent correlate of nephropathy, but not retinopathy, in patients with type 2 diabetes: the Renal Insufficiency And Cardiovascular Events (RIACE) Italian multicenter study. | 1. Research Foundation of the Italian Society of Diabetology (Fo.Ri.SID)  2. Diabetes, Endocrinology, and Metabolism (DEM) Foundation 3. Eli-Lilly 4. Takeda 5. Chiesi Farmaceutici 6. Boehringer Ingelheim. | 1. Foundation 2. Foundation 3. Private industry 4. Private industry 5. Private industry 6. Private industry |
| 102 | Prentice JC et al (2012) | Primary care and health outcomes among older patients with diabetes. | 1. Grant IAD-06-112 and IIR 04-233 from the Health Services Research and Development Service, Department of Veterans Affairs  2. Grant 62967 from the Health Care Financingand Organization Initiative under the Robert Wood Johnson Foundation. | 1. Government 2. Foundation |
| 103 | Rabi DM et al (2007) | Clinical and medication profiles stratified by household income in patients referred for diabetes care. | No funding |  |
| 104 | Ravera M et al (2009) | Chronic kidney disease and cardiovascular risk in hypertensive type 2 diabetics: a primary care perspective. | 1. Northern Ireland Kidney Research Fund 2. NHS Kidney Care. | 1. Government 2. Government |
| 105 | Ravona-Springer R et al (2014) | Trajectories in Glycemic Control over Time Are Associated with Cognitive Performance in Elderly Subjects with Type 2 Diabetes | 1. The American Federation for Aging Research (AFAR), Young investigator award 2011  2. Alzheimer’s Association 3. Helen Bader Foundation and the Irma T. Hirschl Scholar Award  4. Leroy Schecter Foundation Award  5. National Institute of Aging  6. United States Department of Veterans Affairs 7. Berkman Charitable Trust. | 1. Government 2. Professional organizations 3. Foundation 4. Foundation 5. Government 6. Government 7. Foundation |
| 106 | Rosa MQM et al (2017) | Disease and Economic Burden of Hospitalizations Attributable to Diabetes Mellitus and Its Complications: A Nationwide Study in Brazil. | 1. Brazilian Ministry of Health through the National Health Fund (Process # 25000.105417/2014-01) 2. The Brazilian Institute of Health Technology (IATS)/National Council for Scientific and Technological Development (CNPq) supported the open access publication costs. | 1. Government 2. Government |
| 107 | Safai N et al (2018) | Stratification of type 2 diabetes based on routine clinical markers | Research grant from Innovation Fund Denmark (grant number: 4135-00028B) | Government |
| 108 | Sakurai T et al (2014) | Differential subtypes of diabetic older adults diagnosed with Alzheimer's disease. | 1. Chojyu (24-24, 25-6) 2. Ministry of Education, Culture, Sports, Science, and Technology (22590654) 3. Ministry of Health, Labor and Welfare (H25- Ninchisho-008), Japan. | 1. Government 2. Government 3. Government |
| 109 | Sancho-Mestre C et al (2016) | Pharmaceutical cost and multimorbidity with type 2 diabetes mellitus using electronic health record data. | No funding |  |
| **S/No** | **Authors (Year)** | **Article name** | **Funding source** | **Funding source category** |
| 110 | Sarmento RA et al (2018) | Eating patterns and health outcomes in patients with type 2 diabetes | 1. Fundo de Incentivo `a Pesquisa e Eventos–Hospital de Clı ́nicas de Porto Alegre.  2. Scholarships from the Fundaç~ao de Aperfeiçoamento de Pessoal de Nı ́vel Superior 3. Scholarship from the Programa Institucional de Bolsas de Iniciaç~ao Cientı ́fica–Conselho Nacional de Desenvolvimento Cientı ́fico e Tecnol ́ogico. | 1. Government 2. Government 3. Government |
| 111 | Schäfer I et al (2013) | Identifying groups of nonparticipants in type 2 diabetes mellitus education. | 1. German Federal Ministry of Education and Research (grant number 01GK0714)  2. German Medical Association within their peer-reviewed Funding Initiative for Health Services Research (grant number 06-152). | 1. Government 2. Professional organization |
| 112 | Schillinger D et al (2002) | Association of health literacy with diabetes outcomes. | 1. University of California, San Francisco, Hell- man Family Early Career Research Award  2. the Pfizer, Inc, Health Literacy Research Award 3. Agency for Health Research and Quality (AHRQ) grant DII 99187-1, AHRQ center grant P01HS/10856 4. General Clinical Research Center at San Francisco General Hospital through grant 5M01-RR00084-30 from the National Institutes of Health’s National Center for Research Resources. | 1. Professional Organizations 2. Private industry 3. Professional Organizations 4. Government |
| 113 | Seok H at al (2013) | Clinical characteristics and insulin independence of Koreans with new-onset type 2 diabetes presenting with diabetic ketoacidosis. | No funding |  |
| 114 | Shamshirgaran SM et al (2017) | Age differences in diabetes-related complications and glycemic control. | Research Council, Tabriz University of Medical Science. | Professional Organizations |
| 115 | Sheu SJ et al (2012) | High HbA1c level was the most important factor associated with prevalence of diabetic retinopathy in Taiwanese type II diabetic patients with a fixed duration. | 1. Grant NSC97-2314-B-075B-011 from the National Science Council  2. Grant VGHKS100-065 from the Kaohsiung Veterans General Hospital, Kaohsiung City, Taiwan. | 1. Government 2. Government |
| 116 | Siaw MY et al (2016) | Metabolic parameters in type 2 diabetic patients with varying degrees of glycemic control during Ramadan: An observational study. | No funding |  |
| 117 | Sidorenkov G et al (2018) | HbA1c response after insulin initiation in patients with type 2 diabetes mellitus in real life practice: Identifying distinct subgroups. | Department of Clinical Pharmacy and Pharmacology, University Medical Center Groningen, University of Groningen, The Netherlands. | Professional organization |
| 118 | Solini A et al (2014) | Resistant hypertension in patients with type 2 diabetes: clinical correlates and association with complications. | 1. Research Foundation of the Italian Society of Diabetology  2.Diabetes, Endocrinology and Metabolism Foundation 3. Eli-Lilly 4. Takeda 5. Chiesi Farmaceutici  6. Boehringer-Ingelheim. | 1. Foundation 2. Foundation 3. Private industry 4. Private industry 5. Private industry 6. Private industry |
| **S/No** | **Authors (Year)** | **Article name** | **Funding source** | **Funding source category** |
| 119 | Spauwen PJJ et al (2016) | Lower verbal intelligence is associated with diabetic complications and slower walking speed in people with type 2 diabetes: the Maastricht Study | 1. European Regional Development Fund via OP-Zuid 2. The Province of Limburg, the Dutch Ministry of Economic Affairs (grant 31O.041) 3. Stichting De Weijerhorst (Maastricht, the Netherlands) 4. Tje Pearl String Initiative Diabetes (Amsterdam, the Netherlands) 5. Cardiovascular Center (CVC, Maastricht, the Netherlands) 6. Cardiovascular Research Institute Maastricht (CARIM, Maastricht, the Netherlands),  7. School for Public Health and Primary Care (CAPHRI, Maastricht, the Netherlands) 8. School for Nutrition, Toxicology and Metabolism (NUTRIM, Maastricht, the Netherlands) 9. Stichting Annadal (Maastricht, the Netherlands) 10. Health Foundation Limburg (Maastricht, the Netherlands)  11. Janssen-Cileg B.V. (Tilburg, the Netherlands) 12. Novo Nordisk Farma B.V. (Alphen aan den Rijn, the Netherlands)  13. Sanofi-Aventis Netherlands B.V. (Gouda, the Netherlands) | 1-6: Government 7-8: Professional organization 9-10: Goverment 11-13: Private industry |
| 120 | Tan ED et al (2016) | Changes in characteristics and management of Asian and Anglo-Celts with type 2 diabetes over a 15-year period in an urban Australian community: The Fremantle Diabetes Study. | 1. Raine Foundation, University of Western Australia.  2. Project grants from the Australian National Health and Medical Research Council (NHMRC; no. 513781 and 1042231).  3. National Heart Foundation Student Summer Scholarship 4. NHMRC Practitioner Fellowship. | 1. Foundation 2. Government 3. Foundation 4. Government |
| 121 | Tao X et al (2016) | Association between socioeconomic status and metabolic control and diabetes complications: a cross-sectional nationwide study in Chinese adults with type 2 diabetes mellitus. | No funding |  |
| 122 | Toh MPHS et al (2011) | Association of Younger Age With Poor Glycemic and Cholesterol Control in Asians With Type 2 Diabetes Mellitus in Singapore | No funding |  |
| 123 | Twito O et al (2013) | New-onset diabetes in elderly subjects: association between HbA1c levels, mortality, and coronary revascularization. | No funding |  |
| 124 | Ustulin M et al (2017) | Characteristics of frequent emergency department users with type 2 diabetes mellitus in Korea. | Kyung Hee University | Professional organizations |
| 125 | van Dijk CE et al (2013) | Type II diabetes patients in primary care: profiles of healthcare utilization obtained from observational data. | No funding |  |
| 126 | Vepsäläinen T et al (2011) | Physical activity, high-sensitivity C-reactive protein, and total and cardiovascular disease mortality in type 2 diabetes. | No funding |  |
| 127 | Vitale M et al (2016) | Sex differences in food choices, adherence to dietary recommendations and plasma lipid profile in type 2 diabetes - The TOSCA.IT study. | 1. Italian Medicines Agency (AIFA) within the Independent Drug Research Program e contract N. FARM6T9CET e  2. Diabete Ricerca, the no profit Research Foundation of the Italian Diabetes Society. | 1. Government 2. Foundation |
| 128 | Walker JJ et al (2011) | Effect of socioeconomic status on mortality among people with type 2 diabetes: a study from the Scottish Diabetes Research Network Epidemiology Group. | 1. Scottish Government 2. NHS Research Scotland (NRS), through the Scottish Diabetes Research Network. | 1. Government 2. Government |
| 129 | Walraven I et al (2014) | Distinct HbA1c trajectories in a type 2 diabetes cohort. | Lilly Diabetes | Private industry |
| 130 | Wan EY et al (2017) | Association of Visit-to-Visit Variability of Systolic Blood Pressure With Cardiovascular Disease and Mortality in Primary Care Chinese Patients With Type 2 Diabetes-A Retrospective Population-Based Cohort Study. | 1. Hong Kong HA (Reference no. 8011014157) 2. Health and Health Services Research Fund,FoodandHealth Bureau,HKSAR Commissioned Research on Enhanced Primary Care Study (Reference no. EPC-HKU-2). | 1. Professional organization 2. Government |
| 131 | Wang RH et al (2013) | Determinants for quality of life trajectory patterns in patients with type 2 diabetes. | National Science Council, Taiwan | Government |
| 132 | Wang X et al (2017) | Identifying Patterns of Lifestyle Behaviors among People with Type 2 Diabetes in Tianjin, China: A Latent Class Analysis | 1. National Natural Science Foundation of China (71373175) 2. Tianjin Municipal Human Resources and Social Security Bureau (TMIR 201502) 3. Ministry of Education of Humanities and Social Science project (17YJAZH048). | 1. Government 2. Foundation 3. Foundation |
| **S/No** | **Authors (Year)** | **Article name** | **Funding source** | **Funding source category** |
| 133 | Wang Y et al (2003) | Phenotypic heterogeneity and associations of two aldose reductase gene polymorphisms with nephropathy and retinopathy in type 2 diabetes. | 1. Chinese University of Hong Kong Strategic Grant 2. Hong Kong Research Grants Committee Earmarked Grants 3. Hong Kong Innovation and Technology Fund (ITS/033/00). | 1. Professional Organizations 2. Government 3. Government |
| 134 | Wang Y et al (2014) | Racial disparities in cardiovascular risk factor control in an underinsured population with Type 2 diabetes. | Louisiana State University’s Improving Clinical Outcomes Network (LSU ICON). | Professional organization |
| 135 | Wolffenbuttel BH et al (2013) | Ethnic differences in glycemic markers in patients with type 2 diabetes. | Eli Lilly and Company | Private industry |
| 136 | Won JC et al (2017) | Clinical Phenotype of Diabetic Peripheral Neuropathy and Relation to Symptom Patterns: Cluster and Factor Analysis in Patients with Type 2 Diabetes in Korea | 2016 Inje University research grant | Professional organizations |
| 137 | Xu D et al (2016) | Fasting plasma glucose variability and all-cause mortality among type 2 diabetes patients: a dynamic cohort study in Shanghai, China. | 1. Research Fund from Shanghai Municipal Commission of Health and Family Planning (NO: SH201415)  2. Grant for Young Investigators of Minhang district, Shanghai. | 1. Governement 2. Government |
| 138 | Xu ZR et al (2001) | Clustering of cardiovascular risk factors with diabetes in Chinese patients: the effects of sex and hyperinsulinaemia. | No funding |  |
| 139 | Yang W et al (2016) | Clinical Characteristics of Young Type 2 Diabetes Patients with Atherosclerosis. | 1. National High Technology Research and Development Program of China (2012AA02A509) 2. National Key Basic Research Program of China (2011CB504000) 3. Beijing Science and Technology Committee Funding (D131100005313008). | 1. Government 2. Government 3. Professional Organizations |
| 140 | Yeung RO et al (2014) | Metabolic profiles and treatment gaps in young-onset type 2 diabetes in Asia (the JADE programme): a cross-sectional study of a prospective cohort. | 1. The Asia Diabetes Foundation (ADF) 2. Merck. | 1. Foundation 2. Private industry |
| 141 | Yeung RO et al (2018) | Determinants of hospitalization in Chinese patients with type 2 diabetes receiving a peer support intervention and JADE integrated care: The PEARL randomised controlled trial | 1. American Academy of Family Physicians Foundation Peers for Progress Program through the Eli Lilly and Company Foundation awarded to the Asia Diabetes Foundation (ADF) 2. Merck educational grant.  3. University of British Columbia Clinical Investigator Program  4. National Natural Science Foundation of China | 1. Private industry 2. Private industry 3. Professional organization 4. Foundation |
| 142 | Yoda N et al (2008) | Classification of adult patients with type 2 diabetes using the Temperament and Character Inventory. | No funding |  |
| 143 | Zhang XL et al (2018) | The effects of cardiovascular risk factor combined anti-platelet therapy and the risk of cerebrovascular events in patients with T2DM in an urban community over 96-months follow-up: The Beijing communities diabetes study 19. | Grant of Special Scientific Research on Capital Health Development (2016-1-2057, 2016-2-2054), Beijing Municipal Science &Technology Commission (Z151100004015021), | Government |
| 144 | Zhao W et al (2014) | Sex differences in the risk of stroke and HbA(1c) among diabetic patients. | Louisiana State University’s Improving Clinical Outcomes Network (LSU ICON) | Professional organization |
| 145 | Zheng W et al (2011) | Factor analysis of diabetic nephropathy in Chinese patients | No funding |  |
| 146 | Zhou X et al (2016) | Prevalence of Obesity and Its Influence on Achievement of Cardiometabolic Therapeutic Goals in Chinese Type 2 Diabetes Patients: An Analysis of the Nationwide, Cross-Sectional 3B Study. | Research grant (#39103) from MSD China Holding Co., Ltd | Private industry |
| 147 | Zinman B et al (2004) | Phenotypic characteristics of GAD antibody-positive recently diagnosed patients with type 2 diabetes in North America and Europe. | GlaxoSmithKline | Private industry |
| 148 | Zou X et al (2017) | The characteristics of newly diagnosed adult early-onset diabetes: a population-based cross-sectional study. | 1. Chinese Medical Association Foundation and Chinese Diabetes Society, 2. National Key Technologies R&D Program of China (2009BAI80B02) 3. National High-Tech R&D Program of China (863 Program 2012AA02A509). | 1. Foundation 2. Government 3. Government |
