# Supplementary material for: Population segmentation of type 2 diabetes mellitus patients and its clinical applications - a scoping review
Source: BMC Med Res Methodol. 2021 Mar 11;21:49. doi: 10.1186/s12874-021-01209-w (PMC7953703; doi:10.1186/s12874-021-01209-w)
Supplement: Supplementary file 4 — Additional file 4. Advantages and disadvantages of population segmentation methods [14, 181] [file 12874_2021_1209_MOESM4_ESM.docx]

**Supplementary File 4 Advantages and disadvantages of population segmentation methods (15, 182)**

| **Methodology** | **Advantages** | **Disadvantages** |
| --- | --- | --- |
| ***Expert-based population segmentation*** |  |  |
| Judgemental splitting | - Simple to use | - “Non-objective” as the objective assessment of the discriminatory properties of the segmentation variable(s) have not be performed |
| Prescribed binning criteria | - Produce manageable number of patient segments | - “Non-objective” as expert opinion may not offer good discriminatory properties of derived segmentation solutions. |
| ***Data-driven population segmentation*** |  |  |
| Cluster based analyses |  |  |
| - Latent class / growth analyses | - Missing data can be handled - Statistical measures can be used to evaluate model fit and determine optimal number of patient segments e.g. Akaike Information criterion, Bayesion Information criterion - Ability to handle continuous and categorical variables - Variables do not need to be standardised | - Analysis of large patient datasets can be computationally intensive |
| - K-means cluster analyses | - Can manage large patient datasets - Ability to manage both continuous and categorical variables | - Difficulty in generating reproducible patient segments for different outcomes as that initial centroid for each cluster is random - Affected by outliers - Lack of statistical measures such as AIC and BIC |
| - Hierarchical analyses | - Availability of rules (e.g. Duda’s pseudo T square statistic, Calinski’s pseudo F statistic) to achieve optimal number of clusters - Ability to handle continuous, nominal and categorical variables - Provision of dendogram for ease of visualisation of derived segmentation solutions | - Difficulty to managing large datasets exceeding 1000 patients (optimal patient population size: 300-400) - Affected by outliers |
| Decision trees | - Provide objective and derived approach to population segmentation - Ability to manage outliers and missing data - Fast | - Potential propagation of errors made in early stages of splitting |
