# Supplementary material for: Population segmentation of type 2 diabetes mellitus patients and its clinical applications - a scoping review
Source: BMC Med Res Methodol. 2021 Mar 11;21:49. doi: 10.1186/s12874-021-01209-w (PMC7953703; doi:10.1186/s12874-021-01209-w)
Supplement: Supplementary file 5 — Additional file 5. Definitions of criterion used for evaluation of population segmentation outcomes [16, 181] [file 12874_2021_1209_MOESM5_ESM.docx]

**Supplementary File 5** Definitions of criterion used for evaluation of population segmentation results (17, 182)

| **Criteria** | **Details** |
| --- | --- |
| Number of patient segments | The final number of patient segments derived from population segmentation strategies should be reported. |
| Internal validation | Refers to the performance properties of the derived patient segment structure by utilizing the dataset that was used to derive the segment structure |
| External validation | Refers to the performance properties of the derived patient segment structure by utilizing an external dataset or patient population |
| Identifiability and interpretability | Derived patient segments should be recognized easily to facilitate clinical interpretation |
| Substantiality | Derived patient segments should be of a sufficient size to facilitate design of intervention or formulation of targeted policies |
| Stability | Derived patient segment should be relatively stable over time. |
| Actionability | Each derived patient segment should facilitate the design of distinctive healthcare intervention strategies to target its patients |
